# Integrative analysis identifies key mRNA biomarkers for diagnosis, prognosis, and therapeutic targets of HCV-associated hepatocellular carcinoma

**DOI:** 10.18632/aging.202957

**Published:** 2021-05-04

**Authors:** Yongqiang Zhang, Yuqin Tang, Chengbin Guo, Gen Li

**Affiliations:** 1 Molecular Medicine Center, West China Hospital, Sichuan University, Chengdu 610041, P.R. China; 2 West China School of Medicine, West China Hospital, Sichuan University, Chengdu 610041, P.R. China; 3 School of Basic Medical Sciences, Chengdu University of Traditional Chinese Medicine, Chengdu 611137, P.R. China; 4 Guangzhou Women and Children’s Medical Center, Guangzhou Medical University, Guangzhou 510623, P.R. China

**Keywords:** hepatocellular carcinomas, hepatitis C virus, differentially expressed genes, biomarkers, WGCNA

## Abstract

Hepatitis C virus-associated HCC (HCV-HCC) is a prevalent malignancy worldwide and the molecular mechanisms are still elusive. Here, we screened 240 differentially expressed genes (DEGs) of HCV-HCC from Gene expression omnibus (GEO) and the Cancer Genome Atlas (TCGA), followed by weighted gene coexpression network analysis (WGCNA) to identify the most significant module correlated with the overall survival. 10 hub genes (CCNB1, AURKA, TOP2A, NEK2, CENPF, NUF2, CDKN3, PRC1, ASPM, RACGAP1) were identified by four approaches (Protein-protein interaction networks of the DEGs and of the significant module by WGCNA, and diagnostic and prognostic values), and their abnormal expressions, diagnostic values, and prognostic values were successfully verified. A four hub gene-based prognostic signature was built using the least absolute shrinkage and selection operator (LASSO) algorithm and a multivariate Cox regression model with the ICGC-LIRI-JP cohort (N =112). Kaplan-Meier survival plots (*P* = 0.0003) and Receiver Operating Characteristic curves (ROC = 0.778) demonstrated the excellent predictive potential for the prognosis of HCV-HCC. Additionally, upstream regulators including transcription factors and miRNAs of hub genes were predicted, and candidate drugs or herbs were identified. These findings provide a firm basis for the exploration of the molecular mechanism and further clinical biomarkers development of HCV-HCC.

## INTRODUCTION

Globally, Hepatocellular carcinoma (HCC) represents the predominant histological form of liver cancer (accounting for 75%-85% of all cases), which is a commonly diagnosed malignancy with an increasing incidence rate and ranked fourth in mortality among all cancers [1]. In 2018, HCC leads to more than 781,000 deaths and about 841,000 newly diagnosed cases all over the world [1]. Hepatitis C virus (HCV) infection is one of the major causes of HCC, especially in western countries and Japan [1]. According to a survey of 227,808 participants, the anti-HCV-positive rate was 3.0%, but more than 60% of the participants were not aware of their infection [2]. While the introduction of the vaccine has reduced the prevalence of Hepatitis B virus (HBV) infection with promise to decrease the incidence of HBV- associated HCC (HBV-HCC) in certain high-risk countries, there is no vaccine available for HCV infection [1]. On the other hand, although great advances have been achieved for the investigation of HCC in the last decades, its underlying mechanisms of different etiologies vary dramatically, therefore extensive efforts are still needed to establish a better understanding of carcinogenesis and pathogenesis of HCV- associated HCC (HCV-HCC).

Recently, a growing number of candidate biomarkers for diagnosis or prognosis of HCC have been identified [3–12], among which the most commonly reported biomarkers are dysregulated genes [3, 6, 11], significant members of a certain gene family or gene set [4, 10], potential CpG methylation status [7, 9], and alternative splicing signatures [5, 12]. For example, a 24-mRNA-based risk signature has been developed as an independent risk classifier for the prediction of early recurrence in HCC patients [6]. Similarly, a nine immune-related mRNA signature was generated to predict the overall survival (OS) of HCC [10]. While most of the studies focused on HCC prognosis, its diagnosis has not yet been fully investigated. Besides, few studies characterized the stratified categorization by different risk factors (especially HCV infection), however, they may exert contrary outcomes even for the same risk group. Thus, additional markers are required for a more accurate risk prediction in HCV-HCC patients.

**Figure 1:**
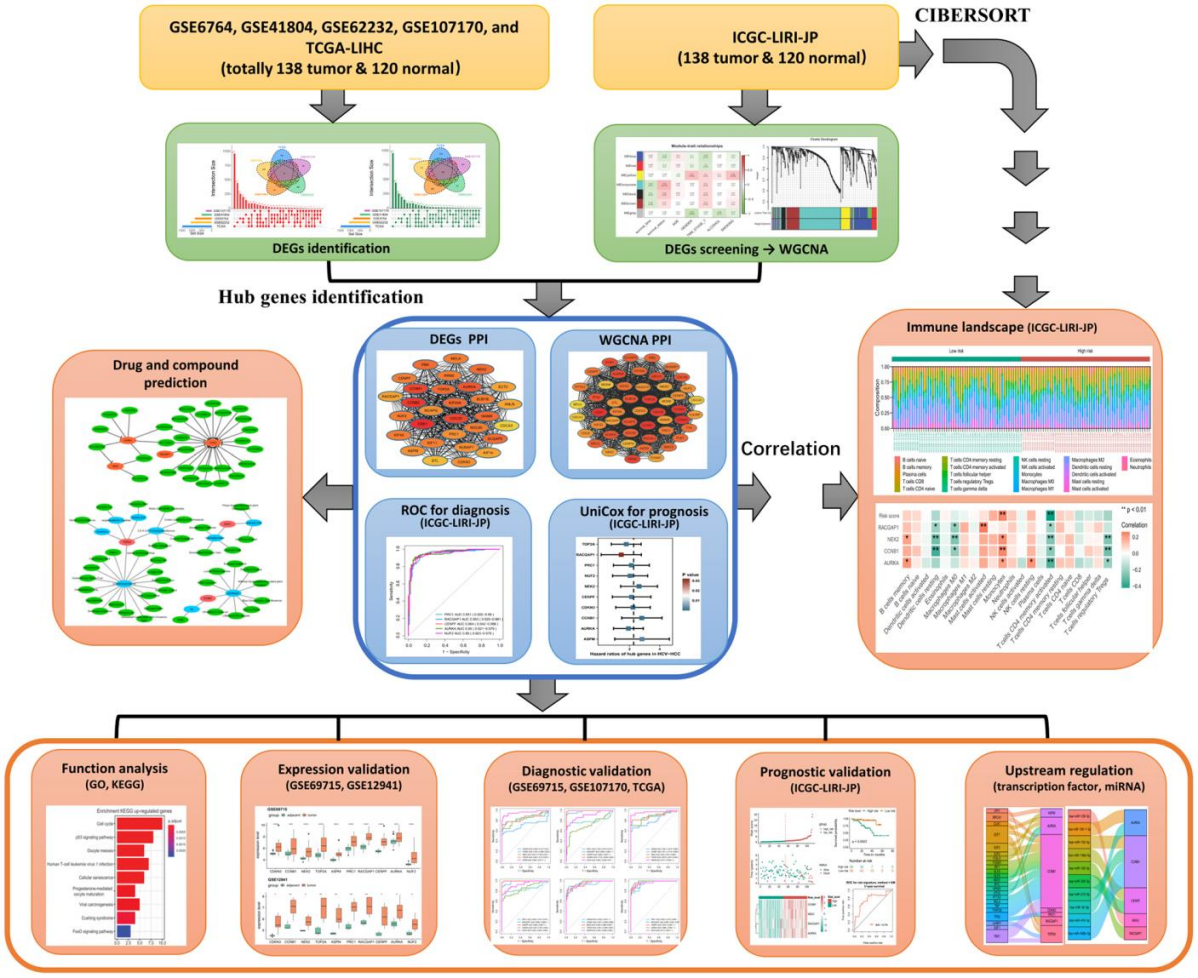
Flowchart of this study.

Of note, single cohort-based studies may result in false-positive outcomes because of the small sample size and limitation of technology platforms. Therefore, an integrated analysis combining multiple public databases such as The Cancer Genome Atlas (TCGA), The Gene Expression Omnibus (GEO), and International Cancer Genome Consortium (ICGC) could improve the accuracy and reliability of the results tremendously, providing an effective approach for the exploration of molecular landscape and the discovery of potential therapeutic targets or important biomarkers for diagnosis and prognosis of cancer. Thus, with the aim to identify the candidate crucial genes for diagnosis and prognosis of HCV-HCC from multiple public databases, which might also give a clue for seeking therapeutic targets in HCV-HCC, we enrolled eight gene expression datasets from TCGA, GEO, and ICGC, including a total of 304 HCV-HCC samples and 290 adjacent normal tissues in the present study. 240 differentially expressed genes (DEGs) were screened in the first step, followed by the identification of 10 hub genes with a combined analysis. Then, the diagnostic and prognostic values of these hub genes were verified. The least absolute shrinkage and selection operator (LASSO)-based penalized Cox regression (LASSO-COX) was performed to construct a prognostic risk signature, which was further evaluated by Kaplan-Meier curves and ROC plots. The relationships between the risk signature and tumor infiltration immune cells were also determined by Spearman correlation analysis. Moreover, Upstream regulations of the 10 hub genes including miRNAs and transcription factors were also predicted. At last, network pharmacological analysis was conducted to seek possible small molecular drugs for HCV-HCC. Collectively, this study identified 10 hub genes concerning the crucial roles in the carcinogenesis of HCV-HCC, which may provide a firm basis for understanding the transcriptional regulatory mechanisms and advancing studies in clinical biomarker discovery of HCV-HCC. The flowchart summarizing the general process of this study was shown in Figure 1.

## RESULTS

### Screening of robust DEGs in HCV-HCC

**Figure 2:**
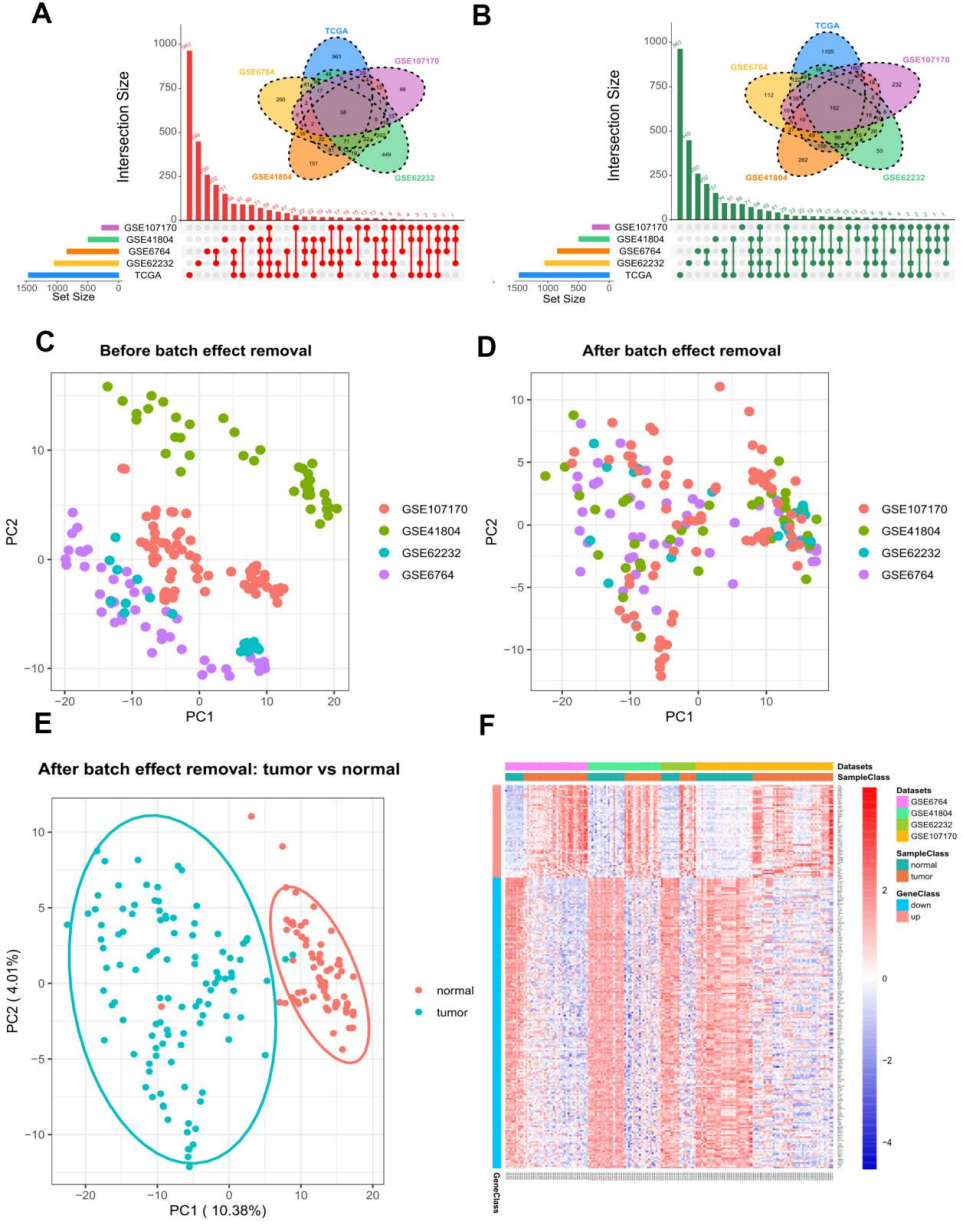
**Differential gene expression between HCV-HCC tumor and adjacent normal tissues.** (**A**, **B**) The combination of Venn plot and Upset plot showing the common upregulated genes (**A**) and the common downregulated genes (**B**) in HCV-HCC according to five public datasets. The screening criteria was set as |log Fold change (FC)| > 1 and FDR (adj.P.Val) <0.05. (**C**, **D**) Principal component analysis (PCA) for the gene expression profiles from four microarray datasets before (**C**) and after (**D**) batch effect removal. The colors represent different datasets. (**E**) scatter plots visualizing the identified clusters of the tumor and normal samples based on the combined dataset. (**F**) Heatmap of the 240 DEGs showing their expression values for each patient. The scale bar indicates the gene expression value. Red indicates high expression level, and blue indicates low expression level. HCV-HCC, HCV- associated HCC. DEGs, differentially expressed genes.

By using GEO2R and the screening criteria of |log Fold change (FC)| > 1 and FDR (adj.P.Val) <0.05, we extracted 1722 DEGs (842 upregulated and 880 downregulated) from GSE6764, 1459 DEGs (496 upregulated and 963 downregulated) from GSE41804, 1761 DEGs (1050 upregulated and 711 downregulated) from GSE62232, and 1163 DEGs (276 upregulated and 887 downregulated) from GSE107170. In the TCGA dataset, we fetched 3740 DEGs (1468 upregulated and 2272 downregulated) between HCV-HCC and normal tissues with the same threshold. As shown in Figure 2A, 2B, a total of 240 overlapping DEGs were identified, including 58 commonly upregulated genes, and 182 commonly downregulated genes. To increase the robustness of these common DEGs, we integrated the four microarray datasets into a combined dataset. The Combat function embedded in sva package was used to remove the batch effect. Plots of the Principal component analysis (PCA) indicated that after expression normalization, the batch effect was all removed successfully (Figure 2C, 2D). In addition, tumor samples and normal samples were clustered independently after batch removal (Figure 2E). Differential analysis by limma package revealed that all the 240 DEGs were still significant in the combined dataset (Figure 2F and Supplementary Table 2).

### Co-expression network construction and identification of the most important module

**Figure 3:**
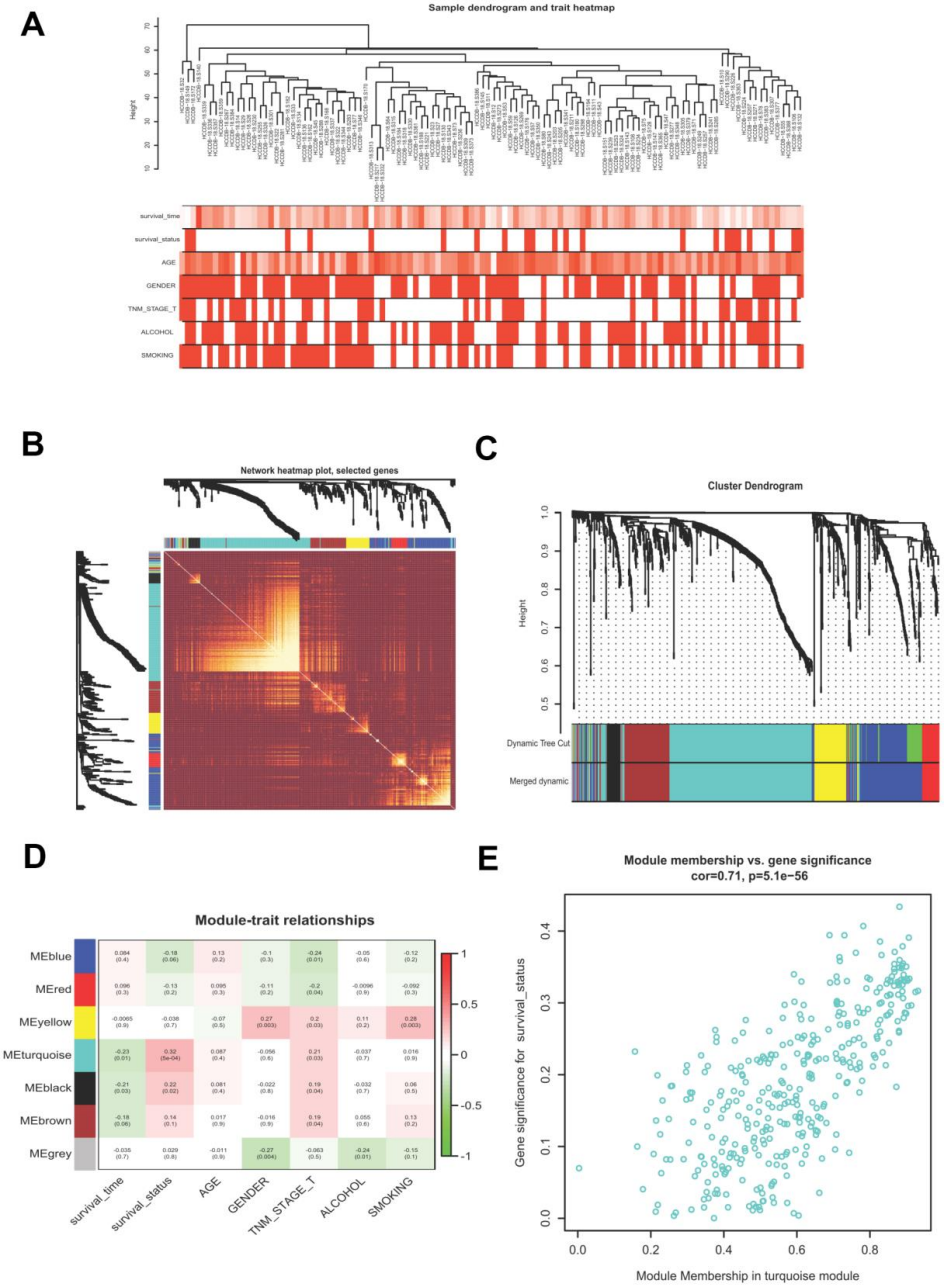
**Building a WGCNA network to identify the most significant module correlated with survival status.** (**A**) Sample clustering tree with clinical traits. (**B**) Heatmap showing the eigengene networks according to the topological overlap matrix (TOM) based dissimilarity. (**C**) Gene clustering dendrogram, with each color corresponding to an individual gene module. (**D**) Pearson correlation analysis between module eigengenes and clinical traits. (**E**) scatter plot showing the gene significance (GS) vs module membership (MM) for the turquoise module. WGCNA, Weight Gene Co-expression Network Analysis.

WGCNA is a useful approach to uncover gene expression patterns and to identify significant gene modules from multiple samples. We conducted WGCNA to disclose the most important module associated with HCV-HCC survival status. Briefly, 807 DEGs of the ICGC-LIRI-JP dataset were filtered (Supplementary Table 3), which were used to evaluate the outlier samples through sample hierarchical clustering using the average linkage method (Figure 3A). After the filtration, we obtained the adjacency matrix by using the appropriate soft threshold of 5 (scale-free R^2^ = 0.87), which was transformed into the TOM, and transited into the dissTOM, followed by the accomplishment of the gene clustering dendrogram and module identification (Figure 3B). Highly similar modules were then merged by the cut line of 0.3. Seven modules were remained (Figure 3C). The Pearson correlation heatmap showed the turquoise module including 357 DEGs has the most significant correlation with survival status and thus was selected for further study (Figure 3D). Figure 3E presented the GS and MM for each gene in the turquoise module.

### PPI network construction

**Figure 4:**
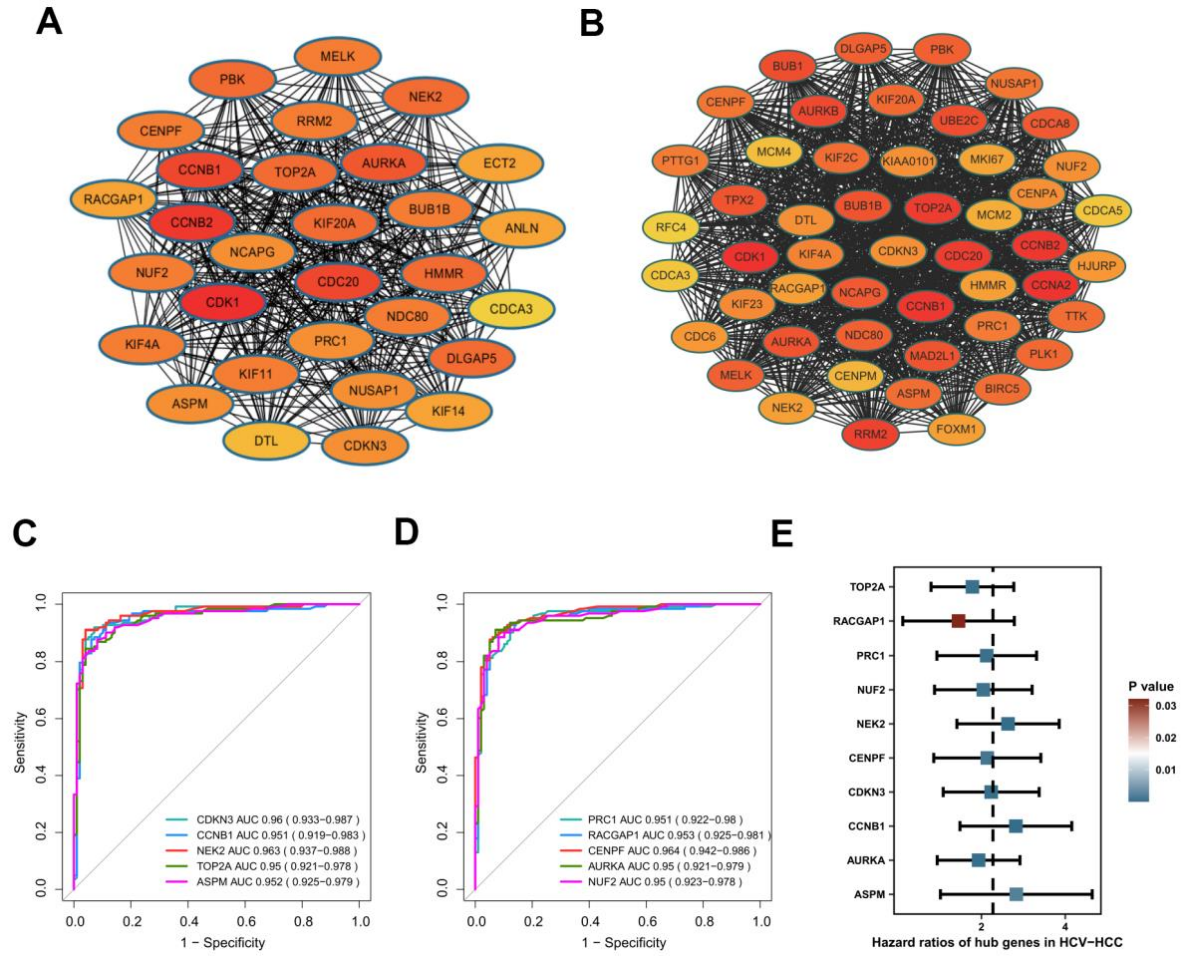
**Identification of hub genes in HCV-HCC.** (**A**) The most significant cluster identified from the DEGs-PPI network. (**B**) The WGCNA-PPI network constructed by the turquoise module. (**C**, **D**) ROC curves showing the AUROC scores and AUC (95%CI) of the 10 hub genes for discriminating tumor from normal samples based on the ICGC-LIRI-JP dataset. Colored lines indicate the ROC curve for each hub gene, and the grey line indicates the reference line. (**E**) Forest plot presenting the results of the univariate Cox regression analysis for the 10 hub genes. HCV-HCC, HCV- associated HCC. DEGs, differentially expressed genes. PPI, protein-protein interaction. WGCNA, Weight Gene Co-expression Network Analysis. ROC, receiver operating characteristic. 95%CI, 95% confidence interval.

We constructed a PPI network with the 240 overlapping DEGs using the STRING online database and the Cytoscape software (Supplementary Figure 1). The network gave 129 nodes and 585 edges, and showed 41 upregulated genes and 88 downregulated genes. The average number of neighbors was 9.07 and the clustering coefficient was 0.461. Using the MCODE app, a significant sub-cluster was screened out with a cluster score of 29.5, comprising 30 nodes and 428 edges (Figure 4A). Interestingly, all of the 30 genes showed high degrees of connectivity by cytohuber analysis (>20 for all cluster genes), indicating their potential hub roles for HCV-HCC tumorigenesis.

Besides, the 357 genes in the turquoise module by WGCNA were also used to construct a PPI network to identify candidate hub genes. The WGCNA-PPI network was composed of 245 nodes and 2581 edges (Figure 4B). There were 50 genes satisfied with the degree cutoff of ≥50 and defined as WGCNA-PPI-hub genes.

### Hub genes identification

Based on the 30 DEGs-PPI-hub genes and the 50 WGCNA-PPI-hub genes, we preliminarily obtained a total of 26 overlapping genes (data not shown). Then we evaluated the AUROC scores of the 26 genes for discriminating HCV-HCC from normal tissue samples using the ICGC-LIRI-JP dataset. As a result, 10 genes (CCNB1, AURKA, TOP2A, NEK2, CENPF, NUF2, CDKN3, PRC1, ASPM, RACGAP1) showed superior discriminatory abilities with AUROC scores of ≥0.95 (Figure 4C, 4D), suggesting their excellent diagnostic values. More importantly, all of the 10 genes were also revealed significantly associated with the overall survival outcome of HCV-HCC patients by UniCox analysis, indicating their potential prognostic powers in clinical use (Figure 4E). Thus, we consider these 10 genes as hub genes in HCV-HCC.

### Functional enrichment analysis

**Figure 5:**
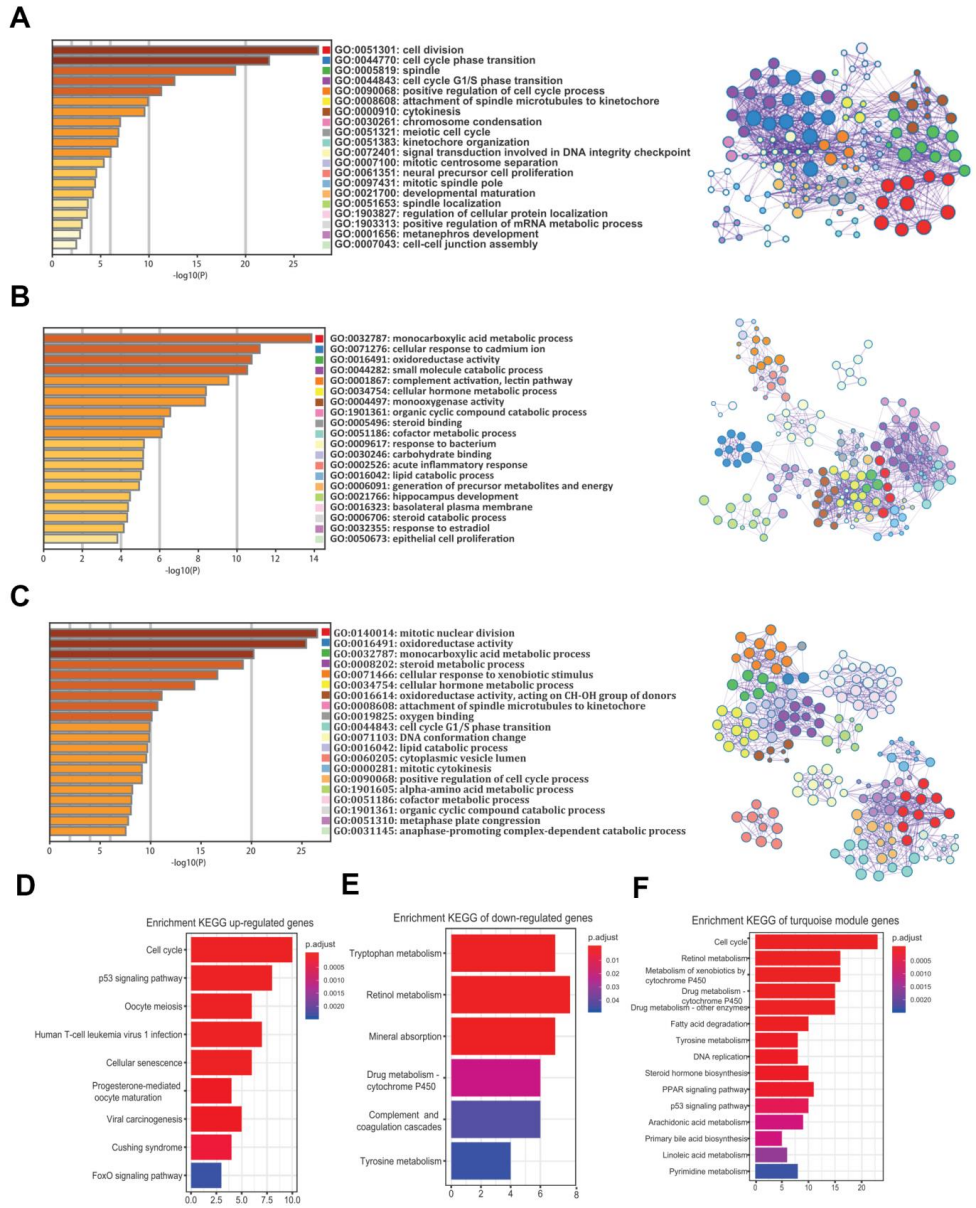
**GO and KEGG analysis of the 240 common DEGs and the turquoise module.** (**A**–**C**) GO enrichment analysis for the upregulated genes (**A**), downregulated genes (**B**), and the turquoise module (**C**) (Top 20 are shown). (**D**–**F**) Enrichment of KEGG pathways for the upregulated genes (**D**), downregulated genes (**E**), and the turquoise module (**F**). GO, gene ontology. KEGG, Kyoto Encyclopedia of Genes and Genomes. DEGs, differentially expressed genes.

To understand the biological functions of the robust DEGs and the turquoise module in HCV-HCC, we performed GO and KEGG analysis. GO enrichment analysis revealed that the commonly 58 upregulated genes were mostly involved in cell division, cell cycle phase transition, spindle, and other important GO terms, mainly related to cell proliferation (Figure 5A). The 182 commonly downregulated genes were mainly related to the monocarboxylic acid metabolic process, cellular response to cadmium ion, and oxidoreductase activity (Figure 5B). For the 357 genes in the turquoise module, mitotic nuclear division, oxidoreductase activity, and monocarboxylic acid metabolic process were the top GO terms (Figure 5C). On the other hand, KEGG analysis suggested that the most KEGG pathways associated with the upregulated genes were cell cycle, p53 signaling pathway, and oocyte meiosis (Figure 5D), while the tryptophan metabolism, retinol metabolism, and mineral absorption were the top three pathways for the downregulated genes (Figure 5E). Moreover, the turquoise module was mostly associated with cell cycle, retinol metabolism, and metabolism of xenobiotics by cytochrome P450 (Figure 5F).

### Hub genes expression validation

**Figure 6:**
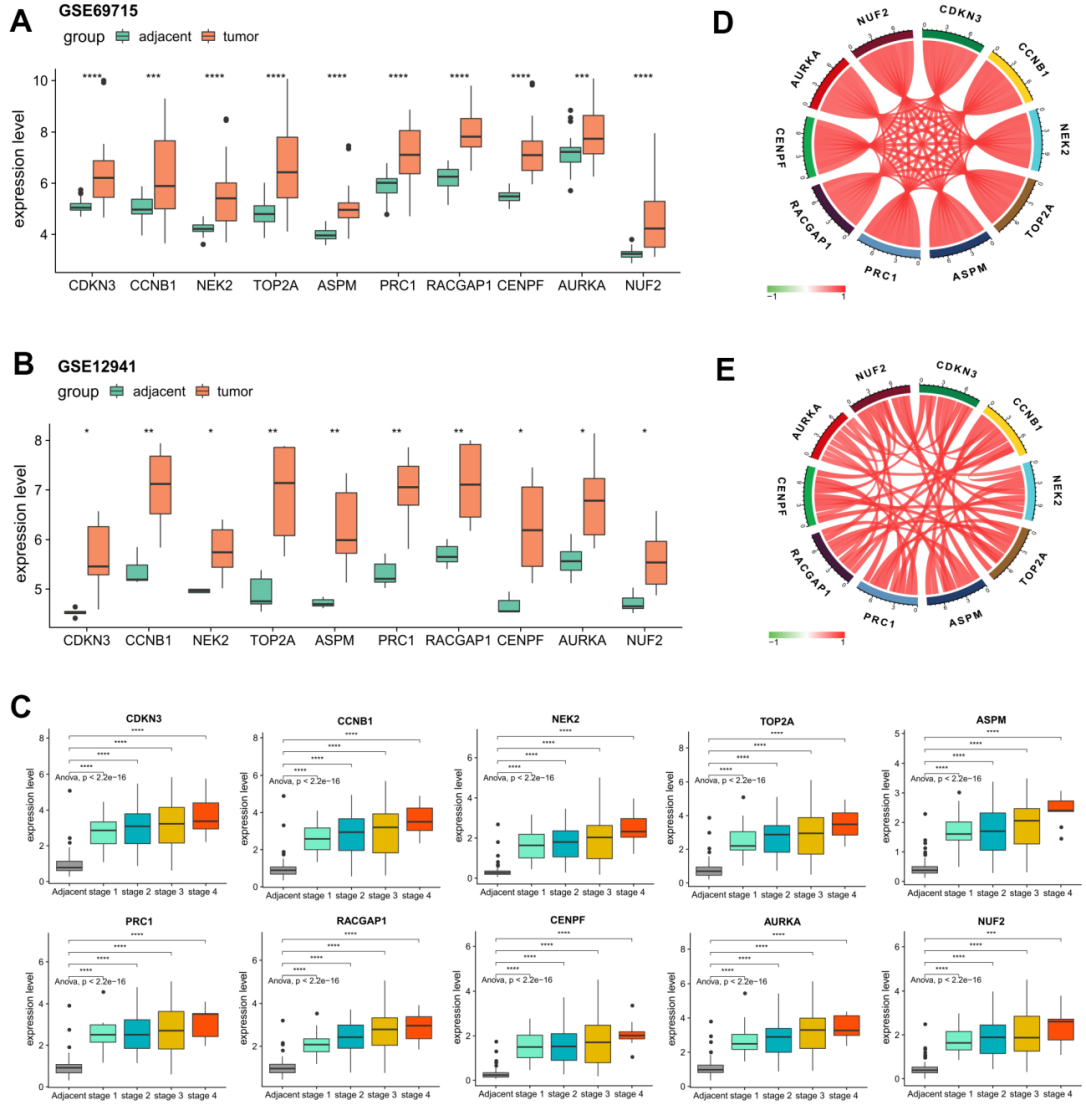
**Confirmation of the abnormal expression of the 10 selected hub genes and their expression correlations.** (**A**, **B**) Two external datasets (GSE69715 and GSE12941) to validate the increased expression levels of the hub genes in tumors compared with adjacent normal tissues. (**C**) Internal validation by ICGC-LIRI-JP dataset to verify the elevated levels of the hub genes concerning tumor stage. (**D**, **E**) Strong correlations among all of the hub genes according to the ICGC-LIRI-JP and TCGA-LIHC datasets. *P < 0.05, **P < 0.01, ***P < 0.001, ****P < 0.0001.

For the validation of the expression patterns, based on the external validation datasets of GSE69715 and GSE12941, we observed significantly elevated gene expression levels of every hub genes in tumor samples compared with that of the adjacent normal samples (Figure 6A, 6B). A closer examination of the internal validation set of ICGC-LIRI-JP showed that the dysregulations of all the hub genes were statistically significant regardless of the tumor stage (Figure 6C), indicating the robustness of their crucial roles in tumor initiation of HCV-HCC. Strikingly, it was determined that in both ICGC-LIRI-JP and TCGA datasets, the relative expression levels of the hub genes were highly correlated with each other (Pearson correlation coefficient > 0.75 for all gene pairs in both ICGC-LIRI-JP and TCGA-LIHC), suggesting their strong interactions and key roles in the development of HCV-HCC (Figure 6D, 6E).

### Validation of the diagnostic value

**Figure 7:**
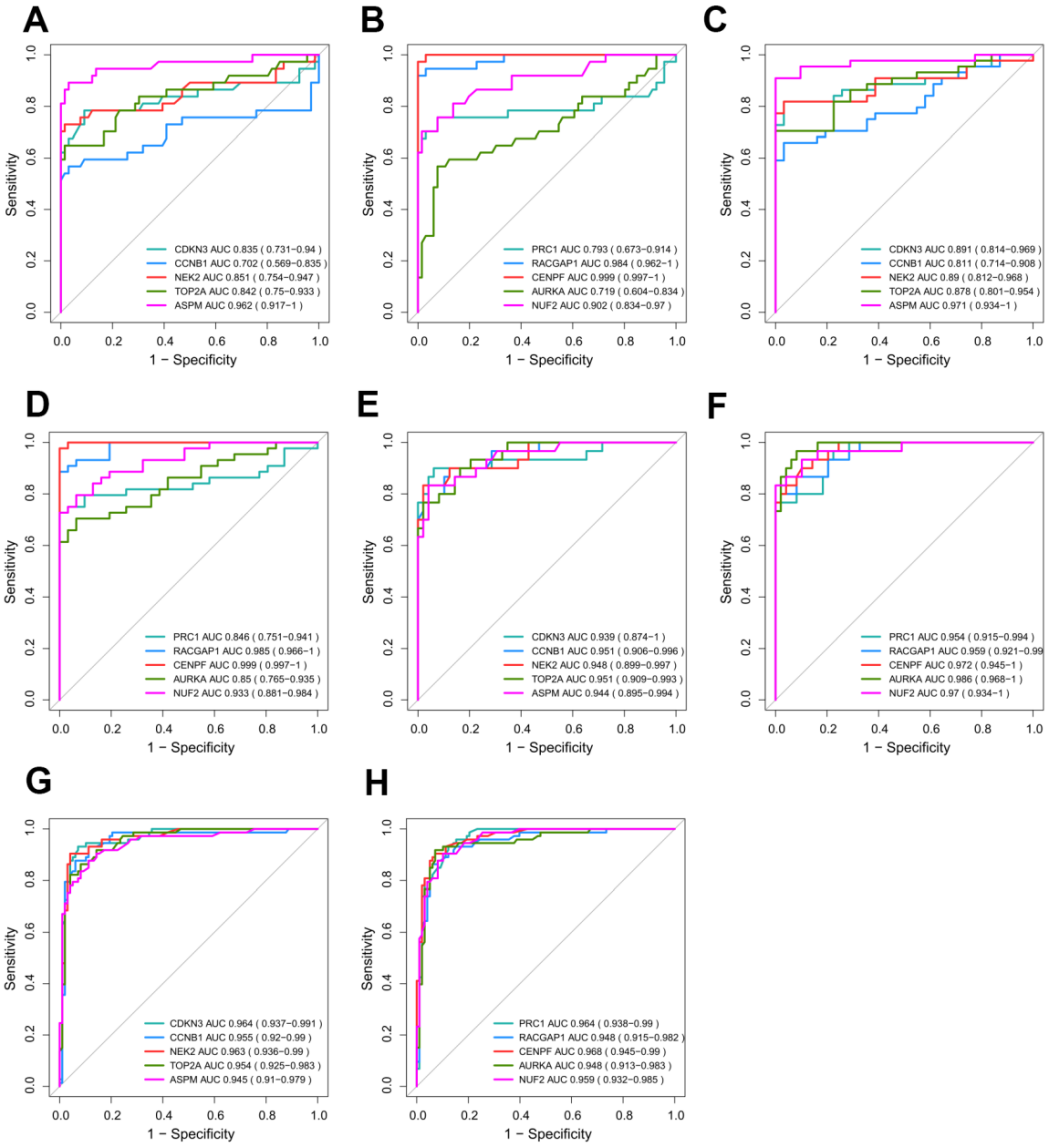
**Validation of the diagnostic efficiency for each of the 10 hub genes.** (**A**–**F**) Performance of the 10 hub genes in discriminating HCV-HCC from normal control based on GSE69715 (**A**, **B**), GSE107170 (**C**, **D**), and TCGA-LIHC (**E**, **F**). (**G**, **H**) Potential utilities of the hub genes for early tumor detection based on ICGC-LIRI-JP. HCV-HCC, HCV- associated HCC.

We presume that excellent discrimination capability may have great potential for cancer diagnosis to benefit HCV-HCC patients. Thus, we validated the performance of hub genes by plotting ROC curves of GSE69715, GSE107170, and TCGA-LIHC (Figure 7A–7F). Two hub genes (CENPF and RACGAP1) showed consistently high AUROC scores in all three datasets (>0.95), indicating their penitential utility as diagnostic biomarkers. Moreover, we used the internal validation set of ICGC-LIRI-JP to assess the distinguishing abilities of the hub genes for early phase tumor samples from adjacent normal tissue samples (Figure 7G, 7H). Surprisingly, ROC curves by all the hub genes revealed their great potential for early detection of HCV-HCC (AUROC score > 0.94 for each hub gene).

### Survival analysis

**Figure 8:**
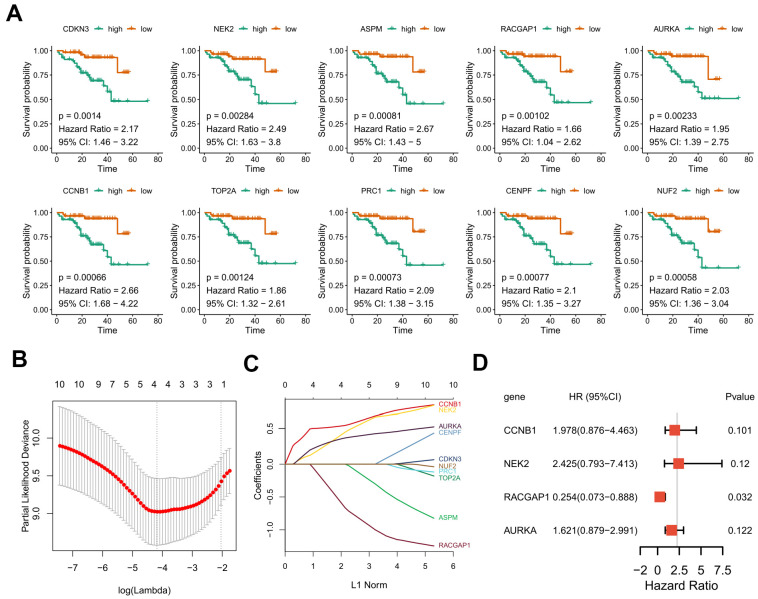
**Kaplan–Meier curves for overall survival of the 10 selected hub genes and construction of a prognostic signature using LASSO Cox regression.** (**A**) OS Kaplan–Meier curves of the 10 hub genes based on ICGC-LIRI-JP. (**B**) 10-fold cross-validation to select the optimal tuning parameter. The λ value of 0.015 was chosen with the lambda.min method. (**C**) LASSO coefficient profiles of the 10 hub genes. (**D**) Forest plot presenting the hazard ratio and 95% CI by multivariate Cox regression analysis for the four selected hub genes. OS, overall survival. LASSO, Least absolute shrinkage and selection operator. 95% CI, 95% confidence interval.

**Figure 9:**
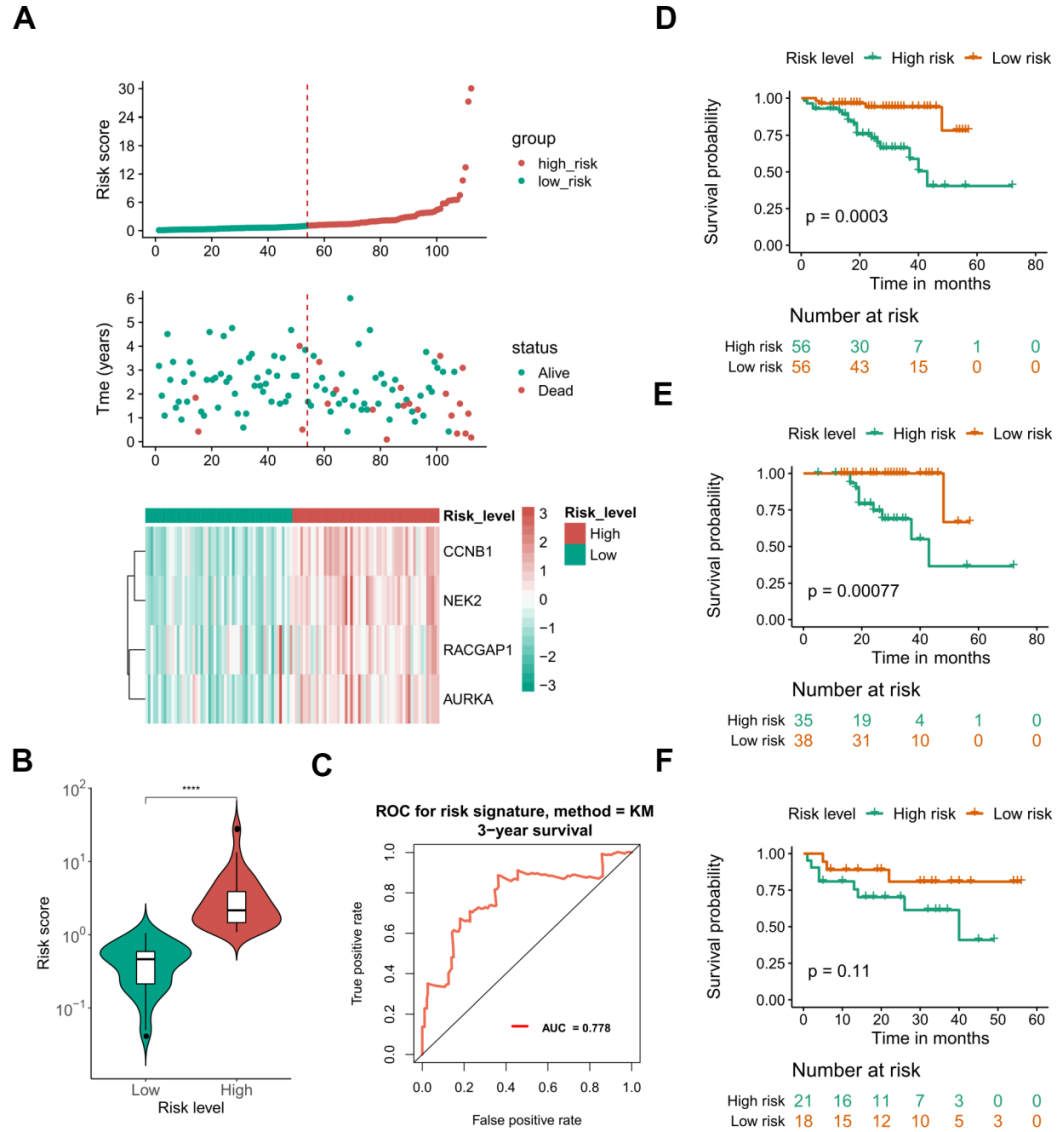
**Performance of the defined four mRNA-based risk signature with ICGC-LIRI-JP.** (**A**) Gene expression, risk score, and clinical outcome for all the patients in distinctive risk groups. (**B**) differential risk scores between high- and low-risk groups. (**C**) ROC plot at 3 years OS showing the AUROC score of 0.778. (**D**) OS Kaplan-Meier survival curves for high- and low-risk patients. (**E**, **F**) OS Kaplan-Meier survival curves for different risk groups of early stage (**E**) and advanced stage patients (**F**). ****, *P* < 0.0001. OS, overall survival. ROC, receiver operating characteristic. AUROC, the area under the receiver operating characteristic curve.

Due to the limited sample sizes of other datasets, we were only able to include the ICGC-LIRI-JP cohort that contained more than 100 HCV-HCC patients with adequate survival information to conduct the survival analysis (N = 112). Kaplan–Meier curves indicated that the overall survival of the high-risk group was significantly lower than that of the low-risk group (*P* < 0.01 for all hub genes, Figure 8A). Furthermore, the LASSO-COX regression was used to reduce the variables with 10-fold cross-validation for the selection of the optimal turning parameter (Figure 8B). At the minimum lambda value, four hub genes were chosen with non-zero coefficients, including CCNB1, NEK2, RACGAP1, and AURKA (Figure 8C), which were next used to perform the multivariate Cox hazards regression analysis (Figure 8D). A risk signature was then generated to evaluate the risk score of HCV-HCC patients with the following formula: risk score = 0.6819* EXP_CCNB1_ + 0.8859*EXP_NEK2_ -1.3715*EXP_RGCGAP1_ + 0.4831*EXP_AURKA_. Patients were divided into the high- or low-risk groups according to the median risk score of 0.8822715 (Figure 9A). A significantly higher risk score was observed in the high-risk group than that of the low-risk group (Figure 9B). The ROC curve at 3 years overall survival showed the area under the curve (AUC) value of 0.778 (Figure 9C), indicating a good predictive performance for the OS of HCV-HCC. Kaplan-Meier survival plots suggested the relatively poor survival in the high-risk group (Figure 9D). Besides, we carried out the stratified analysis using clinical parameters. Consequently, in almost all subsets of patients with different age, gender, vein invasion status, alcohol consumption, and smoking status, the four-hub gene-based risk signature was still a significant prognostic factor (Supplementary Figure 2). Although the TNM staging system was considered as an important prognostic factor for HCC patients, conflict survival outcomes may exist for patients at the same stage. Therefore, we also performed the stratification survival analysis based on the TNM stage. Notably, patients in the low-risk group possessed a better OS compared with the high-risk group in the early stage subset (N = 73, *P* < 0.01) (Figure 9F), while no significant difference was observed for the advanced stage of HCV-HCC (N = 39, *P* = 0.11) (Figure 9F). Besides, we also conducted the univariate Cox analysis to evaluate the other underlying risk factors, however, no significant associations were observed at a statistical level of 0.05, which might partly due to the small sample size.

### The risk signature was associated with the abundance of immune infiltration cells

**Figure 10:**
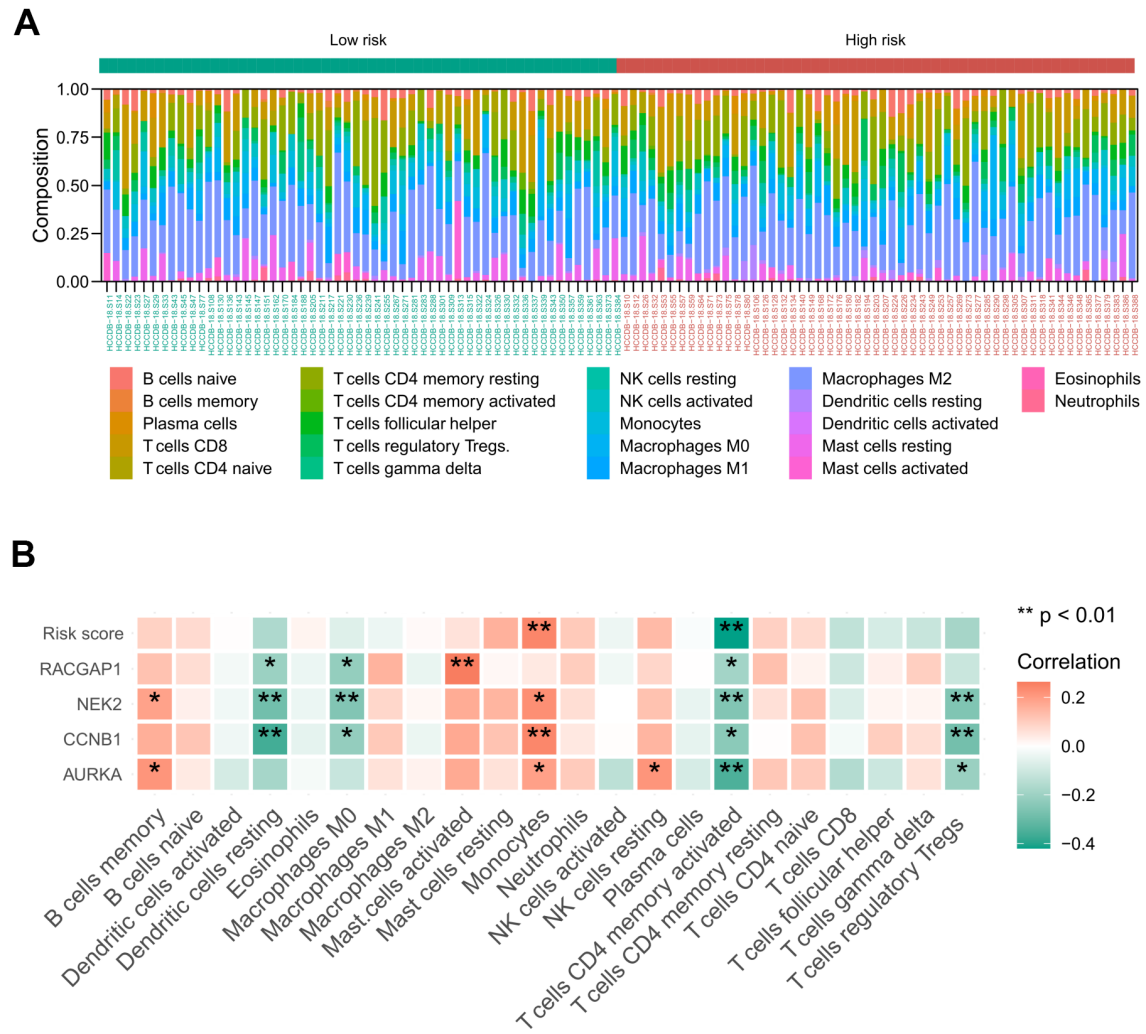
**Relationship between the identified risk signature and tumor immune cell infiltration based on the ICGC-LIRI-JP cohort.** (**A**) The landscape of immune infiltration in each of the tumor samples of low- and high-risk groups. (**B**) Heatmap representing the correlation matrix of the four signature genes, risk score, and relative abundance of 22 immune cell types. Red indicates the positive correlation, while green indicates the negative correlation. * *P* < 0.05, ** *P* < 0.01.

Based on the ICGC-LIRI-JP cohort, we achieved the landscape of the 22 tumor immune infiltration cells for HCV-HCC via the CIBERSORT algorithm (Figure 10A). Then the Spearman correlation coefficient and corresponding *P* value between risk score and infiltration level of each immune cell were calculated. As a result, monocytes were positively associated with the risk score and the expression of NEK2, CCNB1, and AURKA. Activated CD4 memory T cells displayed negative correlations with the risk score and all of the four signature hub genes. Other immune cells manifested no significant correlation with the risk score, except resting dendritic cells and M0 macrophages, which were negatively associated with the expression of RACGAP2, NEK2, and CCNB1. T cells regulatory Tregs were negatively associated with the expression of NEK2, CCNB1, and AURKA (Figure 10B).

### Prediction of upstream regulations

**Figure 11:**
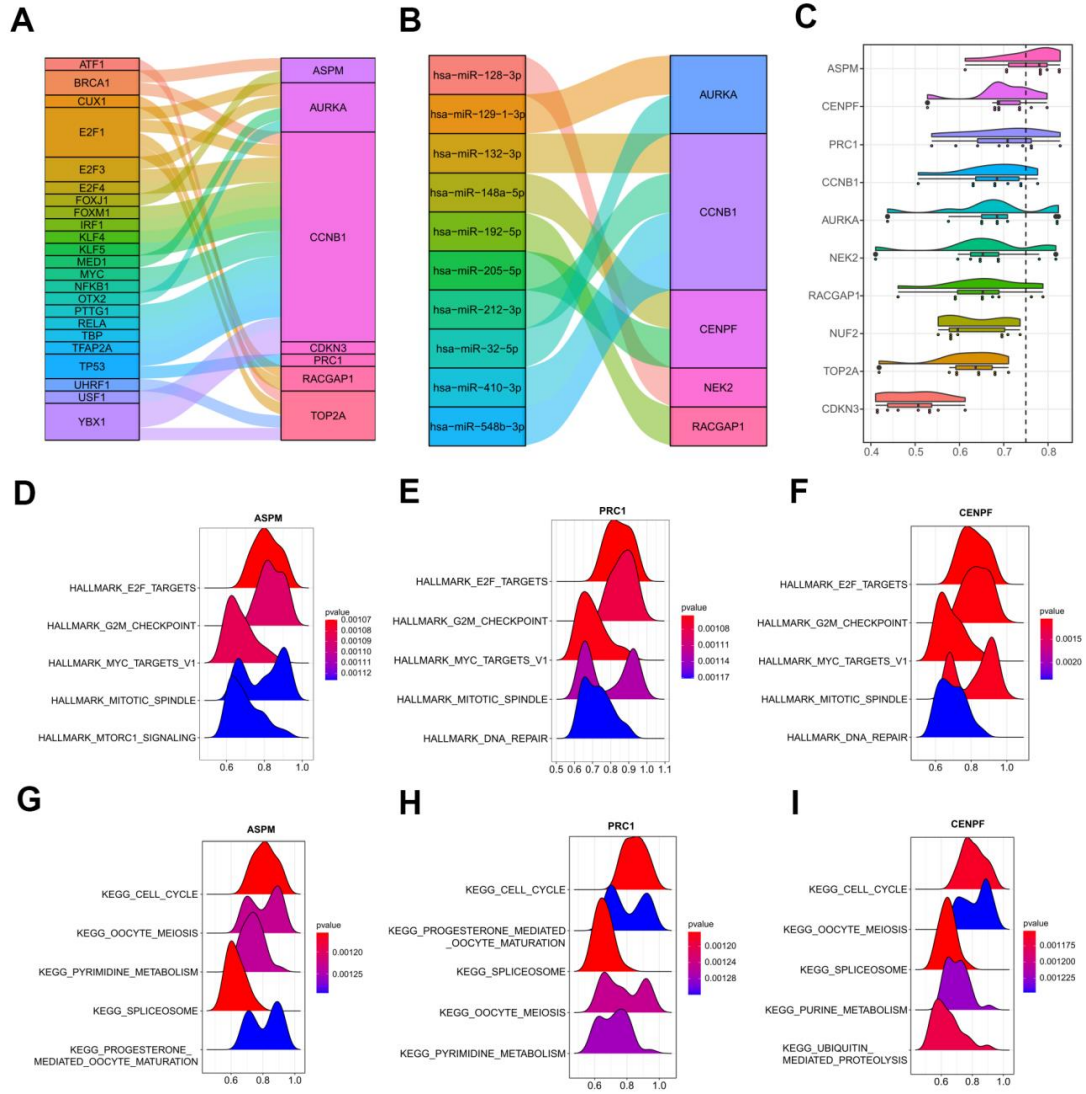
**Upstream regulations of the ten hub genes and GO semantic similarities analysis.** (**A**) The transcription factor-hub gene network predicted by miRNet. (**B**) 10 function MTIs predicted through miRTarBase 8.0. (**C**) Raincloud plot showing the ranking list of function semantic similarities for the 10 hub genes using the ICGC-LIRI-JP dataset. ASPM, CENPF, and PRC1 were the top three hub genes with the highest scores. (**D**–**F**) GSEA results of ASPM, CENPF, and PRC1 based on the hallmark gene set. (**G**–**I**) GSEA results of ASPM, CENPF, and PRC1 based on the KEGG database. GO, gene ontology. MTIs, miRNA-target interactions. KEGG, Kyoto Encyclopedia of Genes and Genomes.

Next, crucial transcription factors in the upstream of the 10 hub genes were determined by the TRRUST database that was integrated into the web-based application of miRNet (Supplementary Table 4). A transcription factor-hub gene network was then constructed and visualized by a Sankey diagram. 23 transcription factors and 7 hub genes were found in this network (Figure 11A). Among all the genes, CCNB1 was the most important node with the highest degree, which was regulated by a total of 19 transcription factors, including several important tumor-associated genes, such as BRCA1, MYC, TP53, and three E2Fs family members (E2F1, E2F3, and E2F4). Moreover, miRNA-hub gene interactions were predicted by employing miRTarBase 8.0 to explore the underlying regulatory mechanism by miRNAs in human. A total of 428 miRNA-hub gene interaction pairs were obtained, including 10 function MTIs, 417 weak function MTIs, and one none function MTI (Supplementary Table 5). As illustrated in Figure 11B, among the 10 function MTIs, CCNB1 gave the highest connection degree, which was targeted by four miRNAs (hsa-miR-132-3p, hsa-miR-212-3p, hsa-miR-548b-3p, hsa-miR-410-3p) in experiment validation, suggesting its increasing correlation with human cancer. Other predictive weak function MTIs were still needed to be investigated in the future. Hence, the transcription factor-hub gene interaction network and the identified miRNA-hub gene pairs could provide insight into the further exploration of the molecular mechanisms of HCV-HCC.

### Semantic similarities and GSEA

In order to achieve a deeper and better understanding of the molecular mechanism of the 10 hub genes, we adopted the geometric mean of CCs and MFs with GO semantic similarity analysis. ASPM, CENPF, and PRC1 were the top three hub genes with the highest-ranking scores (Figure 11C), which were verified by GSEA analysis with the ICGC-LIRI-JP dataset. The top 5000 genes that correlated with the ASPM, CENPF, and PRC1 were used to operate the GSEA, and the most significant terms that enriched by the hallmark gene set (h.all.v7.0) were similar (HALLMARK_E2F_TARGETS, HALLMARK_G2M_CHECKPOINT, HALLMARK_MYC_TARGETS_V1, and HALLMARK_MITOTIC_SPINDLE were the top four gene sets for all of the three hub genes) (Figure 11D–11F). For the pathways distribution from the KEGG database (c2.cp.kegg.v7.0), ASPM, CENPF, and PRC1 shared three important tumor-associated terms (KEGG_CELL_CYCLE, KEGG_OOCYTE_MEIOSIS, and KEGG_SPLICEOSOME) in the top five significant pathways (Figure 11G–11I). These findings were in agreement with the abovementioned results that hub genes may strongly interact with each other and strengthened the pivotal roles of the hub genes in the tumor initiation and development in HCV-HCC.

### Drug-hub gene network and candidate compounds identification

**Figure 12:**
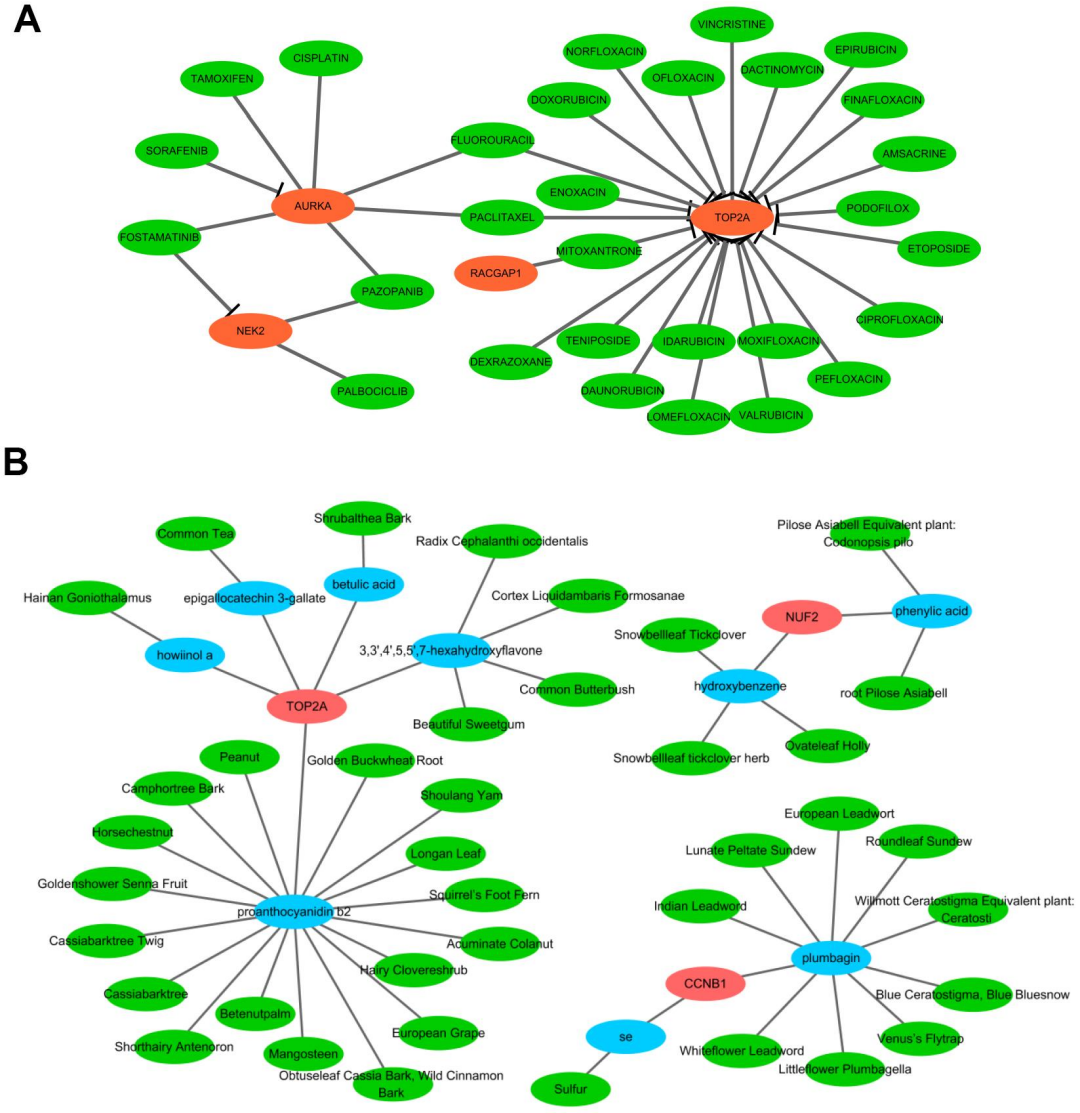
**Network pharmacological analysis to identify candidate drugs and effective compounds for therapeutic targets of HCV-HCC.** (**A**) Drug-hub gene network identified from the DGIdb. Green nodes indicate the predictive miRNAs and red nodes indicate the targeted hub genes. (**B**) Herb-compounds-hub gene network predicted by TCM-MESH and TCM-ID. red nodes indicate hub genes, blue nodes indicate the active compounds and green nodes indicate the putative herbs containing these compounds.

The aforementioned results prompted us to focus on the therapeutic utility of the hub genes. Thus, we searched the DGIdb to obtain the potential drug-gene interactions. As a result, four (TOP2A, AURKA, NEK2, and RACGAP1) of the 10 hub genes were targeted by therapeutic drugs that were approved by FDA. Then a drug-hub gene network was established including the four hub genes and 29 anti-neoplastic drugs (Figure 12A and Supplementary Table 6). As presented in Figure 12A, TOP2A might be inhibited by most of the drugs, followed by AURKA. Among these drugs, Etoposide was supported by the largest number of literature as the cancer chemotherapeutic drug including HCC [13]. Additionally, TCM-MESH and TCM-ID were used to investigate candidate active ingredients and herbs that may target these hub genes. A network consisting of 3 hub genes (TOP2A, CCNB1, and NUF2), 9 effective compounds, and 40 related herbs was constructed (Figure 12B). Similarly, TOP2A was putatively targeted by most compounds (3,3',4',5,5',7-hexahydroxyflavone, proanthocyanidin b2, epigallocatechin 3-gallate, howiinol a, and betulic acid). Among all the compounds, proanthocyanidin b2 and plumbagin showed the top two nodes with the highest degrees (proanthocyanidin b2 was contained in 17 herbs and plumbagin was contained in 9 herbs). Proanthocyanidin b2 was well-documented with anticarcinogenic properties via anti-inflammator and antioxidant potential, and was demonstrated to exert anti-tumor efficacy for HCC *in vitro* and *in vivo* [14]. Plumbagin was also indicated to suppress HCC carcinogenesis through induction of cell arrest and cellular apoptosis [15]. These data may shed light upon target therapy for HCV-HCC patients.

### Comparison of the hub genes and pathways between HCV-HCC and HBV-HCC

In a previous study, we reported 17 hub genes with diagnostic and prognostic values in HBV-HCC [16]. Interestingly, three of them (CCNB1, TOP2A, NEK2) were also identified as crucial genes in HCV-HCC, which might to some extent reflect the common transcriptome regulatory mechanisms in liver cancer induced by viral hepatitis. We also compared the robust DEGs between HCV-HCC and HBV-HCC, as the result, we found 38 common upregulated DEGs and 95 common downregulated DEGs. Notably, commonly important KEGG pathways enriched by robust DEGs were identified between HCV-HCC and HBV-HCC including cell cycle, p53 signaling pathway, oocyte meiosis, progesterone-mediated oocyte maturation, Human T-cell leukemia virus 1 infection, cellular senescence, retinol metabolism, tryptophan metabolism, complement and coagulation cascades, drug metabolism - cytochrome P450, tyrosine metabolism (Supplementary Figure 3). Knowledge like that may reveal indispensable and key pathways for the complete transition from hepatitis to HCC, and therefore would throw light on the yielding of possible predictors or biomarkers during the process.

## DISCUSSION

Despite intense efforts that have been made for the investigation of HCC pathogenesis and its candidate biomarker searching, the overall prognosis for HCC patients was still unfavorable, and the comprehensive explanation for its transcriptional and genetic mechanisms remained elusive, especially for HCV associated HCC. In the current study, 240 robust DEGs of HCV-HCC were, for the first time, screened based on five public datasets, including 58 upregulated genes and 182 downregulated genes. The upregulated genes mainly participated in cell cycle-associated GO terms, such as cell division, cell cycle phase transition, and spindle. Cell-cycle aberration was considered a hallmark of cancer [17]. In the present study, those cell cycle-related genes such as CDK1, CCNB1, CDC20, NEK2, AURKA, RACGAP1, CDKN2A, CDKN2B, CDKN3, RRM2, and ASPM were significantly upregulated in HCV-HCC. downregulated genes were mostly involved in the monocarboxylic acid metabolic process, cellular response to cadmium ion, and oxidoreductase activity. KEGG analysis revealed that, while the upregulated genes were significantly related to cell cycle, p53 signaling pathway, and oocyte meiosis, the downregulated genes were associated with tryptophan metabolism, retinol metabolism, and mineral absorption. The common pathways identified via a deeper examination between HCV-HCC and HBV-HCC may help reveal the generality of the transition from viral hepatitis to HCC.

Subsequently, 10 hub genes were identified through multiple approaches by overlapping a panel of 30 closely correlated DEGs-PPI-hub genes and 50 WGCNA-PPI-hub genes, as well as assessing their diagnostic and prognostic power. Interestingly, in comparison with the adjacent normal tissues, all of the hub genes were overexpressed in the tumor tissue samples, suggesting their tumor-driven function in oncogenesis. Next, the consistent and significant expression trend of the hub genes was validated internally and externally. Notably, the expression levels of all the hub genes were associated with each other, indicating their tight connections and pivotal roles during HCV-HCC. Moreover, ROC curves revealed that CENPF and RACGAP1 exhibited robust discrimination between tumor and normal tissue samples. More importantly, all of the hub genes displayed great potential for early diagnosis of HCV-HCC according to the ICGC-LIRI-JP dataset.

To improve the reliability of their prognostic values, we depicted the Kaplan–Meier OS survival plot for each of the hub genes, combined with a log-rank test. As a result, all the hub genes showed a negative impact on patients’ OS. Furthermore, we generated a four-hub gene-based risk signature (CCNB1, NEK2, RACGAP1, and AURKA) via LASSO-COX proportional regression analysis, which was demonstrated to have excellent prognostic accuracy for OS of HCV-HCC patients. It was worth noting that even though the TNM staging system was most frequently used to predict the outcomes of HCC, our model could still offer additional value for subgroups with different TNM stages and other clinicopathological variables.

In previous studies, all of the four risk signature genes had been reported to play oncogenic roles in HCC and their elevated expression levels were closely related to the reduced overall survival of HCC patients [18–27], which is in agreement with this study. For example, CCNB1 and AURKA were proved to be significantly associated with the prognosis of HCC and HBV-HCC and proposed as hub genes in these cancers [16, 19, 25–28]. Weng, L. et al. demonstrated that the elevated expression of CCNB1 was an independent prognostic indicator for the recurrence in HBV-HCC patients [27]. Recently, CCNB1 was used to build an mRNA risk signature to predict the prognosis of HCC [28], and AURKA was also involved in a 24 mRNA-based signature for early relapse prediction of HCC [6]. NEK2 was showed to promote tumor growth, angiogenesis, and metastasis *in vivo*. Its overexpression was found in liver tumor tissues on both mRNA and proteomic levels, and was associated with the poor survival of HCC [20]. Another study revealed RACGAP1 as a prognostic factor for early recurrence of HCC. Silencing of RACGAP1 could significantly inhibit Hep3B and MHCC97-H cell invasion and migration [22]. Other hub genes were also reported to be predictors for HCC prognosis. It is notable that TOP2A was a widely accepted hub gene in both HCC and HBV-HCC [16, 19, 28, 29], and it was also used to establish an mRNA-based signature for prognosis prediction in HBV-HCC previously [16]. Moreover, CENPF was correlated with higher cumulative recurrence rates and shorter overall survival of HCC [30, 31]. Besides, high expression levels of NUF2 [32], CDKN3 [33], ASPM [33], PRC1 [34] were also related to poor prognosis of HCC. What’s important, CDKN3 was linked to the activated or inhibited cell cycle modules for the transformation of non-malignancy-associated hepatitis/cirrhosis to HCC.

Given that dynamic crosstalk among tumor immune infiltration cells in the tumor microenvironment may trigger and accelerate tumor growth or progression [35], we put emphasis on the correlations between risk score and relative abundance of immune cells. Eight putative immune cell types were found to have significant correlations with risk score or signature hub genes.

To gain insights into the upstream regulatory mechanisms of these hub genes, a transcription factor-hub gene network and 10 function MTIs were identified. Remarkably, most of the transcription factors were well-established oncogenes or tumor suppressor genes. Among the 10 experimentally verified miRNAs, most of them, including miR-128-3p [36], miR-132-3p [37], miR-148a-5p [38], miR-192-5p [39], miR-205-5p [40], miR-212-3p [41], miR-32-5p [42] were previously linked to tumor cell proliferation, invasion, migration and prognosis of HCC. Meanwhile, further researches are still required to elucidate the biological functions of the remaining three miRNAs (miR-129-1-3p, miR-410-3p, and miR-548b-3p) on tumorigenesis and progression of HCC. Moreover, GO semantic similarity analysis and GSEA suggested that ASPM, CENPF, and PRC1 may share common molecular mechanisms during the pathogenesis of HCV-HCC.

For the evaluation of therapeutic implications of the hub genes, we carried out network pharmacological analysis. We found that four of the hub genes (TOP2A, AURKA, NEK2, and RACGAP1) can serve as tumor therapeutic targets for drugs approved by FDA. Specifically, a set of TOP2A inhibitors were determined as potential chemoprotective drugs in various types of cancer, such as doxorubicin in solid tumors, leukemias and lymphomas [43], Idarubicin in HCC [44], acute myelogenous leukemia, advanced breast cancer, multiple myelom, non-Hodgkin's lymphoma, and other malignancies [45], and etoposide in several malignant tumors [46–50] and metastatic tumors (such as brain metastasis of breast cancer) [51, 52]. Next, we identified candidate herbs and their effective components that may have an inhibitory impact on tumor progression via three hub genes (TOP2A, NUF2, and CCNB2). Proanthocyanidin b2 and plumbagin were the most common compounds in herbs related to TOP2A and CCNB1, showing good potential for cancer treatment including HCC [14, 15].

Compared with previous studies, the present study has at least several strengths: first, most studies only enrolled one cohort or single method to screen DEGs in cancer, while a total of eight high-quality gene expression profile datasets with stringent approaches (combining the overlapping strategy and the integrating strategy) improved the robustness. Second, we performed four approaches to identify potential key genes in HCV-HCC, which was different from those derived from only one algorithm (such as PPI network or WGCNA). Third, unlike previous studies that neglected population stratification while constructing a gene signature, we focused on a specific cohort of HCC that was influenced by HCV. Furthermore, the comparison between HCV-HCC and HBV-HCC may help understand the generality and specificity of the transformation from hepatitis B or hepatitis C to HCC. Additionally, the hub gene-based drugs or effective compounds may provide new insight for targeted therapy in HCV-HCC.

Several limitations, however, should be addressed in this study. First, due to the strict patient inclusion criteria applied in this study, only one available cohort (ICGC-LIRI-JP) was included for survival analysis, which may introduce imprecision or potential bias in the evaluation of risk factors, and increase the risk of overfitting during the construction of the prognostic gene signature. Therefore, more external validation cohorts with larger sample sizes are required to validate our prognostic signature and their relevance to immune cell infiltration. Second, more *in vitro* and *in vivo* experiments should be performed to uncover the molecular mechanisms of the predicted transcription factor-hub gene pairs and putative miRNAs that may target the hub genes during HCC tumorigenesis and cancer progression. Third, it should be noted that the candidate drugs and potential active components targeting the hub genes should be further investigated, from structural analysis (such as molecular docking) to in-depth experimental studies for functional exploration, which may help accelerate the development of novel promising drugs for target therapy of HCC.

In summary, we identified 10 hub genes, which may play crucial roles in the carcinogenesis and pathogenesis of HCV-HCC, from multiple datasets with comprehensive bioinformatics approaches. The dysregulation of the hub genes was linked to tumor diagnosis and prognosis and might serve as potential therapeutic targets of HCV-HCC patients. A risk signature was constructed for OS survival classification. A transcription factor-hub gene network and a series of targeted miRNAs were predicted. Potential drugs and candidate compounds for these hub genes were identified. All these results from the multi-dimension analysis provide a strong foundation for a better understanding of the complex transcriptional regulatory mechanisms underlying HCV-HCC, which might shed light on the discovery of potential biomarkers for early diagnosis, prognosis, and treatment for HCV-HCC patients.

## MATERIALS AND METHODS

### Data acquisition

Six gene expression profiles of HCC were selected from the GEO (https://www.ncbi.nlm.nih.gov/geo/) database with the GSE number of GSE6764 [53], GSE41804 [54], GSE62232 [55], GSE107170 [56], GSE12941 [57], and GSE69715 [58]. These datasets met the following strict criteria: (1) including both tumor and normal human tissues; (2) with information of HCV infection; (3) containing at least six HCC-HCV samples. HCV-HCC cases were carefully examined and picked out. Five datasets (GSE6764, GSE41804, GSE62232, GSE107170, GSE69715) were based on GPL570 (Affymetrix Human Genome U133 Plus 2.0 Array) and GSE12941 was based on GPL5175 (Affymetrix Human Exon 1.0 ST Array). We also collected the pretreated data of HCV-HCC samples and the corresponding clinical information of TCGA-LIHC (http://www.tcga.org/) and ICGC-LIRI-JP (https://icgc.org/) from the HCCD database [59]. Five of them (GSE6764, GSE41804, GSE62232, GSE107170, and TCGA-LIHC) were served to screen DEGs, and the remaining three sets were used for further analysis. All of the above studies comprised a total of 304 HCV-HCC and 290 adjacent normal, and detailed information was summarized in Supplementary Table 1.

### Screening of differentially expressed genes (DEGs)

Differential analysis for each of the above-mentioned microarray datasets was performed by GEO2R (https://www.ncbi.nlm.nih.gov/geo/geo2r/) with default settings. For the TCGA-LIHC dataset, the level three normalized mRNA expression profile was downloaded from the HCCD database, and the limma package [60] was adopted to pick out DEGs between HCV-HCC and normal samples. Statistical significance was set as |log Fold change (FC)| > 1 and FDR (adj.P.Val) <0.05. Thereafter, the intersected DEGs were obtained and visualized by the UpSetR [61] and VennDiagram [62] packages. In order to further validate the robustness of the DEGs, we conducted the integrated analysis and differential analysis of the four microarray datasets with the aid of sva and limma packages [63].

### Weight Gene Co-expression Network Analysis (WGCNA) and module identification

The WGCNA network was constructed by the WGCNA package [64] based on the gene expression data of ICGC-LIRI-JP. In the beginning, the DEGs from ICGC-LIRI-JP dataset were screened by limma package at the cutoff of |log Fold change (FC)| > 1 and FDR <0.05, which were used to detect and eliminate outlier samples through the sample clustering tree. Next, an appropriate soft threshold was used to obtain scale-free networks. Then topological overlap matrix (TOM) and the dissimilarity (dissTOM) were computed and used to implement the gene dendrogram and module recognition (minClusterSize = 30). Similar modules were merged into larger ones at a cutline of 0.3. To determine their relevance to clinical traits, Pearson correlations between module eigengenes and clinical phenotypes including age, gender, TNM stage, alcohol consumption, smoking status, survival time, and survival status were calculated and shown with a correlation heatmap. In this study, we chose the most significant module that correlated with survival status for further analysis, and gene significance (GS) and module membership (MM) were also calculated.

### Protein-protein interaction (PPI) network construction

PPI network is a useful approach to explore molecular interactions related to tumorigenesis and progression. In this study, a PPI network comprising the overlapping DEGs was constructed by the Search Tool for the Retrieval of Interacting Genes (STRING) database (version 11.0; http://string-db.org/). A comprehensive interaction score of ≥ 0.7 was set as the threshold (high confidence). Visualization of the PPI network was done by Cytoscape (version 3.2.1; http://www.cytoscape.org) [65]. The MCODE plugin of Cytoscape was used to obtain the most significant cluster in the network. Topological parameters were calculated by cytohuber app [66] and we chose the top 30 nodes that had a degree of > 20 as DEGs-PPI hub genes. Besides, to fetch the hub genes in the significant module that correlated with survival status, we also uploaded the corresponding genes in the selected module to the STRING database to establish the WGCNA-PPI network, which was used to identify WGCNA hub genes according to the node degree threshold (≥50).

### Hub genes identification

In addition to their putative pivotal role in fostering tumorigenesis of cancer, we envisaged that hub genes would provide diagnostic and prognostic values in HCV-HCC patients. So, we picked out the overlapping genes in the PPI hub genes and the WGCNA hub genes and assessed their predictive capabilities for diagnosis and prognosis based on the expression profile of the ICGC-LIRI-JP dataset. For the assessment of their diagnostic powers, we depicted the ROC curves of the overlapping genes by the pROC package [67] to rank their area under the receiver operating characteristic curve (AUROC) scores from high to low, and an AUROC score of > 0.95 was used set as the criterion for selection. To evaluate their prognostic values, only 112 HCV-HCC patients with complete clinicopathologic characteristics (age, gender, TNM stage, vein invasion, alcohol consumption, and smoking status) and available follow-up information (overall survival outcome) were included. The prognostic powers of overlapping genes were estimated by univariate Cox regression (UniCox) with a *P*-value threshold of less than 0.05. A forest plot was drawn to present the hazard ratio (HR) and *P*-value obtained from UniCox analysis. Only genes that satisfied all these conditions were regarded as hub genes in this study.

### Function enrichment

Metascape database [68] was used to perform the gene ontology (GO) analysis of the upregulated genes, the downregulated genes and of the most significant module in the WGCNA network. Significant terms were defined with a *P* < 0.01 and count > 3. For the Kyoto Encyclopedia of Genes and Genomes (KEGG) pathway analysis, the “clusterProfiler” package [69] was utilized and FDR < 0.05 was set as a cutoff.

### Validation of the hub genes’ dysregulation patterns

Three gene expression datasets including ICGC-LIRI-JP, GSE69715, and GSE12941 were used for the validation of the expression patterns of the identified hub genes. We firstly used GSE69715 and GSE12941 as the external datasets to compare the expression levels of the hub genes in tumor vs normal by *t*-test, followed by the investigation of the comparison of that according to different TNM stages, which was conducted through the internal validation set of ICGC-LIRI-JP. Moreover, Pearson correlations of the hub genes’ expression values were also carried out with ICGC-LIRI-JP and TCGA datasets.

### Validation of the hub genes’ diagnostic abilities

For the evaluation of the hub genes’ diagnostic efficiencies, we depicted the ROC curves of GSE69715, GSE107170, and TCGA-LIHC with the pROC package, using the corresponding gene expression profiles. To explore their performance in differentiating the early phase of HCV-HCC from normal liver tissues for early detection possibilities, we used the ICGC-LIRI-JP dataset to generate ROC curves to quantify their predictive powers to discriminate between early stage tumor samples (N =73) and normal control (N =98).

### Survival analysis and construction of the risk signature

To further validate the prognostic values of the identified hub genes, Kaplan–Meier overall survival (OS) curves and log-rank tests were carried out based on the gene expression profile of the ICGC-LIRI-JP dataset. Patients were assigned to different risk groups according to the median expression value, then the survival package [70] was used to measure HR and clinical significance. To construct a prognostic risk signature, we conducted LASSO-COX analysis with the glmnet package [71] to identify the most important hub genes for OS of HCV-HCC. Genes retained by the LASSO regression algorithm were used to build a linear combination to generate the risk score for each patient with the following formula: risk score = Σ (coef (hub gene) × expression value of hub gene). Patients were separated into high- or low-risk groups according to the median risk score, and survival difference between the two groups were compared by Kaplan–Meier curves. The predictive power of the risk signature was investigated by the ROC curve at 3 years, which was accomplished with the survivalROC package [72]. Besides, Stratified analysis was also performed for clinicopathological features including TNM stage, age, gender, vein invasion status, alcohol consumption, and smoking status. For all statistical tests, *P*<0.05 was set as the significant cutoff.

### Correlations between immune response and the risk signature

To explore the relationship between our risk signature and immune response, we utilized the CIBERSORT algorithm [73] to obtain the estimation of the percentage for 22 immune cell types in each of the HCV-HCC patients based on the ICGC-LIRI-JP cohort. The relative abundance of immune cells in high- and low-risk groups was computed and presented by a heatmap plot. Spearman correlation analysis was applied to determine the relevance of risk score and immune cell infiltration. Besides, the correlation between each of the risk signature genes and the immune cell was also investigated and visualized by a correlation heatmap.

### Prediction of upstream regulators for the hub genes

The upstream transcription factors of hub genes were explored by miRNet 2.0 [74], an up to date comprehensive platform illustrating “multiple-to-multiple” relationships and functional interpretation via network-based visualization. We chose the TRRUST database [75] to capture the possible transcription factor-hub gene interactions in the current study, which offered 8,444 TF-target regulatory relationships of 800 human TFs derived from PubMed articles. Besides, we predicted the putative targeted miRNAs of all the hub genes with miRTarBase 8.0, which collected the most updated experimentally validated miRNA-target interactions (MTIs) that were validated by reporter assay, western blot, microarray, and next-generation sequencing experiments [76]. The transcription factor-hub gene interactions and predictive miRNA-hub genes interactions were visualized in Sankey diagrams.

### Semantic similarities and GSEA

The GOSemSim package [77] was used to compute the semantic similarities of the identified hub genes among GO terms with Wang's methods. The geometric mean in cellular components (CCs) and molecular functions (MFs) was adopted to evaluate the similarity index for each hub gene. The top three hub genes with the highest similarity index were further verified by GSEA using the gene expression profile of ICGC-LIRI-JP. Briefly, we calculated the Spearman correlation coefficients and corresponding *P* values between each of the genes in the profile and all of the three hub genes and selected the top 5000 of the most significantly correlated genes that had the largest correlation coefficients at a *P*-value of < 0.01. GSEA was performed using the clusterProfiler package [69] with the pre-ranked gene lists by correlation coefficients, which was based on the database of h.all.v7.0.symbols and c2.cp.kegg.v7.0.symbols, respectively.

### Network pharmacology analysis for HCV-HCC

The drug-gene interaction database (DGIdb) (https://dgidb.genome.wustl.edu/; version: 4.2.0 - sha1 afd9f30b) [78] was used to determine the promising drugs that target the identified hub genes. It contains more than 100,000 drug-gene interactions mined from PharmGKB, DrugBank, Chembl, TTD, Drug Target Commons, and others. Possible drugs were prefiltered by FDA approval and anti-neoplastic function. TCM-MESH [79] and TCM-ID [80] were used to predict the potential herbs and active components that targeting the hub genes. Only compounds supported by both of the two databases were regarded as potential effective herbal ingredients.

## Supplementary Material

Supplementary Figures

Supplementary Table 1

Supplementary Table 2

Supplementary Table 3

Supplementary Table 4

Supplementary Table 5

Supplementary Table 6
