# Supplementary material for: Integrative analysis identifies key mRNA biomarkers for diagnosis, prognosis, and therapeutic targets of HCV-associated hepatocellular carcinoma
Source: Aging (Albany NY). 2021 May 4;13(9):12865–95. doi: 10.18632/aging.202957 (PMC8148482; doi:10.18632/aging.202957)
Supplement: Supplementary Figures [file aging-13-202957-s001.pdf]

SUPPLEMENTARY FIGURES

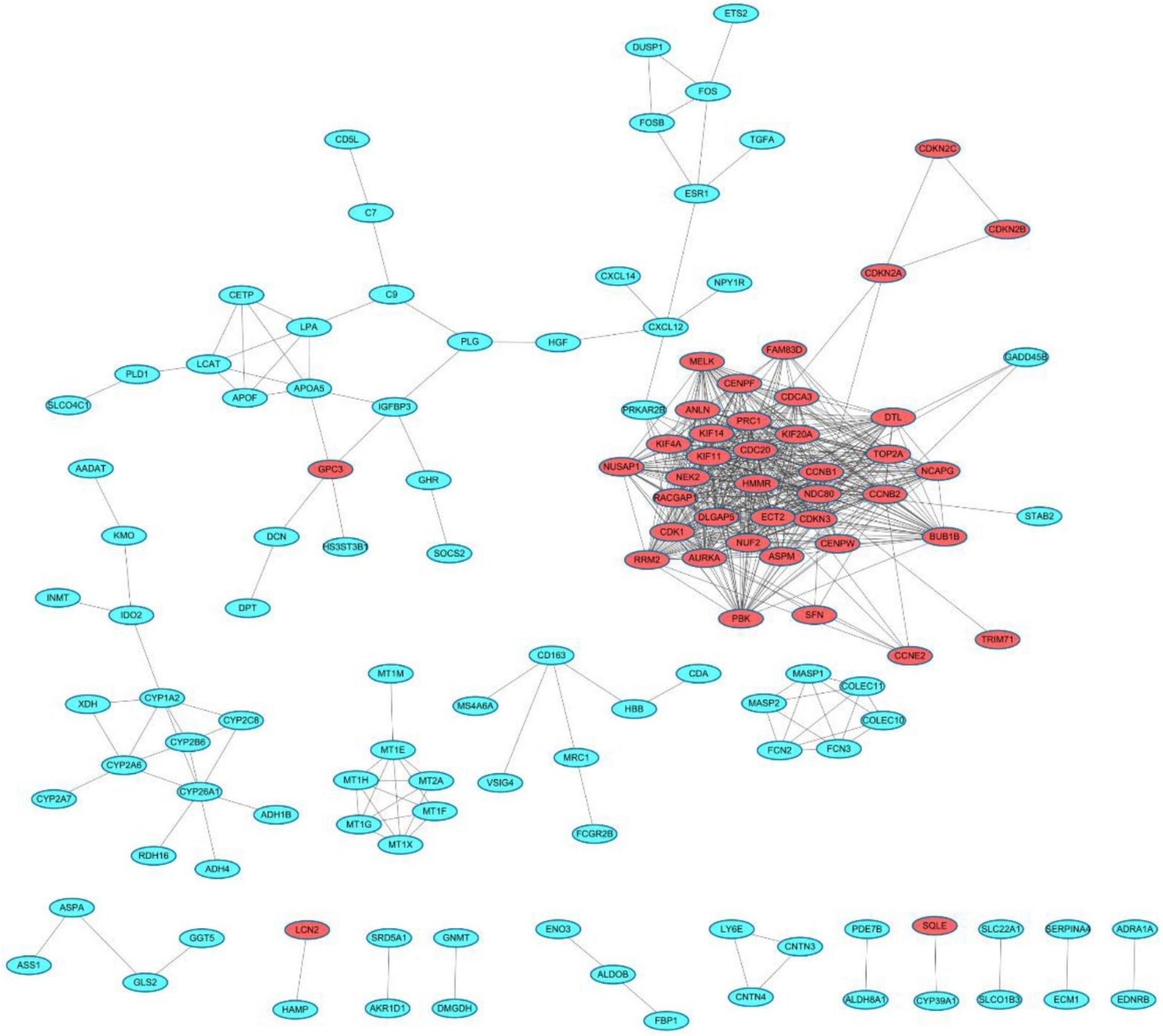

Supplementary Figure 1. PPI network of 240 DEGs based on the STRING database. Red nodes denote the upregulated genes, while blue nodes denote the downregulated genes. PPI, protein-protein interaction. DEGs, differentially expressed genes. PPI, protein-protein interaction. STRING, Search Tool for the Retrieval of Interacting Genes.

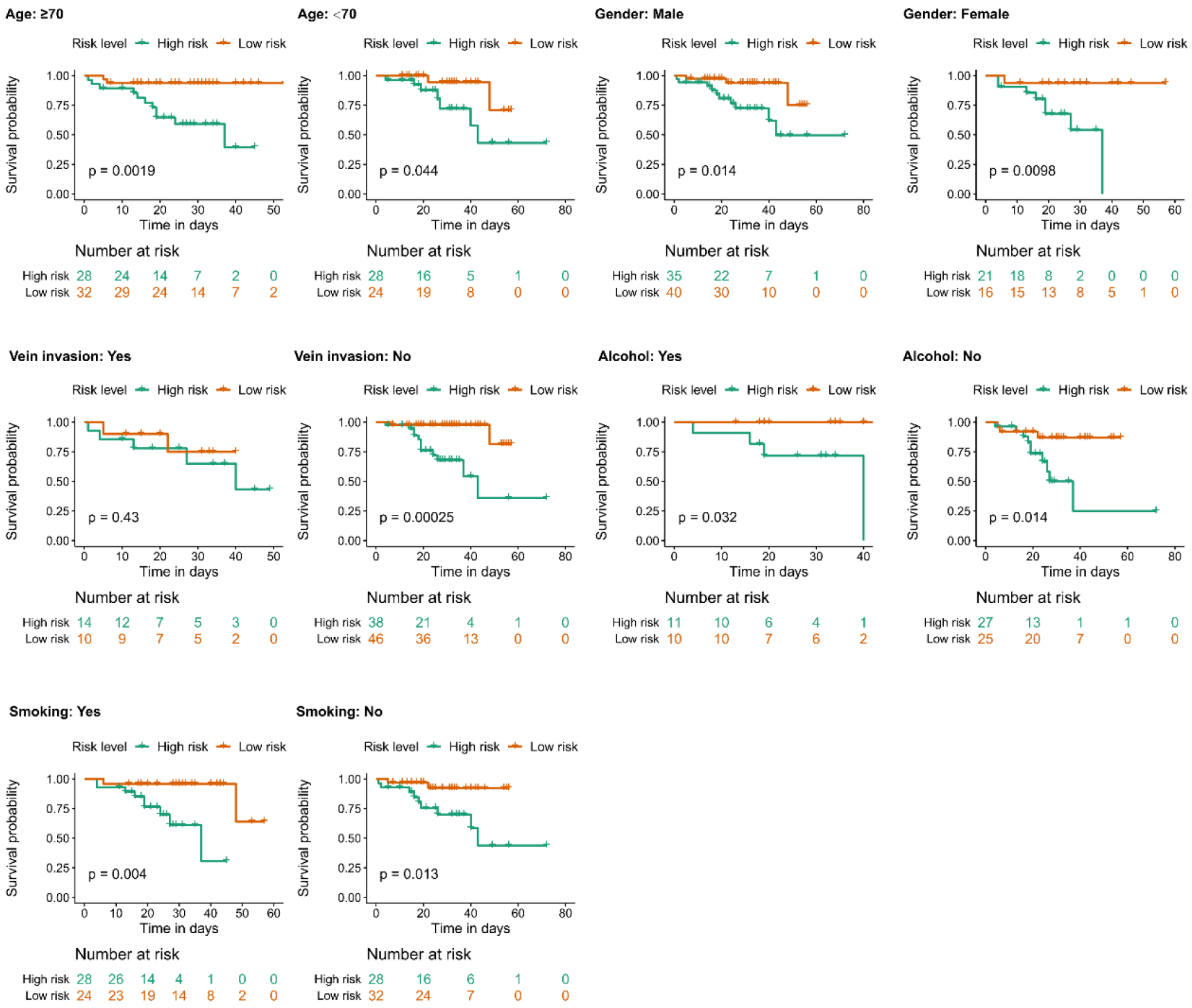

Supplementary Figure 2. Stratified survival analysis according to clinicopathological features.

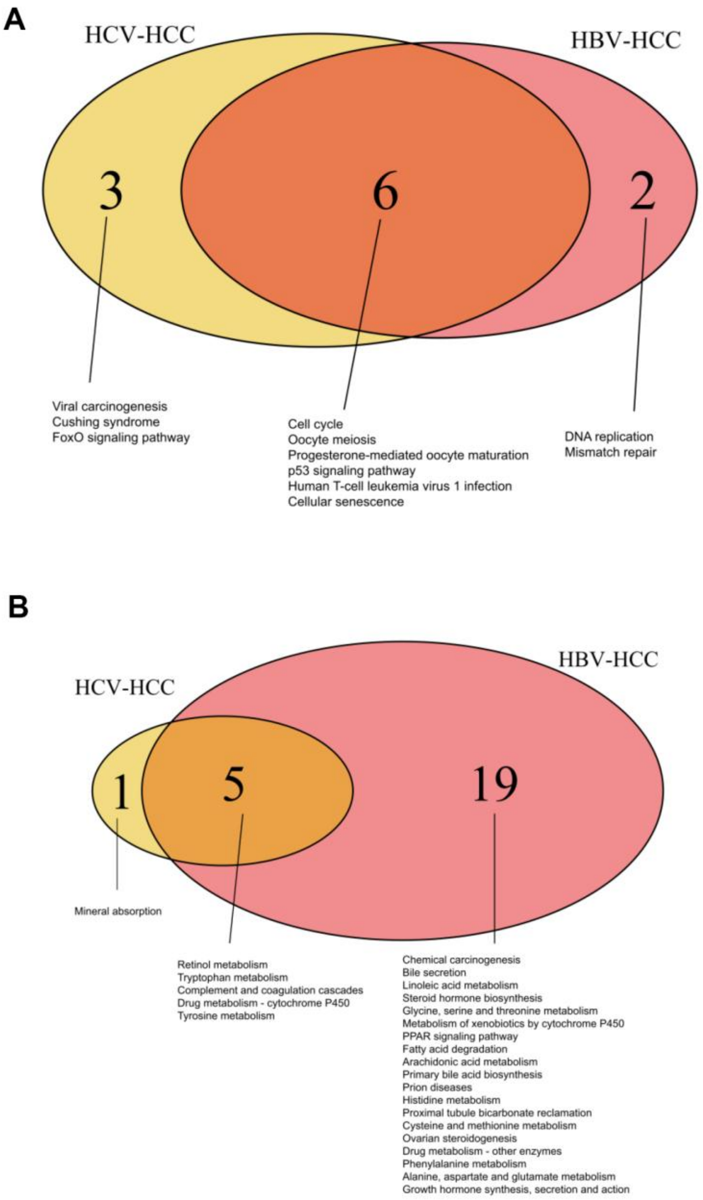

Supplementary Figure 3. Common KEGG pathways enriched by robust DEGs of HBV-HCC and HCV-HCC. (**A**) Venn plot showing the common or individual pathways enriched by the upregulated genes of HCV-HCC and HBV-HCC. (**B**) Venn plot showing the common or individual pathways enriched by the downregulated genes of HCV-HCC and HBV-HCC. HBV-HCC, HBV - associated HCC. HCV-HCC, HCV-associated HCC. KEGG, Kyoto Encyclopedia of Genes and Genomes. DEGs, differentially expressed genes.
