# Supplementary material for: Integrative analysis identifies key mRNA biomarkers for diagnosis, prognosis, and therapeutic targets of HCV-associated hepatocellular carcinoma
Source: Aging (Albany NY). 2021 May 4;13(9):12865–95. doi: 10.18632/aging.202957 (PMC8148482; doi:10.18632/aging.202957)
Supplement: Supplementary Table 1 [file aging-13-202957-s002.pdf]

Supplementary Table 1. Characteristics of all datasets enrolled in this study.

Study	Platforms	Etiology	Sample number			Technology
			Tumor	Normal	Total	
GSE6764	GPL570	HCV	35	10	45	microarray
GSE41804	GPL570	HCV	20	20	40	microarray
GSE62232	GPL570	HCV	9	10	19	microarray
GSE107170	GPL570	HCV	44	31	75	microarray
TCGA-LIHC	Illumina Hiseq	HCV	30	49	79	RNA-seq
HCCD-ICGC	Illumina Hiseq	HCV	123	98	221	RNA-seq
GSE12941	GPL5175	HCV	6	6	12	microarray
GSE69715	GPL570	HCV	37	66	103	microarray

HCV, Hepatitis C virus.
