# Supplementary material for: Integrative analysis identifies key mRNA biomarkers for diagnosis, prognosis, and therapeutic targets of HCV-associated hepatocellular carcinoma
Source: Aging (Albany NY). 2021 May 4;13(9):12865–95. doi: 10.18632/aging.202957 (PMC8148482; doi:10.18632/aging.202957)
Supplement: Supplementary Table 2 [file aging-13-202957-s003.doc]

## Supplementary Table 2. Differential analysis of the overlapping 240 DEGs by the combined microarray dataset.

|  | **logFC** | **AveExpr** | **t** | **P.Value** | **adj.P.Val** | **B** |
| --- | --- | --- | --- | --- | --- | --- |
| IGF2BP3 | 1.556232652 | 5.404083107 | 6.429049649 | 1.07E-09 | 1.07E-09 | 11.07386353 |
| TRIM71 | 1.556335056 | 4.932612078 | 6.607178352 | 4.07E-10 | 4.08E-10 | 12.02007204 |
| CYP2A7 | -1.897722399 | 9.334535697 | -6.665924422 | 2.95E-10 | 2.97E-10 | 12.33563382 |
| ADH1B | -1.660511164 | 11.28492994 | -6.754821731 | 1.81E-10 | 1.83E-10 | 12.81638894 |
| RAB3B | 1.426220641 | 4.70601103 | 7.073835386 | 3.03E-11 | 3.08E-11 | 14.57259994 |
| SLCO1B3 | -2.818878704 | 9.073949818 | -7.280045363 | 9.31E-12 | 9.51E-12 | 15.73246122 |
| TAT | -1.345812576 | 10.87834674 | -7.336941961 | 6.71E-12 | 6.88E-12 | 16.05574393 |
| LCN2 | 2.100059611 | 8.104668716 | 7.363692472 | 5.74E-12 | 5.92E-12 | 16.20821472 |
| VSIG4 | -1.204024923 | 7.400331814 | -7.538925516 | 2.07E-12 | 2.14E-12 | 17.21437536 |
| DUSP1 | -1.328937923 | 11.18349024 | -7.546038905 | 1.98E-12 | 2.06E-12 | 17.25548524 |
| CYP2C8 | -1.537276357 | 9.494804485 | -7.548077498 | 1.96E-12 | 2.05E-12 | 17.2672705 |
| MRAP2 | 1.354485564 | 5.100104253 | 7.558767346 | 1.84E-12 | 1.93E-12 | 17.32909674 |
| RNF125 | -1.15629579 | 7.992333767 | -7.568906145 | 1.74E-12 | 1.83E-12 | 17.3877784 |
| FXYD1 | -1.475959483 | 8.161294561 | -7.621747209 | 1.27E-12 | 1.34E-12 | 17.69427937 |
| KIF11 | 1.244789319 | 4.896008983 | 7.686195705 | 8.69E-13 | 9.23E-13 | 18.06960488 |
| MASP2 | -1.367839277 | 9.380757398 | -7.770102734 | 5.28E-13 | 5.64E-13 | 18.56067353 |
| GNMT | -2.079144376 | 9.120094385 | -7.817992601 | 3.97E-13 | 4.26E-13 | 18.84215929 |
| PPP1R3B | -1.384063972 | 9.718084792 | -7.953302791 | 1.77E-13 | 1.90E-13 | 19.64212257 |
| GSPT2 | -1.86452077 | 6.822721057 | -8.011809557 | 1.24E-13 | 1.34E-13 | 19.99009781 |
| SQLE | 1.543569436 | 8.491401519 | 8.055479997 | 9.54E-14 | 1.04E-13 | 20.25063379 |
| CYP2A6 | -2.343234462 | 10.02418625 | -8.103505059 | 7.13E-14 | 7.78E-14 | 20.53792971 |
| DAB1 | -1.316631607 | 6.036613645 | -8.125409794 | 6.24E-14 | 6.84E-14 | 20.66923716 |
| ALDH8A1 | -1.521145872 | 11.5727417 | -8.165095346 | 4.90E-14 | 5.40E-14 | 20.90755598 |
| GGT5 | -1.130177146 | 6.446453952 | -8.23578704 | 3.19E-14 | 3.53E-14 | 21.33341184 |
| IRX3 | 1.737915186 | 5.971626636 | 8.263053241 | 2.70E-14 | 3.00E-14 | 21.49811984 |
| CD163 | -1.300221366 | 9.743284167 | -8.34553342 | 1.63E-14 | 1.82E-14 | 21.99787077 |
| SLC4A4 | -1.461717033 | 7.588830076 | -8.382462417 | 1.30E-14 | 1.45E-14 | 22.22235074 |
| APOA5 | -1.999910694 | 9.341267789 | -8.405331732 | 1.13E-14 | 1.27E-14 | 22.36158831 |
| FCGR2B | -1.682572553 | 6.468185268 | -8.501369259 | 6.22E-15 | 7.05E-15 | 22.9481308 |
| EML6 | 1.262723268 | 6.040508416 | 8.541718905 | 4.85E-15 | 5.51E-15 | 23.19543096 |
| PLD1 | -1.16501348 | 6.410375706 | -8.556476523 | 4.42E-15 | 5.05E-15 | 23.28600573 |
| DMGDH | -1.289190854 | 6.675349181 | -8.57146688 | 4.03E-15 | 4.63E-15 | 23.37807776 |
| CDCA3 | 1.278163082 | 5.531889637 | 8.600911438 | 3.36E-15 | 3.87E-15 | 23.55912949 |
| GREM2 | -1.566358082 | 5.622314367 | -8.604051377 | 3.29E-15 | 3.82E-15 | 23.5784523 |
| PHYHIPL | 1.325269095 | 6.486444632 | 8.636691484 | 2.69E-15 | 3.13E-15 | 23.77949289 |
| LHX2 | -1.415039844 | 5.421165292 | -8.639949942 | 2.63E-15 | 3.08E-15 | 23.79958044 |
| SEMA6D | -1.363060321 | 6.425167657 | -8.742258816 | 1.39E-15 | 1.63E-15 | 24.43189939 |
| PON3 | -1.329628808 | 11.83954171 | -8.756474826 | 1.27E-15 | 1.50E-15 | 24.52000554 |
| CD5L | -1.813709253 | 8.121592325 | -8.763620311 | 1.22E-15 | 1.44E-15 | 24.56431313 |
| CDC20 | 1.56323807 | 5.551322973 | 8.771942858 | 1.15E-15 | 1.38E-15 | 24.61593812 |
| AZGP1 | -1.27778989 | 12.37110079 | -8.82951142 | 8.04E-16 | 9.64E-16 | 24.97358433 |
| CDA | -1.606980881 | 6.424649737 | -8.829921285 | 8.01E-16 | 9.64E-16 | 24.97613402 |
| AKR1D1 | -2.581571791 | 9.897578042 | -8.894239687 | 5.35E-16 | 6.48E-16 | 25.37683667 |
| CCNE2 | 1.332470039 | 5.876645988 | 8.903046694 | 5.06E-16 | 6.16E-16 | 25.4317946 |
| GLYAT | -2.234070758 | 8.857052149 | -8.928719585 | 4.30E-16 | 5.27E-16 | 25.59212291 |
| HOXA13 | 2.038265304 | 4.12958729 | 8.940858631 | 3.98E-16 | 4.90E-16 | 25.66799527 |
| CYP26A1 | -2.155152729 | 5.856448832 | -8.946804406 | 3.84E-16 | 4.75E-16 | 25.7051728 |
| DPT | -1.82124314 | 6.902449293 | -8.956297173 | 3.61E-16 | 4.49E-16 | 25.76454895 |
| PLG | -1.405784966 | 11.61227499 | -8.957964837 | 3.58E-16 | 4.47E-16 | 25.77498254 |
| NUF2 | 1.794848671 | 4.637121407 | 9.003595108 | 2.68E-16 | 3.37E-16 | 26.06075849 |
| KCND3 | -2.055047688 | 7.234322139 | -9.050502411 | 1.99E-16 | 2.51E-16 | 26.35511805 |
| COLEC11 | -2.015256415 | 8.922659438 | -9.066804398 | 1.80E-16 | 2.28E-16 | 26.45755615 |
| SFN | 1.961356768 | 8.395776048 | 9.111469733 | 1.35E-16 | 1.73E-16 | 26.73858249 |
| INMT | -1.846603773 | 6.90932364 | -9.117937002 | 1.30E-16 | 1.66E-16 | 26.77931671 |
| SERPINA4 | -1.555271727 | 9.995892114 | -9.208057701 | 7.31E-17 | 9.43E-17 | 27.3480668 |
| SKAP1 | -1.232036872 | 7.362958773 | -9.228829025 | 6.40E-17 | 8.30E-17 | 27.47944773 |
| C9 | -3.688802944 | 10.61738304 | -9.294421081 | 4.21E-17 | 5.49E-17 | 27.89503288 |
| GJC1 | 1.371602431 | 5.791468967 | 9.321739299 | 3.53E-17 | 4.63E-17 | 28.06843192 |
| C7 | -1.795753834 | 6.720879317 | -9.337570192 | 3.19E-17 | 4.21E-17 | 28.16899991 |
| SHBG | -1.693576102 | 6.764635582 | -9.371735161 | 2.56E-17 | 3.40E-17 | 28.38624431 |
| TP53I3 | 1.337266539 | 7.745381329 | 9.37652007 | 2.48E-17 | 3.31E-17 | 28.41669246 |
| CCNB2 | 1.637677607 | 6.135888821 | 9.389640641 | 2.28E-17 | 3.06E-17 | 28.50021161 |
| KIF4A | 1.639768299 | 5.522957136 | 9.403070116 | 2.10E-17 | 2.83E-17 | 28.58573956 |
| PTPRS | -1.616339783 | 7.145708432 | -9.44258532 | 1.62E-17 | 2.20E-17 | 28.83764634 |
| HBB | -1.967808638 | 10.05293491 | -9.533496863 | 9.04E-18 | 1.23E-17 | 29.41858064 |
| ZIC2 | 2.218733202 | 4.760727827 | 9.542526518 | 8.53E-18 | 1.17E-17 | 29.4763844 |
| SLC22A1 | -3.014430538 | 9.968151153 | -9.568642148 | 7.21E-18 | 9.94E-18 | 29.64366841 |
| CD1D | -1.775933552 | 6.86985959 | -9.575943463 | 6.87E-18 | 9.54E-18 | 29.69046447 |
| GADD45B | -1.414841812 | 9.734469025 | -9.576949515 | 6.83E-18 | 9.53E-18 | 29.69691346 |
| NDRG2 | -1.299379397 | 10.12992469 | -9.578386597 | 6.77E-18 | 9.50E-18 | 29.70612583 |
| SPINK1 | 4.296146571 | 8.912186972 | 9.59150033 | 6.22E-18 | 8.78E-18 | 29.79021226 |
| PBK | 1.997437074 | 5.303647381 | 9.61707242 | 5.27E-18 | 7.48E-18 | 29.95429261 |
| SOCS2 | -1.788423392 | 8.572484503 | -9.625646081 | 4.98E-18 | 7.12E-18 | 30.00933682 |
| ESR1 | -1.768768809 | 6.7140126 | -9.639842272 | 4.55E-18 | 6.53E-18 | 30.10051401 |
| MS4A6A | -1.366930519 | 8.816347403 | -9.641033429 | 4.51E-18 | 6.52E-18 | 30.10816641 |
| SYTL5 | -1.417178011 | 6.109285693 | -9.665079837 | 3.86E-18 | 5.61E-18 | 30.26271473 |
| ASPA | -1.552770121 | 6.767963376 | -9.675476269 | 3.61E-18 | 5.28E-18 | 30.32957234 |
| HAMP | -3.453088607 | 9.161789145 | -9.695739636 | 3.16E-18 | 4.66E-18 | 30.45994941 |
| GCDH | -1.081264868 | 8.601057049 | -9.765513657 | 2.01E-18 | 2.98E-18 | 30.9095534 |
| BCO2 | -2.138733666 | 8.026663115 | -9.876434352 | 9.76E-19 | 1.45E-18 | 31.62637862 |
| DLGAP5 | 1.921764687 | 4.763057107 | 9.890103798 | 8.93E-19 | 1.34E-18 | 31.71489035 |
| RRM2 | 2.156181301 | 7.595297755 | 9.899012549 | 8.42E-19 | 1.27E-18 | 31.77259595 |
| GRAMD1C | -1.695315794 | 8.074729007 | -9.919442049 | 7.37E-19 | 1.12E-18 | 31.90498565 |
| ADH4 | -1.751621357 | 10.10870416 | -9.927560475 | 6.99E-19 | 1.07E-18 | 31.95761857 |
| N4BP2L1 | -1.095583732 | 9.336794776 | -9.975862722 | 5.10E-19 | 7.84E-19 | 32.27103579 |
| NCAPG | 1.492464609 | 5.785782674 | 9.987250784 | 4.73E-19 | 7.32E-19 | 32.34499515 |
| IDO2 | -2.304334242 | 5.733161558 | -10.00973348 | 4.08E-19 | 6.36E-19 | 32.49108134 |
| SMPX | 2.527553385 | 5.013008717 | 10.03305184 | 3.50E-19 | 5.50E-19 | 32.64269936 |
| CYP2B6 | -1.91396062 | 9.643173028 | -10.05401922 | 3.05E-19 | 4.82E-19 | 32.77911889 |
| ECT2 | 1.731936118 | 6.49956388 | 10.1004776 | 2.25E-19 | 3.58E-19 | 33.08168276 |
| CNTN4 | -1.554137477 | 5.398953974 | -10.11594145 | 2.03E-19 | 3.25E-19 | 33.18248087 |
| ZG16 | -1.846514961 | 7.480681268 | -10.12691985 | 1.89E-19 | 3.05E-19 | 33.25406802 |
| NUSAP1 | 1.557976135 | 8.162877248 | 10.12995349 | 1.85E-19 | 3.01E-19 | 33.27385342 |
| DCN | -2.6928354 | 7.978559518 | -10.14229897 | 1.71E-19 | 2.79E-19 | 33.35438803 |
| SLC38A4 | -1.559427254 | 9.042712231 | -10.17827699 | 1.35E-19 | 2.22E-19 | 33.58924428 |
| HS3ST3B1 | -1.291268145 | 9.265560966 | -10.19164782 | 1.24E-19 | 2.05E-19 | 33.67658508 |
| DLG5 | 1.54643168 | 7.561724922 | 10.20327642 | 1.15E-19 | 1.91E-19 | 33.75257115 |
| BUB1B | 1.708357473 | 6.328805058 | 10.23282426 | 9.43E-20 | 1.58E-19 | 33.94575636 |
| NR4A3 | -1.630705514 | 6.332533988 | -10.24518294 | 8.69E-20 | 1.47E-19 | 34.02660337 |
| CDK1 | 2.146374409 | 6.148117445 | 10.30932051 | 5.69E-20 | 9.69E-20 | 34.44659704 |
| ADRA1A | -1.719498334 | 7.174940983 | -10.34529786 | 4.49E-20 | 7.69E-20 | 34.68249472 |
| CNTN3 | -2.791897919 | 5.48023989 | -10.35148501 | 4.31E-20 | 7.44E-20 | 34.72308478 |
| DTL | 1.894095286 | 5.893760659 | 10.36896655 | 3.84E-20 | 6.67E-20 | 34.83780459 |
| NEK2 | 2.017096862 | 5.51701812 | 10.36900762 | 3.84E-20 | 6.67E-20 | 34.83807417 |
| CLRN3 | -3.071826923 | 7.806919483 | -10.37685107 | 3.64E-20 | 6.43E-20 | 34.8895621 |
| EPB41L4B | -1.394444979 | 6.978360623 | -10.38978078 | 3.34E-20 | 5.94E-20 | 34.97446056 |
| ANK3 | -2.151394575 | 6.299920153 | -10.4999176 | 1.61E-20 | 2.89E-20 | 35.69873173 |
| BBOX1 | -2.650613496 | 7.256547611 | -10.50229194 | 1.59E-20 | 2.86E-20 | 35.71436685 |
| OLFML3 | -2.029270735 | 7.518154346 | -10.50479621 | 1.56E-20 | 2.84E-20 | 35.73085852 |
| ANTXR2 | -1.117322391 | 7.859780448 | -10.56168412 | 1.07E-20 | 1.96E-20 | 36.10575082 |
| AMDHD1 | -1.433967643 | 11.05203104 | -10.6294914 | 6.82E-21 | 1.26E-20 | 36.55324464 |
| MT2A | -1.589143172 | 13.01716255 | -10.63449111 | 6.60E-21 | 1.23E-20 | 36.5862674 |
| NDC80 | 1.630197735 | 6.139143355 | 10.64350038 | 6.21E-21 | 1.16E-20 | 36.64578222 |
| TTC36 | -2.378213678 | 7.543306546 | -10.64633688 | 6.10E-21 | 1.15E-20 | 36.66452252 |
| FEZ1 | -1.263235321 | 6.713179882 | -10.67145 | 5.16E-21 | 9.82E-21 | 36.83049183 |
| RDH16 | -2.185680564 | 10.49866063 | -10.67325865 | 5.10E-21 | 9.79E-21 | 36.84244847 |
| SRD5A1 | -1.432163815 | 8.325645378 | -10.67935524 | 4.89E-21 | 9.47E-21 | 36.88275548 |
| RBMS3 | -1.661337294 | 6.858532124 | -10.68469835 | 4.72E-21 | 9.21E-21 | 36.91808537 |
| DIRAS3 | -1.776302652 | 5.413111473 | -10.70648683 | 4.08E-21 | 8.04E-21 | 37.06219819 |
| ETS2 | -1.264155212 | 9.85123581 | -10.72423435 | 3.63E-21 | 7.20E-21 | 37.17963354 |
| AURKA | 1.702472954 | 7.255101399 | 10.73484586 | 3.38E-21 | 6.76E-21 | 37.24987119 |
| LPA | -2.201053186 | 8.163196879 | -10.84055116 | 1.67E-21 | 3.37E-21 | 37.95038882 |
| ACSM3 | -1.822289971 | 8.287698013 | -10.85228482 | 1.54E-21 | 3.14E-21 | 38.02824252 |
| COL15A1 | 2.565506216 | 6.366383877 | 10.86404371 | 1.43E-21 | 2.93E-21 | 38.10628203 |
| GPR180 | -1.123507052 | 6.118962732 | -10.87815134 | 1.30E-21 | 2.69E-21 | 38.19993342 |
| RBM24 | 2.130169533 | 5.773366541 | 10.87865552 | 1.29E-21 | 2.69E-21 | 38.20328082 |
| ZGPAT | -1.792885124 | 8.993341529 | -10.87943154 | 1.29E-21 | 2.69E-21 | 38.20843314 |
| KIF20A | 1.976504068 | 5.241849861 | 10.88231951 | 1.26E-21 | 2.68E-21 | 38.22760823 |
| CETP | -2.019507211 | 6.418804306 | -10.91141726 | 1.04E-21 | 2.23E-21 | 38.42086771 |
| PAMR1 | -1.684867178 | 6.35699442 | -10.96549482 | 7.24E-22 | 1.57E-21 | 38.78032673 |
| LCAT | -1.913438776 | 8.036452367 | -10.96831894 | 7.11E-22 | 1.55E-21 | 38.79910914 |
| ENO3 | -2.07614676 | 7.763912028 | -10.98613567 | 6.31E-22 | 1.39E-21 | 38.91762641 |
| MOGAT2 | -1.823812975 | 6.917254424 | -10.98698019 | 6.27E-22 | 1.39E-21 | 38.92324515 |
| DNASE1L3 | -2.302911931 | 9.253908596 | -11.04459688 | 4.26E-22 | 9.57E-22 | 39.30678812 |
| MELK | 1.832458164 | 6.91069417 | 11.0701133 | 3.59E-22 | 8.14E-22 | 39.47677528 |
| ITGA9 | -1.691336285 | 8.258212531 | -11.07398514 | 3.50E-22 | 8.00E-22 | 39.50257584 |
| ST6GAL2 | -1.641666251 | 4.579209827 | -11.0742283 | 3.50E-22 | 8.00E-22 | 39.50419622 |
| TMEM56 | -1.139037253 | 10.47495019 | -11.10622075 | 2.82E-22 | 6.57E-22 | 39.7174515 |
| MCC | -1.946519598 | 7.472312546 | -11.13077802 | 2.39E-22 | 5.63E-22 | 39.88122707 |
| GHR | -1.991069863 | 10.50939977 | -11.15498375 | 2.03E-22 | 4.83E-22 | 40.04272669 |
| C8orf4 | -1.67548213 | 9.808368374 | -11.16946495 | 1.85E-22 | 4.43E-22 | 40.13937684 |
| XDH | -1.914214113 | 9.379945744 | -11.19137232 | 1.59E-22 | 3.86E-22 | 40.28563578 |
| CDKN2B | 1.455622061 | 5.363303723 | 11.22329397 | 1.29E-22 | 3.15E-22 | 40.49884894 |
| CDKN3 | 1.881748997 | 6.086682811 | 11.22830332 | 1.24E-22 | 3.07E-22 | 40.53231796 |
| MYO10 | -1.32830958 | 5.53776295 | -11.23575935 | 1.18E-22 | 2.95E-22 | 40.58213917 |
| CCNB1 | 2.280341146 | 6.040118709 | 11.23960069 | 1.15E-22 | 2.91E-22 | 40.60780938 |
| ANLN | 2.249506625 | 5.428780763 | 11.28802322 | 8.31E-23 | 2.12E-22 | 40.93153632 |
| COLEC10 | -2.090890918 | 5.749158896 | -11.29003931 | 8.20E-23 | 2.12E-22 | 40.94502032 |
| CDKN2A | 1.377257698 | 6.487702473 | 11.29038664 | 8.18E-23 | 2.12E-22 | 40.94734338 |
| APOF | -2.975793139 | 9.226944012 | -11.31220765 | 7.07E-23 | 1.86E-22 | 41.09331446 |
| KIF14 | 1.842237828 | 5.139779574 | 11.32622167 | 6.43E-23 | 1.71E-22 | 41.18708737 |
| CYP4V2 | -1.445507416 | 9.952471329 | -11.32863788 | 6.33E-23 | 1.71E-22 | 41.20325725 |
| HAO2 | -2.600337435 | 7.489371785 | -11.33181497 | 6.19E-23 | 1.69E-22 | 41.22451998 |
| MT1H | -2.08653347 | 11.48441852 | -11.33963624 | 5.87E-23 | 1.62E-22 | 41.27686839 |
| CDKN2C | 1.556971719 | 6.885695179 | 11.3557269 | 5.27E-23 | 1.47E-22 | 41.38458453 |
| MT1X | -1.976529473 | 11.95023504 | -11.36339578 | 5.01E-23 | 1.41E-22 | 41.43593186 |
| MFSD2A | -2.739612681 | 7.820337625 | -11.39518625 | 4.04E-23 | 1.15E-22 | 41.6488503 |
| PRC1 | 1.903077104 | 6.312714155 | 11.41978209 | 3.42E-23 | 9.90E-23 | 41.81365209 |
| CENPW | 1.669759874 | 6.802907119 | 11.5957279 | 1.04E-23 | 3.06E-23 | 42.99425402 |
| KBTBD11 | -1.780188821 | 5.916699019 | -11.59973918 | 1.02E-23 | 3.01E-23 | 43.0212032 |
| NRG1 | -2.609155165 | 5.404874058 | -11.66303716 | 6.62E-24 | 1.99E-23 | 43.44664718 |
| ASPM | 2.049308743 | 5.982135201 | 11.6728229 | 6.20E-24 | 1.88E-23 | 43.51245086 |
| EDNRB | -1.38007471 | 7.31269287 | -11.70503946 | 4.98E-24 | 1.53E-23 | 43.72914639 |
| GBA3 | -2.720218292 | 9.023320771 | -11.71871057 | 4.54E-24 | 1.42E-23 | 43.82112745 |
| PROM1 | -2.723956387 | 4.661283302 | -11.76147133 | 3.40E-24 | 1.07E-23 | 44.10892604 |
| CRHBP | -3.354340653 | 6.630601567 | -11.79198821 | 2.77E-24 | 8.85E-24 | 44.31440771 |
| THRSP | -3.243807063 | 7.91330501 | -11.89013593 | 1.42E-24 | 4.61E-24 | 44.97576062 |
| FOS | -2.646344588 | 8.343311791 | -11.92442381 | 1.13E-24 | 3.71E-24 | 45.20697279 |
| SPG20 | -1.453739736 | 5.41216195 | -11.94234708 | 9.99E-25 | 3.33E-24 | 45.32786757 |
| TSPYL5 | -1.343289325 | 5.804636363 | -11.99140938 | 7.16E-25 | 2.42E-24 | 45.65891412 |
| MT1E | -1.939240487 | 11.50656119 | -12.01226118 | 6.21E-25 | 2.13E-24 | 45.79966088 |
| CDH19 | -2.499849082 | 5.295907727 | -12.02415385 | 5.73E-25 | 1.99E-24 | 45.87994782 |
| GPC3 | 3.770275618 | 8.999590134 | 12.06860676 | 4.24E-25 | 1.50E-24 | 46.18012985 |
| PLAC8 | -2.18358056 | 6.875323746 | -12.09188881 | 3.62E-25 | 1.30E-24 | 46.33739974 |
| MARCO | -2.923176891 | 6.924612815 | -12.16805378 | 2.16E-25 | 7.84E-25 | 46.85212746 |
| TOP2A | 2.615239571 | 6.477928895 | 12.1795765 | 1.99E-25 | 7.36E-25 | 46.93002905 |
| MRC1 | -1.790808093 | 9.25844928 | -12.18707717 | 1.90E-25 | 7.11E-25 | 46.98074297 |
| SRPX | -3.01089752 | 6.690164521 | -12.19345703 | 1.81E-25 | 6.91E-25 | 47.02388134 |
| NPY1R | -2.23343817 | 5.739518236 | -12.21520902 | 1.57E-25 | 6.06E-25 | 47.17097833 |
| MT1G | -2.289822214 | 11.64477331 | -12.25856436 | 1.17E-25 | 4.59E-25 | 47.46424659 |
| ALDOB | -2.155374715 | 9.307000533 | -12.27733756 | 1.03E-25 | 4.10E-25 | 47.59126598 |
| GSTZ1 | -1.786317402 | 8.641766776 | -12.3362798 | 6.87E-26 | 2.79E-25 | 47.99019023 |
| AADAT | -1.781732892 | 7.305717868 | -12.34510042 | 6.47E-26 | 2.68E-25 | 48.04990408 |
| STAB2 | -1.949870396 | 5.912028452 | -12.35219226 | 6.16E-26 | 2.60E-25 | 48.0979172 |
| PDE7B | -1.568207269 | 6.185563559 | -12.52327814 | 1.92E-26 | 8.24E-26 | 49.25692433 |
| FAM83D | 2.185425225 | 6.466924127 | 12.53011761 | 1.84E-26 | 8.01E-26 | 49.30328475 |
| KMO | -2.406291033 | 8.879105361 | -12.56105269 | 1.49E-26 | 6.61E-26 | 49.51299829 |
| GCH1 | -1.550955675 | 10.74497316 | -12.5828064 | 1.28E-26 | 5.80E-26 | 49.66049296 |
| TFPI2 | -2.383842848 | 5.379020549 | -12.60512501 | 1.10E-26 | 5.08E-26 | 49.81183704 |
| CHST4 | -2.526425412 | 6.163406838 | -12.63829265 | 8.78E-27 | 4.13E-26 | 50.03678405 |
| FOSB | -3.013491852 | 7.729100582 | -12.67928025 | 6.64E-27 | 3.19E-26 | 50.31482246 |
| RND3 | -1.783456049 | 10.35712675 | -12.68331172 | 6.46E-27 | 3.16E-26 | 50.3421731 |
| HMMR | 2.252043172 | 6.472116704 | 12.71135593 | 5.34E-27 | 2.67E-26 | 50.53244799 |
| GLS2 | -3.427053128 | 7.605238966 | -12.84153198 | 2.20E-27 | 1.12E-26 | 51.41599669 |
| SLCO4C1 | -2.145360621 | 5.371932263 | -12.86832014 | 1.83E-27 | 9.54E-27 | 51.59787811 |
| LY6E | -2.234723891 | 7.726636212 | -12.88838605 | 1.59E-27 | 8.51E-27 | 51.73413052 |
| HHIP | -2.526525962 | 5.592779055 | -12.89137173 | 1.56E-27 | 8.51E-27 | 51.75440487 |
| IL1RAP | -2.357747321 | 7.957629373 | -12.92873499 | 1.21E-27 | 6.76E-27 | 52.00814038 |
| HGF | -2.961343144 | 7.447920962 | -12.93818591 | 1.14E-27 | 6.49E-27 | 52.07232736 |
| MASP1 | -1.59564868 | 8.085279414 | -12.94730907 | 1.07E-27 | 6.24E-27 | 52.1342903 |
| PCDH9 | -1.910232021 | 5.721134566 | -13.05140762 | 5.24E-28 | 3.14E-27 | 52.84143763 |
| CYP1A2 | -3.193187357 | 9.056479418 | -13.06739436 | 4.70E-28 | 2.89E-27 | 52.95005559 |
| CYP39A1 | -2.562741932 | 7.669679558 | -13.0888826 | 4.06E-28 | 2.56E-27 | 53.09605931 |
| MT1M | -3.430690157 | 7.241096642 | -13.20581325 | 1.83E-28 | 1.18E-27 | 53.89068296 |
| CENPF | 2.136232319 | 6.675066298 | 13.31001317 | 8.97E-29 | 5.98E-28 | 54.5989372 |
| TUBE1 | -1.805728125 | 8.146129639 | -13.34432534 | 7.09E-29 | 4.86E-28 | 54.83218114 |
| SLC7A2 | -1.82973977 | 10.14025022 | -13.43518529 | 3.81E-29 | 2.69E-28 | 55.44985517 |
| MAN1C1 | -1.622287894 | 7.179599687 | -13.45628074 | 3.30E-29 | 2.40E-28 | 55.59326893 |
| PRKAR2B | -1.861556785 | 5.323419054 | -13.59083694 | 1.32E-29 | 9.88E-29 | 56.508038 |
| TGFA | -1.689691457 | 5.807399147 | -13.59363982 | 1.29E-29 | 9.88E-29 | 56.52709296 |
| FCN3 | -3.402496087 | 8.385938583 | -13.63166129 | 9.97E-30 | 7.97E-29 | 56.78557418 |
| FBP1 | -2.188584848 | 10.84282972 | -13.68726008 | 6.82E-30 | 5.64E-29 | 57.1635403 |
| FLVCR1 | 1.569062336 | 7.161500174 | 13.69755908 | 6.36E-30 | 5.45E-29 | 57.23355213 |
| IGFBP3 | -2.384545137 | 9.713957771 | -13.7781244 | 3.67E-30 | 3.26E-29 | 57.78120227 |
| STEAP4 | -2.529905422 | 7.393287382 | -13.83957214 | 2.41E-30 | 2.22E-29 | 58.19885951 |
| CLEC1B | -3.556518282 | 6.580799933 | -14.00326557 | 7.89E-31 | 7.57E-30 | 59.31124344 |
| CCBE1 | -2.377587292 | 5.804826423 | -14.08361727 | 4.56E-31 | 4.56E-30 | 59.85711817 |
| CXCL12 | -2.466252654 | 8.876950296 | -14.09745151 | 4.15E-31 | 4.33E-30 | 59.95108965 |
| BDH2 | -1.417727947 | 8.477962922 | -14.13711088 | 3.16E-31 | 3.45E-30 | 60.22046106 |
| RSPO3 | -2.255834616 | 6.067106035 | -14.14594755 | 2.98E-31 | 3.40E-30 | 60.28047631 |
| OIT3 | -3.256638138 | 7.188668909 | -14.25240648 | 1.44E-31 | 1.73E-30 | 61.00336405 |
| PITPNM3 | -1.541949727 | 4.986850696 | -14.57020883 | 1.65E-32 | 2.09E-31 | 63.1594892 |
| FAM65C | -2.274969622 | 5.995449412 | -14.59460629 | 1.40E-32 | 1.87E-31 | 63.32487978 |
| MT1F | -2.980445138 | 10.22528176 | -14.69786758 | 6.94E-33 | 9.79E-32 | 64.02464869 |
| RACGAP1 | 2.054849793 | 7.491878588 | 14.87487683 | 2.08E-33 | 3.12E-32 | 65.22320784 |
| ADAMTS13 | -1.730366664 | 6.75571534 | -15.21149719 | 2.12E-34 | 3.39E-33 | 67.49862601 |
| ASS1 | -1.688777246 | 12.78561051 | -15.29477277 | 1.21E-34 | 2.07E-33 | 68.06065558 |
| GPM6A | -3.182652064 | 6.237211559 | -15.30457846 | 1.13E-34 | 2.07E-33 | 68.12680986 |
| ECM1 | -1.905009058 | 6.878895208 | -15.41548698 | 5.32E-35 | 1.06E-33 | 68.87468381 |
| LIFR | -1.955164062 | 6.76080388 | -15.60633765 | 1.46E-35 | 3.19E-34 | 70.1599457 |
| CLEC4G | -3.135425799 | 5.992841082 | -15.86311548 | 2.59E-36 | 6.21E-35 | 71.88559501 |
| TMEM27 | -3.24121751 | 6.858664613 | -15.95973181 | 1.35E-36 | 3.60E-35 | 72.53375944 |
| CLEC4M | -3.210778438 | 5.88559884 | -16.28933888 | 1.47E-37 | 4.42E-36 | 74.73996483 |
| TACSTD2 | -3.773283909 | 6.353128573 | -16.36644238 | 8.79E-38 | 3.01E-36 | 75.25488141 |
| DACH1 | -1.887979545 | 4.706738653 | -16.81212683 | 4.47E-39 | 1.79E-37 | 78.22195027 |
| FCN2 | -3.446015533 | 6.533654566 | -17.23507335 | 2.69E-40 | 1.29E-38 | 81.02194492 |
| CXCL14 | -3.891531242 | 5.876062308 | -18.52043733 | 5.82E-44 | 3.49E-42 | 89.42585201 |
| FREM2 | -3.392246487 | 4.575855845 | -19.07715963 | 1.59E-45 | 1.27E-43 | 93.01185919 |
| CAP2 | 2.410077924 | 8.102887857 | 20.00563932 | 4.24E-48 | 5.08E-46 | 98.91401012 |
| ANGPTL6 | -1.687294803 | 7.017970872 | -21.19133593 | 2.54E-51 | 6.09E-49 | 106.3013601 |

DEGs, differentially expressed genes.
