# Supplementary material for: Integrative analysis identifies key mRNA biomarkers for diagnosis, prognosis, and therapeutic targets of HCV-associated hepatocellular carcinoma
Source: Aging (Albany NY). 2021 May 4;13(9):12865–95. doi: 10.18632/aging.202957 (PMC8148482; doi:10.18632/aging.202957)
Supplement: Supplementary Table 3 [file aging-13-202957-s004.doc]

## Supplementary Table 3. DEGs of the ICGC-LIRI-JP dataset.

| **gene** | **logFC** | **AveExpr** | **t** | **P.Value** | **adj.P.Val** | **B** |
| --- | --- | --- | --- | --- | --- | --- |
| CXCL14 | -3.161830098 | 2.170452489 | -22.59907506 | 3.01E-59 | 6.44E-55 | 124.1972847 |
| CLEC4G | -3.746676622 | 2.48719457 | -22.25932252 | 3.07E-58 | 3.28E-54 | 121.901368 |
| TMEM27 | -2.175350091 | 2.26040724 | -21.97837038 | 2.12E-57 | 1.51E-53 | 119.9920634 |
| COLEC10 | -2.847896134 | 2.287420814 | -21.02243985 | 1.62E-54 | 8.64E-51 | 113.4244505 |
| FAM83F | -1.285403186 | 0.738778281 | -20.9878021 | 2.06E-54 | 8.81E-51 | 113.1844497 |
| TBCE | 1.280433051 | 3.706108597 | 20.83578884 | 5.98E-54 | 2.13E-50 | 112.129528 |
| CAP2 | 2.068117637 | 2.539095023 | 20.45127026 | 8.97E-53 | 2.74E-49 | 109.4493173 |
| CLEC1B | -3.107558487 | 2.054841629 | -20.41029803 | 1.20E-52 | 3.20E-49 | 109.1627462 |
| CLEC4M | -3.349493944 | 2.091312217 | -20.17522625 | 6.33E-52 | 1.50E-48 | 107.5149928 |
| FCN2 | -3.642585864 | 2.763800905 | -20.0927859 | 1.14E-51 | 2.43E-48 | 106.9356839 |
| SFRP5 | -2.705034843 | 1.578054299 | -19.81080565 | 8.45E-51 | 1.64E-47 | 104.948661 |
| ECM1 | -2.37244815 | 2.916832579 | -19.58691121 | 4.18E-50 | 7.44E-47 | 103.3649464 |
| CFP | -2.320550854 | 2.6239819 | -19.32197022 | 2.79E-49 | 4.59E-46 | 101.4841996 |
| FCN3 | -4.179323876 | 4.002624434 | -19.2780734 | 3.83E-49 | 5.84E-46 | 101.1719006 |
| STAB2 | -1.831304961 | 1.57678733 | -19.18695094 | 7.37E-49 | 1.05E-45 | 100.5230063 |
| GPM6A | -1.598844367 | 1.150859729 | -19.12445337 | 1.16E-48 | 1.54E-45 | 100.0774782 |
| KLHL12 | 1.042112992 | 3.25520362 | 18.87008941 | 7.23E-48 | 9.09E-45 | 98.26026526 |
| VIPR1 | -2.165761573 | 2.086968326 | -18.81406062 | 1.08E-47 | 1.29E-44 | 97.85915519 |
| CCBE1 | -1.094712958 | 0.725113122 | -18.78838738 | 1.31E-47 | 1.47E-44 | 97.67526156 |
| UBE2T | 1.926831757 | 2.06678733 | 18.74926961 | 1.73E-47 | 1.85E-44 | 97.39494811 |
| NPY1R | -1.626231956 | 1.467963801 | -18.65600811 | 3.40E-47 | 3.46E-44 | 96.72607273 |
| CRHBP | -3.438849345 | 2.92800905 | -18.55107566 | 7.28E-47 | 7.07E-44 | 95.9725373 |
| CCT3 | 1.371014601 | 6.188461538 | 18.44205016 | 1.61E-46 | 1.49E-43 | 95.18855424 |
| GDF2 | -2.055452132 | 1.177239819 | -18.33486537 | 3.50E-46 | 3.11E-43 | 94.41677766 |
| FAM189B | 1.361172225 | 2.204208145 | 18.09976626 | 1.94E-45 | 1.66E-42 | 92.72047871 |
| CXCL12 | -2.935679443 | 4.133257919 | -18.07932486 | 2.25E-45 | 1.85E-42 | 92.57276765 |
| FLAD1 | 1.165699353 | 3.966742081 | 17.95823954 | 5.44E-45 | 4.30E-42 | 91.6970877 |
| MARCO | -3.720993861 | 2.903529412 | -17.92129227 | 7.12E-45 | 5.44E-42 | 91.42964933 |
| LIFR | -1.783308445 | 1.951357466 | -17.89636326 | 8.55E-45 | 6.30E-42 | 91.24914141 |
| PLAC8 | -2.014769371 | 1.936923077 | -17.79552415 | 1.79E-44 | 1.27E-41 | 90.518472 |
| SCAMP3 | 1.308991206 | 4.666696833 | 17.70115028 | 3.56E-44 | 2.46E-41 | 89.83392596 |
| PSMD4 | 1.402069023 | 6.231357466 | 17.6899551 | 3.87E-44 | 2.58E-41 | 89.75267547 |
| MUC6 | -2.33136884 | 1.35918552 | -17.5122876 | 1.42E-43 | 9.21E-41 | 88.46196155 |
| BMPER | -1.386469222 | 1.019773756 | -17.41828076 | 2.84E-43 | 1.78E-40 | 87.77808018 |
| GBA | 1.564427576 | 3.936515837 | 17.27746256 | 7.98E-43 | 4.61E-40 | 86.75247707 |
| EIF2D | 1.085558321 | 4.146832579 | 17.27903161 | 7.89E-43 | 4.61E-40 | 86.76391232 |
| DPT | -3.046595321 | 2.406832579 | -17.2654359 | 8.72E-43 | 4.90E-40 | 86.66482074 |
| CDKN3 | 2.124052597 | 2.100226244 | 17.2593776 | 9.11E-43 | 4.99E-40 | 86.62066112 |
| ADAMTS13 | -1.702317903 | 2.412760181 | -17.23489529 | 1.09E-42 | 5.83E-40 | 86.44218116 |
| RSPO3 | -1.2969703 | 1.012850679 | -17.19608907 | 1.45E-42 | 7.56E-40 | 86.1591942 |
| GPR128 | -1.395478679 | 1.581085973 | -17.1633912 | 1.85E-42 | 9.39E-40 | 85.92067264 |
| SRPX | -1.887090592 | 1.709004525 | -17.10757036 | 2.79E-42 | 1.38E-39 | 85.51331159 |
| FAM180A | -1.489357889 | 1.303122172 | -16.96208671 | 8.13E-42 | 3.86E-39 | 84.45067414 |
| PAMR1 | -1.360294508 | 1.360361991 | -16.87184372 | 1.58E-41 | 7.19E-39 | 83.79085654 |
| CHST4 | -2.020148498 | 1.16719457 | -16.84865376 | 1.88E-41 | 8.36E-39 | 83.62122146 |
| IGFBP3 | -2.658105193 | 6.014479638 | -16.82612248 | 2.22E-41 | 9.47E-39 | 83.45637387 |
| ASPM | 1.399776008 | 1.224162896 | 16.82881281 | 2.17E-41 | 9.47E-39 | 83.47605899 |
| RACGAP1 | 1.469639124 | 1.821719457 | 16.62258994 | 9.97E-41 | 4.02E-38 | 81.96591447 |
| CNIH4 | 1.144625021 | 4.494298643 | 16.61342466 | 1.07E-40 | 4.14E-38 | 81.89874269 |
| CDHR2 | -2.723055417 | 2.481085973 | -16.56068756 | 1.58E-40 | 5.97E-38 | 81.51214687 |
| PTH1R | -2.09564294 | 2.231809955 | -16.52602182 | 2.04E-40 | 7.37E-38 | 81.25794431 |
| CDK5 | 1.130987224 | 2.936606335 | 16.50761298 | 2.33E-40 | 8.17E-38 | 81.12292727 |
| PIGC | 1.085214037 | 3.506334842 | 16.50159544 | 2.44E-40 | 8.41E-38 | 81.07878861 |
| CETP | -2.036242741 | 2.567420814 | -16.37026994 | 6.45E-40 | 2.19E-37 | 80.11505611 |
| AURKA | 1.87331923 | 2.141085973 | 16.31925026 | 9.41E-40 | 3.14E-37 | 79.74041781 |
| PPP2R5A | 1.004763564 | 4.927375566 | 16.27337534 | 1.32E-39 | 4.28E-37 | 79.40345162 |
| NAA20 | 1.066013771 | 5.438914027 | 16.20903603 | 2.13E-39 | 6.69E-37 | 78.93069433 |
| UBQLN4 | 1.129363697 | 3.480090498 | 16.16731175 | 2.90E-39 | 8.98E-37 | 78.62400988 |
| PYGO2 | 1.121235275 | 3.245158371 | 16.144635 | 3.43E-39 | 1.05E-36 | 78.45729763 |
| PTTG1 | 2.15560146 | 2.741764706 | 16.10930614 | 4.46E-39 | 1.34E-36 | 78.197527 |
| LY6E | -2.944368674 | 5.945972851 | -16.02990513 | 8.04E-39 | 2.29E-36 | 77.61350591 |
| PSMB4 | 1.055998839 | 7.087013575 | 16.0150256 | 8.97E-39 | 2.52E-36 | 77.50403342 |
| TACSTD2 | -1.974078314 | 1.294570136 | -15.98377962 | 1.13E-38 | 3.10E-36 | 77.27412002 |
| OIT3 | -2.062538576 | 3.336561086 | -15.97892936 | 1.17E-38 | 3.17E-36 | 77.23842756 |
| VPS72 | 1.083471877 | 3.893936652 | 15.96358404 | 1.31E-38 | 3.47E-36 | 77.12549713 |
| RAB25 | -2.122649743 | 1.240452489 | -15.9384397 | 1.58E-38 | 4.13E-36 | 76.94043345 |
| B3GAT1 | -1.426100879 | 1.067104072 | -15.83385233 | 3.44E-38 | 8.73E-36 | 76.17041337 |
| REPIN1 | 1.010729219 | 4.738144796 | 15.82881316 | 3.58E-38 | 8.88E-36 | 76.13330279 |
| HMMR | 1.372913556 | 1.327375566 | 15.78733466 | 4.87E-38 | 1.19E-35 | 75.82780425 |
| GNPAT | 1.147523644 | 4.302850679 | 15.78264155 | 5.04E-38 | 1.21E-35 | 75.79323471 |
| TRPV4 | -1.452100548 | 1.718552036 | -15.75290391 | 6.28E-38 | 1.49E-35 | 75.57416985 |
| NSMCE2 | 1.247499585 | 3.229411765 | 15.72235932 | 7.88E-38 | 1.83E-35 | 75.34913042 |
| CCNB1 | 1.932955865 | 2.053665158 | 15.70779775 | 8.79E-38 | 2.02E-35 | 75.24183647 |
| FAM65C | -1.60811183 | 1.530497738 | -15.68899694 | 1.01E-37 | 2.30E-35 | 75.10329668 |
| RNASEH2A | 1.314744483 | 2.57959276 | 15.64119931 | 1.44E-37 | 3.19E-35 | 74.75103579 |
| CLDN10 | -1.448109341 | 0.933936652 | -15.62741756 | 1.60E-37 | 3.44E-35 | 74.64945389 |
| SLC26A6 | 1.681297495 | 2.746561086 | 15.60805679 | 1.84E-37 | 3.94E-35 | 74.50674093 |
| BCO2 | -2.229001161 | 3.023710407 | -15.55072277 | 2.82E-37 | 5.97E-35 | 74.08405581 |
| SLC5A1 | -1.143537415 | 0.643755656 | -15.53543664 | 3.16E-37 | 6.63E-35 | 73.97134628 |
| NUSAP1 | 1.742620707 | 2.520180995 | 15.53386379 | 3.20E-37 | 6.64E-35 | 73.95974882 |
| TOP2A | 2.014513025 | 1.920995475 | 15.52417715 | 3.44E-37 | 7.07E-35 | 73.88832248 |
| TAF6 | 1.106037 | 3.195475113 | 15.5185896 | 3.59E-37 | 7.29E-35 | 73.8471205 |
| FAM83D | 1.76307118 | 1.765746606 | 15.49463293 | 4.28E-37 | 8.53E-35 | 73.67045741 |
| PRC1 | 1.715313589 | 1.912533937 | 15.47584957 | 4.93E-37 | 9.48E-35 | 73.53193352 |
| CDK1 | 1.74593413 | 1.836923077 | 15.44935462 | 6.00E-37 | 1.13E-34 | 73.33652329 |
| CDKN2C | 1.381657541 | 2.291221719 | 15.4492669 | 6.00E-37 | 1.13E-34 | 73.33587635 |
| IGFALS | -2.568712461 | 2.64361991 | -15.45153467 | 5.90E-37 | 1.13E-34 | 73.35260268 |
| DNASE1L3 | -2.513531608 | 4.514841629 | -15.43993365 | 6.44E-37 | 1.20E-34 | 73.26703599 |
| KIFC1 | 1.457186826 | 1.348054299 | 15.41524014 | 7.73E-37 | 1.41E-34 | 73.08489145 |
| ARPC1A | 1.072025884 | 5.028280543 | 15.35472376 | 1.21E-36 | 2.20E-34 | 72.63845159 |
| C7 | -3.501777003 | 4.358190045 | -15.33844514 | 1.37E-36 | 2.44E-34 | 72.5183479 |
| PRUNE | 1.084991704 | 2.820904977 | 15.32555818 | 1.51E-36 | 2.64E-34 | 72.42326399 |
| CENPF | 1.339531276 | 1.035837104 | 15.20462732 | 3.71E-36 | 6.19E-34 | 71.53084444 |
| CCDC34 | 1.286878215 | 1.98918552 | 15.18721434 | 4.22E-36 | 6.99E-34 | 71.40232274 |
| NEK2 | 1.53560146 | 1.186696833 | 15.16104359 | 5.13E-36 | 8.42E-34 | 71.20915265 |
| TCF21 | -1.199624191 | 1.104886878 | -15.08315913 | 9.15E-36 | 1.48E-33 | 70.63421703 |
| STMN1 | 1.731611083 | 4.547420814 | 15.06232638 | 1.07E-35 | 1.72E-33 | 70.48041803 |
| SLC25A39 | 1.20319479 | 5.468733032 | 14.96869424 | 2.15E-35 | 3.40E-33 | 69.78911341 |
| ZIC2 | 1.258392235 | 0.775882353 | 14.96585052 | 2.19E-35 | 3.44E-33 | 69.76811629 |
| EFNA4 | 1.218764725 | 2.18719457 | 14.91870434 | 3.11E-35 | 4.82E-33 | 69.41999439 |
| NECAB3 | 1.463404679 | 3.466923077 | 14.89153079 | 3.81E-35 | 5.86E-33 | 69.21934045 |
| AKR1C3 | 1.86717936 | 6.743076923 | 14.87260642 | 4.39E-35 | 6.65E-33 | 69.07959699 |
| NUF2 | 1.463870914 | 1.272488688 | 14.84839519 | 5.26E-35 | 7.80E-33 | 68.9008108 |
| CDCA8 | 1.336695703 | 1.241809955 | 14.791257 | 8.05E-35 | 1.15E-32 | 68.47886708 |
| UBE2C | 2.249198606 | 2.378959276 | 14.76258081 | 9.96E-35 | 1.40E-32 | 68.26710029 |
| HJURP | 1.472134561 | 1.249230769 | 14.75119243 | 1.08E-34 | 1.51E-32 | 68.18299933 |
| CDKN2A | 1.878592169 | 2.196877828 | 14.73437409 | 1.23E-34 | 1.69E-32 | 68.05879885 |
| PLOD3 | 1.09748963 | 4.148371041 | 14.72315128 | 1.34E-34 | 1.82E-32 | 67.97592019 |
| ADAMTSL2 | -2.17574747 | 2.622941176 | -14.72234744 | 1.34E-34 | 1.82E-32 | 67.96998396 |
| DIRAS3 | -1.242995686 | 1.260135747 | -14.70717996 | 1.50E-34 | 2.01E-32 | 67.85797443 |
| GPAA1 | 1.449755268 | 4.912081448 | 14.675545 | 1.90E-34 | 2.53E-32 | 67.62435508 |
| RABEPK | 1.368957193 | 3.983438914 | 14.66215967 | 2.10E-34 | 2.77E-32 | 67.52550675 |
| MELK | 1.372144516 | 1.251131222 | 14.56278991 | 4.41E-34 | 5.57E-32 | 66.79169214 |
| PBK | 1.372432388 | 1.165475113 | 14.53139275 | 5.57E-34 | 7.00E-32 | 66.55984262 |
| TMEM101 | 1.106545545 | 3.235656109 | 14.52066226 | 6.03E-34 | 7.49E-32 | 66.48060552 |
| EPHA2 | -1.525563298 | 2.779502262 | -14.49995618 | 7.04E-34 | 8.64E-32 | 66.32770807 |
| RNF187 | 1.159313921 | 4.359004525 | 14.47269547 | 8.63E-34 | 1.03E-31 | 66.12641471 |
| RND3 | -1.899770201 | 4.321131222 | -14.45950568 | 9.52E-34 | 1.13E-31 | 66.02902337 |
| TMEM106C | 1.442640617 | 4.348325792 | 14.45773298 | 9.64E-34 | 1.14E-31 | 66.01593416 |
| LYVE1 | -1.981786959 | 2.146606335 | -14.44212663 | 1.08E-33 | 1.26E-31 | 65.90070146 |
| PPOX | 1.086144848 | 2.767873303 | 14.4381977 | 1.12E-33 | 1.29E-31 | 65.87169179 |
| CDC20 | 1.915336818 | 1.728144796 | 14.43650907 | 1.13E-33 | 1.30E-31 | 65.85922366 |
| CFTR | -1.568251203 | 0.976968326 | -14.42374382 | 1.24E-33 | 1.42E-31 | 65.76497118 |
| CLN3 | 1.065803053 | 3.960633484 | 14.42131507 | 1.26E-33 | 1.44E-31 | 65.7470387 |
| EHMT2 | 1.188228804 | 2.852036199 | 14.38530889 | 1.65E-33 | 1.84E-31 | 65.48119648 |
| NBLA00301 | -1.198326697 | 0.916425339 | -14.33883085 | 2.34E-33 | 2.53E-31 | 65.13806018 |
| SSR2 | 1.082644765 | 6.631945701 | 14.33485083 | 2.41E-33 | 2.58E-31 | 65.10867797 |
| KIF20A | 1.350866932 | 1.193574661 | 14.30493798 | 3.01E-33 | 3.16E-31 | 64.88785504 |
| TOMM20 | 1.044783474 | 5.034751131 | 14.25544401 | 4.35E-33 | 4.53E-31 | 64.52250901 |
| SLC38A6 | 1.117996516 | 2.390090498 | 14.24072295 | 4.85E-33 | 5.03E-31 | 64.41385117 |
| ANLN | 1.345567446 | 1.114298643 | 14.22390301 | 5.50E-33 | 5.65E-31 | 64.28970565 |
| CDCA5 | 1.424009457 | 1.301221719 | 14.19084424 | 7.03E-33 | 7.02E-31 | 64.04571845 |
| RCAN1 | -1.426019579 | 4.757149321 | -14.18136307 | 7.55E-33 | 7.50E-31 | 63.97574726 |
| HAND2 | -1.433059565 | 1.215067873 | -14.17034197 | 8.19E-33 | 8.10E-31 | 63.89441358 |
| AADAT | -1.391519827 | 2.889411765 | -14.16771115 | 8.35E-33 | 8.15E-31 | 63.87499897 |
| OLFML3 | -1.78807118 | 2.385339367 | -14.150383 | 9.50E-33 | 9.10E-31 | 63.74712608 |
| ITLN1 | -1.388581384 | 1.128190045 | -14.14215756 | 1.01E-32 | 9.62E-31 | 63.68642863 |
| IGF2AS | -1.340725071 | 1.122171946 | -14.08896767 | 1.50E-32 | 1.37E-30 | 63.29396299 |
| SF3B4 | 1.031169736 | 4.216153846 | 14.06539281 | 1.79E-32 | 1.61E-30 | 63.12003426 |
| CDCA3 | 1.497450639 | 1.415565611 | 14.05596831 | 1.92E-32 | 1.71E-30 | 63.05050665 |
| MFSD2A | -3.022628173 | 3.814253394 | -14.04700183 | 2.05E-32 | 1.81E-30 | 62.98435987 |
| TROAP | 1.524249212 | 1.323031674 | 14.04335628 | 2.11E-32 | 1.85E-30 | 62.95746681 |
| SNRPC | 1.039624191 | 4.926063348 | 14.00758061 | 2.75E-32 | 2.39E-30 | 62.69356883 |
| UCK2 | 1.101687407 | 1.988054299 | 13.99938121 | 2.92E-32 | 2.51E-30 | 62.63309084 |
| MFAP4 | -2.770652895 | 3.123574661 | -13.9809531 | 3.35E-32 | 2.86E-30 | 62.49717338 |
| RRM2 | 1.688557325 | 1.999683258 | 13.97383566 | 3.53E-32 | 3.01E-30 | 62.44468079 |
| MSTO1 | 1.016728887 | 1.905565611 | 13.9615086 | 3.87E-32 | 3.26E-30 | 62.35376947 |
| NEU1 | 1.153242907 | 4.499095023 | 13.95301213 | 4.13E-32 | 3.46E-30 | 62.29111093 |
| FBP1 | -2.406832587 | 7.141266968 | -13.94949039 | 4.24E-32 | 3.53E-30 | 62.26513989 |
| INTS8 | 1.009120624 | 2.937963801 | 13.93446984 | 4.74E-32 | 3.94E-30 | 62.15437508 |
| DCN | -3.667795752 | 5.301402715 | -13.91596916 | 5.43E-32 | 4.48E-30 | 62.01795589 |
| HSD17B13 | -3.700717604 | 4.971855204 | -13.91276203 | 5.57E-32 | 4.57E-30 | 61.99430841 |
| INMT | -1.945101211 | 2.317330317 | -13.90607467 | 5.85E-32 | 4.79E-30 | 61.94500069 |
| CYP39A1 | -1.898910735 | 3.090180995 | -13.89753071 | 6.23E-32 | 5.08E-30 | 61.88200578 |
| C1QTNF1 | -1.977424092 | 2.760769231 | -13.8927127 | 6.46E-32 | 5.25E-30 | 61.84648333 |
| C15orf23 | 1.090244732 | 2.47158371 | 13.88064824 | 7.07E-32 | 5.63E-30 | 61.75753712 |
| AP1S1 | 1.035925004 | 4.309004525 | 13.87672208 | 7.28E-32 | 5.76E-30 | 61.72859221 |
| SPC25 | 1.007726896 | 1.040045249 | 13.86983756 | 7.66E-32 | 6.04E-30 | 61.67783838 |
| SNRPE | 1.172860461 | 4.359095023 | 13.86760103 | 7.79E-32 | 6.11E-30 | 61.66135066 |
| COMMD4 | 1.109624191 | 5.185022624 | 13.82431374 | 1.07E-31 | 8.28E-30 | 61.34226761 |
| SCNM1 | 1.052775842 | 2.863076923 | 13.77476621 | 1.55E-31 | 1.18E-29 | 60.9771155 |
| ALG1L | 1.616032023 | 1.500542986 | 13.7704693 | 1.60E-31 | 1.21E-29 | 60.94545241 |
| CCNE2 | 1.035833748 | 1.281402715 | 13.76858466 | 1.63E-31 | 1.23E-29 | 60.93156506 |
| CTSA | 1.074357889 | 6.110904977 | 13.75677594 | 1.77E-31 | 1.33E-29 | 60.84455319 |
| EPRS | 1.010454621 | 3.730542986 | 13.75081623 | 1.85E-31 | 1.39E-29 | 60.80064134 |
| POLR2K | 1.050074664 | 5.136063348 | 13.73993529 | 2.01E-31 | 1.48E-29 | 60.72047259 |
| C1orf31 | 1.137144516 | 4.435339367 | 13.73478662 | 2.09E-31 | 1.52E-29 | 60.6825397 |
| FCGR2B | -1.770631326 | 2.234841629 | -13.73576501 | 2.07E-31 | 1.52E-29 | 60.68974788 |
| ASS1 | -1.822478845 | 8.395475113 | -13.7297201 | 2.17E-31 | 1.58E-29 | 60.64521291 |
| FEN1 | 1.191523146 | 2.518461538 | 13.72558439 | 2.24E-31 | 1.61E-29 | 60.61474454 |
| CENPW | 1.509061722 | 2.375701357 | 13.7260499 | 2.23E-31 | 1.61E-29 | 60.61817397 |
| CDKN2AIPNL | 1.080397378 | 2.938552036 | 13.70480265 | 2.61E-31 | 1.87E-29 | 60.46165194 |
| NDC80 | 1.27937697 | 1.470316742 | 13.69984561 | 2.71E-31 | 1.93E-29 | 60.42513736 |
| BIRC5 | 1.639797578 | 1.542443439 | 13.68641362 | 2.99E-31 | 2.10E-29 | 60.32619943 |
| CCNB2 | 1.583408827 | 1.487692308 | 13.67192914 | 3.33E-31 | 2.32E-29 | 60.21951699 |
| TCF19 | 1.232020076 | 1.663755656 | 13.6700974 | 3.38E-31 | 2.34E-29 | 60.20602622 |
| CYP1A2 | -3.301302472 | 3.263031674 | -13.6670463 | 3.46E-31 | 2.39E-29 | 60.18355529 |
| CDC25C | 1.132831425 | 0.898959276 | 13.64103368 | 4.19E-31 | 2.89E-29 | 59.99199081 |
| MLEC | 1.13109922 | 4.194117647 | 13.62906262 | 4.58E-31 | 3.15E-29 | 59.90384172 |
| TUBG1 | 1.028762237 | 2.987058824 | 13.62253315 | 4.81E-31 | 3.29E-29 | 59.85576436 |
| C1orf85 | 1.359077485 | 5.013348416 | 13.61100631 | 5.24E-31 | 3.55E-29 | 59.77089511 |
| PLVAP | 1.860345114 | 3.770497738 | 13.60739977 | 5.38E-31 | 3.64E-29 | 59.74434214 |
| TXNRD1 | 1.808310105 | 4.707149321 | 13.59040704 | 6.11E-31 | 4.08E-29 | 59.61924183 |
| ID1 | -2.34163846 | 4.68479638 | -13.57755373 | 6.72E-31 | 4.42E-29 | 59.5246241 |
| ESM1 | 1.742004314 | 1.346470588 | 13.5513612 | 8.16E-31 | 5.22E-29 | 59.33183425 |
| DARS2 | 1.029922018 | 2.60239819 | 13.54874733 | 8.32E-31 | 5.30E-29 | 59.31259649 |
| NGFR | -1.61690891 | 1.969683258 | -13.5360503 | 9.14E-31 | 5.81E-29 | 59.21915267 |
| LCAT | -1.846285051 | 5.018552036 | -13.52888844 | 9.64E-31 | 6.09E-29 | 59.16644816 |
| SRXN1 | 1.847937614 | 4.475837104 | 13.52207469 | 1.01E-30 | 6.37E-29 | 59.11630763 |
| MT1F | -3.288885017 | 5.155248869 | -13.51660541 | 1.06E-30 | 6.60E-29 | 59.07606226 |
| GHR | -1.797501244 | 3.824886878 | -13.50433208 | 1.16E-30 | 7.20E-29 | 58.98575463 |
| GPR182 | -1.022899452 | 0.818959276 | -13.48958835 | 1.29E-30 | 7.99E-29 | 58.87727909 |
| MRPL24 | 1.126541397 | 5.94280543 | 13.48757436 | 1.31E-30 | 8.06E-29 | 58.86246218 |
| GM2A | 1.085158454 | 3.524977376 | 13.48757605 | 1.31E-30 | 8.06E-29 | 58.8624746 |
| UBE2S | 1.372388419 | 3.081266968 | 13.45960812 | 1.61E-30 | 9.78E-29 | 58.65673467 |
| MND1 | 1.080947403 | 1.218552036 | 13.45848026 | 1.63E-30 | 9.84E-29 | 58.6484386 |
| PFDN6 | 1.11690808 | 5.457239819 | 13.43746192 | 1.90E-30 | 1.13E-28 | 58.49384803 |
| BUB1 | 1.204484818 | 1.107104072 | 13.42999687 | 2.01E-30 | 1.18E-28 | 58.43894765 |
| PIR | 1.43110005 | 3.475882353 | 13.42657283 | 2.06E-30 | 1.21E-28 | 58.41376708 |
| ATP6V1C1 | 1.147668824 | 3.392217195 | 13.41587291 | 2.23E-30 | 1.30E-28 | 58.33508317 |
| APOA1BP | 1.002366849 | 5.290633484 | 13.40103397 | 2.49E-30 | 1.45E-28 | 58.22597179 |
| MKI67 | 1.217867098 | 1.147511312 | 13.39485446 | 2.61E-30 | 1.51E-28 | 58.18053697 |
| DTL | 1.066441015 | 1.059049774 | 13.36686534 | 3.21E-30 | 1.84E-28 | 57.97477179 |
| TRPV6 | -1.153660196 | 0.708325792 | -13.3632812 | 3.29E-30 | 1.88E-28 | 57.94842555 |
| EZH2 | 1.199174548 | 2.335475113 | 13.32971179 | 4.22E-30 | 2.37E-28 | 57.70169767 |
| ROBO1 | 1.933182346 | 2.403076923 | 13.32020806 | 4.53E-30 | 2.52E-28 | 57.63185844 |
| BCAP31 | 1.028904928 | 6.542036199 | 13.29509355 | 5.46E-30 | 3.00E-28 | 57.44732567 |
| LRRC14 | 1.001159781 | 2.331900452 | 13.29375446 | 5.51E-30 | 3.02E-28 | 57.43748749 |
| RRAGD | 1.200781483 | 1.986063348 | 13.28872193 | 5.72E-30 | 3.12E-28 | 57.40051482 |
| MCM7 | 1.099410154 | 4.10199095 | 13.28556914 | 5.86E-30 | 3.18E-28 | 57.37735282 |
| CKAP2 | 1.082723577 | 1.67760181 | 13.2623807 | 6.96E-30 | 3.77E-28 | 57.20701628 |
| RBM24 | 1.390242243 | 1.174162896 | 13.25344002 | 7.43E-30 | 4.02E-28 | 57.1413485 |
| TLX1 | 1.548813672 | 1.253846154 | 13.24600562 | 7.85E-30 | 4.23E-28 | 57.08674769 |
| PLGLA | -1.537484652 | 1.895520362 | -13.2353358 | 8.50E-30 | 4.55E-28 | 57.00839039 |
| MAFG | 1.012465572 | 2.426968326 | 13.23337331 | 8.62E-30 | 4.61E-28 | 56.99397899 |
| LOC344887 | 2.130267131 | 1.369909502 | 13.22412797 | 9.23E-30 | 4.88E-28 | 56.92608933 |
| C8orf33 | 1.213597976 | 3.395339367 | 13.21068569 | 1.02E-29 | 5.38E-28 | 56.8273902 |
| CD5L | -2.647774183 | 3.453800905 | -13.20147108 | 1.09E-29 | 5.73E-28 | 56.75973873 |
| TTK | 1.081190476 | 0.953891403 | 13.18544036 | 1.23E-29 | 6.38E-28 | 56.64205728 |
| GMNN | 1.439126431 | 3.880859729 | 13.17938725 | 1.29E-29 | 6.64E-28 | 56.59762554 |
| KIF2C | 1.244222665 | 1.163710407 | 13.1654719 | 1.43E-29 | 7.27E-28 | 56.49549098 |
| TARBP1 | 1.16497926 | 2.803484163 | 13.16223734 | 1.46E-29 | 7.43E-28 | 56.47175198 |
| SAE1 | 1.023005641 | 3.814977376 | 13.11210622 | 2.12E-29 | 1.05E-27 | 56.10391555 |
| PRSS22 | -1.153295172 | 0.688733032 | -13.08916248 | 2.51E-29 | 1.23E-27 | 55.93562011 |
| IRAK1 | 1.151114983 | 3.78638009 | 13.05850824 | 3.15E-29 | 1.51E-27 | 55.71082122 |
| KIF4A | 1.096217023 | 0.960723982 | 13.00767968 | 4.58E-29 | 2.16E-27 | 55.33821655 |
| TP53I3 | 1.823261988 | 3.482307692 | 12.98867575 | 5.27E-29 | 2.45E-27 | 55.19895193 |
| RDBP | 1.170406504 | 4.331402715 | 12.97821724 | 5.70E-29 | 2.63E-27 | 55.1223207 |
| PSPH | 1.184800066 | 2.298597285 | 12.94046378 | 7.53E-29 | 3.41E-27 | 54.84576009 |
| TMEM9 | 1.051344782 | 4.749321267 | 12.86254362 | 1.34E-28 | 5.90E-27 | 54.27529235 |
| NAT2 | -2.176723079 | 3.08239819 | -12.85140622 | 1.45E-28 | 6.38E-27 | 54.19379086 |
| E2F1 | 1.404535424 | 1.480995475 | 12.84612103 | 1.51E-28 | 6.60E-27 | 54.15511816 |
| KRTCAP2 | 1.149617554 | 6.728914027 | 12.84135282 | 1.57E-28 | 6.82E-27 | 54.12023016 |
| SHBG | -2.413747304 | 3.983438914 | -12.83558844 | 1.63E-28 | 7.09E-27 | 54.0780558 |
| RFC4 | 1.134144682 | 2.721628959 | 12.79973149 | 2.13E-28 | 9.13E-27 | 53.81577127 |
| ZNF238 | 1.169827443 | 3.333529412 | 12.79848833 | 2.15E-28 | 9.20E-27 | 53.80667966 |
| SLC50A1 | 1.157578397 | 4.804977376 | 12.78751081 | 2.33E-28 | 9.85E-27 | 53.72640335 |
| CYP2B6 | -3.027049942 | 6.139547511 | -12.78022806 | 2.46E-28 | 1.03E-26 | 53.67315151 |
| AURKB | 1.426006305 | 1.486108597 | 12.77094436 | 2.63E-28 | 1.10E-26 | 53.60527484 |
| IQGAP3 | 1.190926663 | 1.519864253 | 12.76493728 | 2.75E-28 | 1.14E-26 | 53.56135844 |
| SH3YL1 | -1.522046624 | 2.851357466 | -12.75949884 | 2.86E-28 | 1.19E-26 | 53.52160185 |
| SMG5 | 1.002880372 | 4.14959276 | 12.75359493 | 2.99E-28 | 1.23E-26 | 53.47844523 |
| TFPI2 | -1.675735855 | 1.768371041 | -12.75123081 | 3.04E-28 | 1.25E-26 | 53.46116474 |
| DLGAP5 | 1.069012776 | 0.927828054 | 12.73407323 | 3.45E-28 | 1.40E-26 | 53.33576527 |
| JMJD5 | -1.812562635 | 2.856606335 | -12.73279871 | 3.49E-28 | 1.41E-26 | 53.32645117 |
| LPPR1 | 1.495069686 | 2.219954751 | 12.72188782 | 3.78E-28 | 1.52E-26 | 53.24672074 |
| ASPG | -2.478310105 | 3.099954751 | -12.71626934 | 3.94E-28 | 1.58E-26 | 53.20566806 |
| MDK | 2.690111996 | 4.974660633 | 12.71490636 | 3.98E-28 | 1.59E-26 | 53.19570954 |
| DTYMK | 1.229531276 | 3.104615385 | 12.70696104 | 4.22E-28 | 1.68E-26 | 53.13766066 |
| FAM163B | -1.269101543 | 1.037647059 | -12.7042345 | 4.30E-28 | 1.71E-26 | 53.11774169 |
| SLC25A47 | -3.345460428 | 5.471312217 | -12.69593524 | 4.58E-28 | 1.81E-26 | 53.05711454 |
| ADRA1A | -1.647272275 | 2.112171946 | -12.67884461 | 5.19E-28 | 2.03E-26 | 52.93228384 |
| APOF | -2.685834578 | 4.985475113 | -12.67925814 | 5.17E-28 | 2.03E-26 | 52.93530399 |
| MT2A | -2.446475859 | 9.221447964 | -12.67207106 | 5.46E-28 | 2.13E-26 | 52.88281646 |
| BAG2 | 1.04153559 | 2.756923077 | 12.64418422 | 6.70E-28 | 2.59E-26 | 52.67920009 |
| FOXM1 | 1.341969471 | 2.08239819 | 12.62492938 | 7.72E-28 | 2.93E-26 | 52.53865015 |
| ENAH | 1.149235938 | 1.982579186 | 12.59684623 | 9.49E-28 | 3.58E-26 | 52.33371734 |
| C19orf77 | -3.072784138 | 2.994705882 | -12.58644806 | 1.02E-27 | 3.86E-26 | 52.25785617 |
| C20orf27 | 1.153770533 | 3.16959276 | 12.57738668 | 1.10E-27 | 4.08E-26 | 52.19175569 |
| MSL3P1 | 1.118554007 | 1.523257919 | 12.54438094 | 1.40E-27 | 5.16E-26 | 51.95105058 |
| CENPA | 1.081987722 | 0.86321267 | 12.52704553 | 1.59E-27 | 5.82E-26 | 51.82466676 |
| HES6 | 1.22062303 | 1.651085973 | 12.52623942 | 1.60E-27 | 5.85E-26 | 51.81879056 |
| GGT5 | -1.709616725 | 2.884208145 | -12.51850127 | 1.69E-27 | 6.18E-26 | 51.76238519 |
| CYHR1 | 1.12639041 | 3.639864253 | 12.5153114 | 1.73E-27 | 6.32E-26 | 51.73913508 |
| ANXA3 | -1.243445329 | 1.200497738 | -12.49953393 | 1.94E-27 | 7.03E-26 | 51.62415143 |
| PRSS8 | -1.92400448 | 2.159276018 | -12.49576816 | 2.00E-27 | 7.18E-26 | 51.59671059 |
| ECT2 | 1.107646424 | 1.470452489 | 12.49218836 | 2.05E-27 | 7.36E-26 | 51.57062617 |
| NCAPG | 1.116221171 | 1.485837104 | 12.48227313 | 2.21E-27 | 7.86E-26 | 51.4983846 |
| NME1 | 1.18363033 | 5.505701357 | 12.48234219 | 2.20E-27 | 7.86E-26 | 51.49888779 |
| BUB1B | 1.172254024 | 1.042941176 | 12.47789912 | 2.28E-27 | 8.08E-26 | 51.46651899 |
| MCM3 | 1.089053426 | 3.4239819 | 12.47811648 | 2.27E-27 | 8.08E-26 | 51.46810245 |
| SHARPIN | 1.127258172 | 4.216877828 | 12.45806236 | 2.63E-27 | 9.21E-26 | 51.32202672 |
| TALDO1 | 1.014472374 | 6.109411765 | 12.43774243 | 3.06E-27 | 1.06E-25 | 51.17405474 |
| PYCRL | 1.067110503 | 2.239728507 | 12.43035814 | 3.23E-27 | 1.11E-25 | 51.12029162 |
| SAC3D1 | 1.108967977 | 2.191085973 | 12.41455314 | 3.63E-27 | 1.24E-25 | 51.00523745 |
| TSLP | -1.078115148 | 1.386289593 | -12.41175565 | 3.70E-27 | 1.26E-25 | 50.98487544 |
| ASF1B | 1.080832089 | 1.291855204 | 12.38932808 | 4.36E-27 | 1.48E-25 | 50.82166057 |
| CAMK2B | -1.106205409 | 0.950859729 | -12.37957937 | 4.69E-27 | 1.58E-25 | 50.75073098 |
| TRIM16L | 1.826809358 | 2.061628959 | 12.37751725 | 4.76E-27 | 1.60E-25 | 50.7357286 |
| GGH | 1.521588684 | 5.911040724 | 12.37299818 | 4.92E-27 | 1.66E-25 | 50.702853 |
| HDAC11 | 1.038861789 | 1.838190045 | 12.37093563 | 5.00E-27 | 1.68E-25 | 50.68784892 |
| ASPA | -1.06541397 | 1.668868778 | -12.33033348 | 6.73E-27 | 2.22E-25 | 50.39257617 |
| MRPS21 | 1.099355401 | 6.295429864 | 12.31822887 | 7.35E-27 | 2.42E-25 | 50.30458034 |
| C1orf35 | 1.066611913 | 3.127104072 | 12.28668586 | 9.27E-27 | 3.01E-25 | 50.07534712 |
| KIF23 | 1.086034511 | 1.099954751 | 12.2756661 | 1.00E-26 | 3.25E-25 | 49.99528789 |
| GPC3 | 3.683977103 | 4.84199095 | 12.26450371 | 1.09E-26 | 3.51E-25 | 49.91420568 |
| GABRP | -1.073836901 | 0.681221719 | -12.25795707 | 1.14E-26 | 3.67E-25 | 49.86665791 |
| BDH2 | -1.062816492 | 3.856334842 | -12.24469932 | 1.26E-26 | 4.01E-25 | 49.7703821 |
| CCNA2 | 1.11991787 | 1.928506787 | 12.23434596 | 1.36E-26 | 4.30E-25 | 49.69521072 |
| STEAP4 | -1.740287042 | 2.312036199 | -12.23345097 | 1.37E-26 | 4.33E-25 | 49.68871317 |
| FAM83H | 1.255854488 | 3.003755656 | 12.23081172 | 1.40E-26 | 4.39E-25 | 49.66955284 |
| ANGPTL1 | -1.186527294 | 1.505339367 | -12.23012158 | 1.40E-26 | 4.41E-25 | 49.66454273 |
| TUBE1 | -1.054846524 | 3.365158371 | -12.20338737 | 1.71E-26 | 5.31E-25 | 49.47050384 |
| MAD2L1 | 1.256765389 | 1.680180995 | 12.19105253 | 1.87E-26 | 5.77E-25 | 49.38100328 |
| LSM4 | 1.162108014 | 5.18321267 | 12.18934125 | 1.89E-26 | 5.84E-25 | 49.36858776 |
| DLG5 | 1.149748631 | 1.956742081 | 12.18493497 | 1.95E-26 | 6.02E-25 | 49.33662112 |
| HAMP | -4.394116476 | 4.927058824 | -12.13492003 | 2.82E-26 | 8.53E-25 | 48.97392691 |
| C1R | -1.375288701 | 8.560588235 | -12.13001703 | 2.92E-26 | 8.83E-25 | 48.93838709 |
| TTC36 | -2.883528289 | 4.298099548 | -12.11443427 | 3.27E-26 | 9.84E-25 | 48.82545239 |
| CD4 | -1.491130745 | 4.303257919 | -12.08223568 | 4.14E-26 | 1.24E-24 | 48.59218543 |
| MOGAT2 | -1.808550689 | 2.491900452 | -12.07987534 | 4.21E-26 | 1.25E-24 | 48.5750904 |
| NME1-NME2 | 1.048574747 | 7.024208145 | 12.04702357 | 5.36E-26 | 1.59E-24 | 48.33722628 |
| C9 | -3.616797743 | 5.979276018 | -12.03157228 | 6.00E-26 | 1.76E-24 | 48.22539533 |
| KLHDC3 | 1.005240584 | 4.925294118 | 12.0293603 | 6.09E-26 | 1.79E-24 | 48.2093882 |
| TIMD4 | -1.580610586 | 1.450497738 | -12.013548 | 6.84E-26 | 1.99E-24 | 48.09497838 |
| CCL14 | -1.642610752 | 6.013031674 | -12.0023075 | 7.42E-26 | 2.14E-24 | 48.01366634 |
| SNRPB | 1.065882695 | 5.461085973 | 11.98377184 | 8.50E-26 | 2.44E-24 | 47.87961595 |
| A2M | -1.973010619 | 8.587511312 | -11.98098948 | 8.67E-26 | 2.48E-24 | 47.85949742 |
| AQP3 | -1.41778082 | 4.51 | -11.97230616 | 9.24E-26 | 2.62E-24 | 47.79671695 |
| SCRIB | 1.127526132 | 3.226108597 | 11.958071 | 1.03E-25 | 2.88E-24 | 47.69381683 |
| TKT | 1.657339472 | 5.391900452 | 11.95288036 | 1.06E-25 | 2.98E-24 | 47.65630211 |
| SQSTM1 | 1.178656048 | 7.656606335 | 11.95166921 | 1.07E-25 | 3.00E-24 | 47.64754919 |
| CYP2C8 | -3.047107184 | 7.747466063 | -11.90942227 | 1.46E-25 | 4.00E-24 | 47.34234693 |
| EDNRB | -1.280339306 | 2.935882353 | -11.88634 | 1.73E-25 | 4.67E-24 | 47.17569076 |
| CCHCR1 | 1.074396051 | 2.641538462 | 11.88595816 | 1.73E-25 | 4.68E-24 | 47.17293444 |
| SERPINB8 | -1.334131409 | 2.503800905 | -11.8779818 | 1.84E-25 | 4.93E-24 | 47.11536065 |
| TXN | 1.158081135 | 8.37158371 | 11.85083669 | 2.24E-25 | 5.96E-24 | 46.91948777 |
| HOXA13 | 1.190059731 | 0.717647059 | 11.8503455 | 2.25E-25 | 5.97E-24 | 46.91594438 |
| TGM3 | 2.128429567 | 1.445927602 | 11.83476318 | 2.52E-25 | 6.64E-24 | 46.80355043 |
| MT1M | -3.028654389 | 4.263348416 | -11.82720794 | 2.66E-25 | 7.01E-24 | 46.74906663 |
| NR2C2AP | 1.185906753 | 3.286968326 | 11.82340775 | 2.73E-25 | 7.19E-24 | 46.72166478 |
| CLDN15 | 1.631326531 | 3.336606335 | 11.81038771 | 3.01E-25 | 7.90E-24 | 46.62779649 |
| ATAD2 | 1.294598474 | 2.744298643 | 11.77503195 | 3.89E-25 | 1.01E-23 | 46.37301256 |
| PLK1 | 1.145967314 | 1.344841629 | 11.77339421 | 3.94E-25 | 1.02E-23 | 46.36121459 |
| TP53BP2 | 1.059214369 | 3.457782805 | 11.75774274 | 4.41E-25 | 1.13E-23 | 46.24848281 |
| KRT19 | -2.081511531 | 1.533348416 | -11.73307038 | 5.28E-25 | 1.34E-23 | 46.07084488 |
| PDZK1 | 1.071637631 | 4.563574661 | 11.71714914 | 5.92E-25 | 1.49E-23 | 45.95625837 |
| SERPINA11 | -2.175326033 | 6.199502262 | -11.70043147 | 6.69E-25 | 1.67E-23 | 45.83597773 |
| FAM19A5 | -1.02792434 | 0.787285068 | -11.67185706 | 8.23E-25 | 2.03E-23 | 45.63048052 |
| PPP1R16A | 1.167184337 | 4.182262443 | 11.66224329 | 8.82E-25 | 2.17E-23 | 45.56136758 |
| PHLDA1 | -1.591427742 | 3.960497738 | -11.65734806 | 9.14E-25 | 2.24E-23 | 45.52618098 |
| SPINT2 | -1.93292517 | 2.603800905 | -11.6500714 | 9.64E-25 | 2.36E-23 | 45.47388319 |
| ALB | -1.776580388 | 14.44 | -11.61129698 | 1.28E-24 | 3.07E-23 | 45.19533704 |
| HAO2 | -2.586170566 | 4.558597285 | -11.61049319 | 1.28E-24 | 3.08E-23 | 45.1895651 |
| SMYD2 | 1.111669155 | 4.538099548 | 11.60770078 | 1.31E-24 | 3.13E-23 | 45.16951375 |
| DBNDD1 | 1.597077319 | 2.502850679 | 11.59847051 | 1.40E-24 | 3.33E-23 | 45.10324251 |
| NME2 | 1.010155135 | 7.509049774 | 11.59512069 | 1.44E-24 | 3.39E-23 | 45.07919464 |
| PGLYRP2 | -2.329117305 | 5.206561086 | -11.57397079 | 1.67E-24 | 3.91E-23 | 44.92740053 |
| COLEC11 | -1.868164925 | 4.898823529 | -11.56276614 | 1.81E-24 | 4.23E-23 | 44.84701072 |
| KPNA2 | 1.359243405 | 3.802624434 | 11.54862733 | 2.01E-24 | 4.64E-23 | 44.74559583 |
| FDPS | 1.267574249 | 6.362217195 | 11.54801194 | 2.02E-24 | 4.65E-23 | 44.74118241 |
| ZNF323 | 1.255887672 | 1.90561086 | 11.53933598 | 2.15E-24 | 4.93E-23 | 44.67896696 |
| EHD3 | -1.040410652 | 1.486968326 | -11.53962188 | 2.15E-24 | 4.93E-23 | 44.68101701 |
| BCAT2 | 1.319258337 | 3.076696833 | 11.531423 | 2.28E-24 | 5.20E-23 | 44.62223267 |
| TK1 | 1.363354903 | 2.543484163 | 11.52431532 | 2.40E-24 | 5.46E-23 | 44.57128018 |
| CDT1 | 1.089073337 | 1.089095023 | 11.52054842 | 2.46E-24 | 5.60E-23 | 44.54427965 |
| CIDEB | -1.098417123 | 5.619276018 | -11.51462941 | 2.57E-24 | 5.82E-23 | 44.50185747 |
| PDGFRA | -1.605111996 | 1.934208145 | -11.50081651 | 2.84E-24 | 6.40E-23 | 44.40287959 |
| FASTK | 1.045282064 | 4.807375566 | 11.49615573 | 2.94E-24 | 6.61E-23 | 44.36948873 |
| UGT2B11 | 2.120143521 | 2.529683258 | 11.48104369 | 3.28E-24 | 7.35E-23 | 44.26124555 |
| GBA3 | -2.108503401 | 3.998325792 | -11.4605363 | 3.80E-24 | 8.45E-23 | 44.11441303 |
| MT1E | -2.834785963 | 7.174615385 | -11.4460852 | 4.22E-24 | 9.32E-23 | 44.01098236 |
| ESR1 | -1.094223494 | 1.514977376 | -11.44117966 | 4.37E-24 | 9.65E-23 | 43.97587931 |
| SMYD3 | 1.160692716 | 2.290588235 | 11.43656522 | 4.52E-24 | 9.95E-23 | 43.9428628 |
| IGJ | -2.87829849 | 3.994479638 | -11.43231637 | 4.66E-24 | 1.02E-22 | 43.91246501 |
| AKR1B10 | 4.092607433 | 5.371357466 | 11.41342792 | 5.34E-24 | 1.16E-22 | 43.77736442 |
| LRRC45 | 1.146222831 | 2.732126697 | 11.40632331 | 5.62E-24 | 1.22E-22 | 43.72656284 |
| PLSCR4 | -1.102495437 | 3.120271493 | -11.40634608 | 5.62E-24 | 1.22E-22 | 43.7267256 |
| CD1D | -1.054753609 | 1.792352941 | -11.36929609 | 7.35E-24 | 1.56E-22 | 43.46192856 |
| ABCB6 | 1.128131741 | 3.297466063 | 11.36010722 | 7.85E-24 | 1.66E-22 | 43.39628937 |
| MT1G | -3.521616061 | 7.107556561 | -11.35310976 | 8.26E-24 | 1.74E-22 | 43.3463132 |
| KIAA0101 | 1.260861125 | 1.539909502 | 11.34767158 | 8.59E-24 | 1.81E-22 | 43.30747899 |
| CCL21 | -2.816116642 | 3.812352941 | -11.33544302 | 9.38E-24 | 1.97E-22 | 43.22017181 |
| MIR3658 | 1.322215862 | 3.575791855 | 11.33317215 | 9.53E-24 | 1.99E-22 | 43.20396129 |
| UROC1 | -2.29755102 | 3.808823529 | -11.27792861 | 1.42E-23 | 2.92E-22 | 42.80986807 |
| ACADS | -1.038726564 | 4.84361991 | -11.27256026 | 1.48E-23 | 3.02E-22 | 42.77159848 |
| TRIP13 | 1.04373403 | 0.977330317 | 11.25699487 | 1.65E-23 | 3.34E-22 | 42.66066368 |
| SMO | 1.285688568 | 3.251176471 | 11.25060301 | 1.73E-23 | 3.48E-22 | 42.6151205 |
| CCL19 | -2.884991704 | 3.467285068 | -11.24016722 | 1.86E-23 | 3.74E-22 | 42.54077836 |
| TRIM55 | 2.258038825 | 2.60918552 | 11.19605636 | 2.56E-23 | 5.06E-22 | 42.22674562 |
| HSP90AB1 | 1.07043471 | 7.778823529 | 11.1945353 | 2.59E-23 | 5.10E-22 | 42.21592286 |
| TMEM14A | 1.054737017 | 4.651719457 | 11.19075282 | 2.66E-23 | 5.22E-22 | 42.18901116 |
| HTATIP2 | 1.018313423 | 5.426651584 | 11.17742229 | 2.92E-23 | 5.70E-22 | 42.09418616 |
| CYP2A7 | -3.059537913 | 3.768506787 | -11.15923091 | 3.33E-23 | 6.47E-22 | 41.96483351 |
| TLCD1 | 1.13987556 | 2.585022624 | 11.15335198 | 3.48E-23 | 6.73E-22 | 41.92304267 |
| CLRN3 | -2.080624689 | 2.680678733 | -11.14934872 | 3.58E-23 | 6.91E-22 | 41.89458865 |
| SPINK1 | 4.464073337 | 4.202488688 | 11.12843432 | 4.16E-23 | 7.91E-22 | 41.74598046 |
| TPX2 | 1.248067861 | 2.054932127 | 11.11857565 | 4.46E-23 | 8.44E-22 | 41.67595577 |
| CENPM | 1.272612411 | 1.390633484 | 11.08510534 | 5.67E-23 | 1.06E-21 | 41.43834907 |
| RFXANK | 1.027067364 | 3.538054299 | 11.07591814 | 6.06E-23 | 1.13E-21 | 41.37316372 |
| NUDT1 | 1.074873071 | 2.579457014 | 11.07297575 | 6.19E-23 | 1.15E-21 | 41.35228988 |
| PRIM1 | 1.020515182 | 2.026244344 | 11.04856818 | 7.37E-23 | 1.34E-21 | 41.1791986 |
| SMPX | 1.883723245 | 1.332488688 | 11.0257241 | 8.68E-23 | 1.56E-21 | 41.0172925 |
| HGF | -1.382273934 | 1.765067873 | -11.01922335 | 9.09E-23 | 1.63E-21 | 40.97123608 |
| MANEAL | 1.009820806 | 2.256108597 | 11.01802162 | 9.17E-23 | 1.64E-21 | 40.96272297 |
| IMPDH2 | 1.029966816 | 4.871402715 | 11.00669389 | 9.94E-23 | 1.77E-21 | 40.88248938 |
| RASGEF1B | -1.186756263 | 3.161538462 | -10.98735146 | 1.14E-22 | 2.02E-21 | 40.7455427 |
| PIK3R2 | 1.116052763 | 2.852171946 | 10.95092987 | 1.48E-22 | 2.57E-21 | 40.48786127 |
| MLF1IP | 1.002159449 | 2.151538462 | 10.93854767 | 1.62E-22 | 2.79E-21 | 40.40031394 |
| DUSP23 | 1.039409325 | 5.383800905 | 10.93245431 | 1.69E-22 | 2.91E-21 | 40.35724195 |
| CDC6 | 1.099754438 | 1.292488688 | 10.91903979 | 1.86E-22 | 3.19E-21 | 40.26244357 |
| FEZ1 | -1.019463249 | 1.735565611 | -10.91242756 | 1.95E-22 | 3.33E-21 | 40.21572849 |
| GCH1 | -1.038909076 | 4.41841629 | -10.91185828 | 1.96E-22 | 3.34E-21 | 40.21170692 |
| GRAMD1C | -1.109163763 | 2.526968326 | -10.89160006 | 2.26E-22 | 3.81E-21 | 40.0686374 |
| DTX1 | -1.66944002 | 2.518099548 | -10.89181891 | 2.26E-22 | 3.81E-21 | 40.07018256 |
| TRIM16 | 1.454597644 | 1.648552036 | 10.87130488 | 2.61E-22 | 4.37E-21 | 39.92538542 |
| SDCBP2 | -1.626458437 | 2.678552036 | -10.87060457 | 2.63E-22 | 4.38E-21 | 39.92044372 |
| LAMC1 | 1.126309109 | 3.249819005 | 10.86450684 | 2.74E-22 | 4.55E-21 | 39.87741964 |
| CCDC3 | -1.404882197 | 2.504117647 | -10.85725003 | 2.89E-22 | 4.78E-21 | 39.82622671 |
| C8orf4 | -1.672064875 | 5.142352941 | -10.81509683 | 3.90E-22 | 6.33E-21 | 39.52906023 |
| MT1X | -2.51149328 | 7.529547511 | -10.80315633 | 4.25E-22 | 6.86E-21 | 39.44494662 |
| TDO2 | -2.261002987 | 6.793348416 | -10.79328871 | 4.56E-22 | 7.32E-21 | 39.37545643 |
| MYBL2 | 1.514424258 | 1.439502262 | 10.7627542 | 5.66E-22 | 8.95E-21 | 39.16054697 |
| C17orf90 | 1.0544815 | 3.384434389 | 10.73251831 | 7.02E-22 | 1.09E-20 | 38.94792272 |
| MSH5 | 1.056958686 | 2.436425339 | 10.7336042 | 6.96E-22 | 1.09E-20 | 38.95555572 |
| IL13RA2 | -1.117908578 | 1.078325792 | -10.72605564 | 7.35E-22 | 1.14E-20 | 38.90249988 |
| H2AFX | 1.148831093 | 3.041945701 | 10.68604979 | 9.76E-22 | 1.49E-20 | 38.62150774 |
| CRNDE | 1.286953708 | 1.335656109 | 10.671943 | 1.08E-21 | 1.64E-20 | 38.52250279 |
| PSMG3 | 1.01898374 | 3.952126697 | 10.66034878 | 1.17E-21 | 1.77E-20 | 38.4411623 |
| NDUFA4L2 | 1.353459433 | 2.252262443 | 10.65913638 | 1.18E-21 | 1.78E-20 | 38.4326582 |
| C19orf48 | 1.044513854 | 3.855927602 | 10.65808914 | 1.19E-21 | 1.79E-20 | 38.4253128 |
| NDRG2 | -1.029520491 | 6.299049774 | -10.65716075 | 1.20E-21 | 1.80E-20 | 38.41880123 |
| HSPB1 | 1.397485482 | 7.731357466 | 10.64894599 | 1.27E-21 | 1.90E-20 | 38.36119197 |
| MCM4 | 1.130488634 | 2.3939819 | 10.6306622 | 1.45E-21 | 2.14E-20 | 38.23301994 |
| PBLD | -1.313973785 | 5.116244344 | -10.60952873 | 1.68E-21 | 2.46E-20 | 38.08495815 |
| RECQL4 | 1.168410486 | 1.811312217 | 10.57319637 | 2.17E-21 | 3.15E-20 | 37.83063231 |
| GLS2 | -2.422474697 | 4.255520362 | -10.55610479 | 2.45E-21 | 3.54E-20 | 37.71108851 |
| TMEM64 | 1.043077816 | 2.453393665 | 10.54293035 | 2.69E-21 | 3.87E-20 | 37.61898501 |
| CNTFR | -1.10188817 | 1.201221719 | -10.53172769 | 2.91E-21 | 4.16E-20 | 37.54069571 |
| NAPSB | -1.248605442 | 2.097013575 | -10.50854455 | 3.43E-21 | 4.86E-20 | 37.37876745 |
| MCM2 | 1.166331508 | 1.86158371 | 10.50219847 | 3.58E-21 | 5.06E-20 | 37.33446202 |
| SLC22A1 | -2.858325037 | 5.892126697 | -10.49515337 | 3.77E-21 | 5.29E-20 | 37.28528665 |
| KRT7 | -2.249773519 | 2.807149321 | -10.49474551 | 3.78E-21 | 5.30E-20 | 37.28244008 |
| TUBA1B | 1.098198938 | 5.829276018 | 10.4941073 | 3.79E-21 | 5.32E-20 | 37.27798593 |
| IL33 | -1.26348515 | 2.639140271 | -10.47756701 | 4.26E-21 | 5.94E-20 | 37.1625797 |
| ATOX1 | 1.046718102 | 6.949909502 | 10.46855803 | 4.54E-21 | 6.31E-20 | 37.09974681 |
| PRKAB2 | 1.045224822 | 3.826425339 | 10.46132056 | 4.78E-21 | 6.61E-20 | 37.04928217 |
| ATOH8 | -1.073846856 | 1.343665158 | -10.45349363 | 5.05E-21 | 6.96E-20 | 36.99472046 |
| FTL | 1.157669653 | 12.52257919 | 10.45015349 | 5.17E-21 | 7.12E-20 | 36.97144037 |
| BRP44L | -1.060044798 | 6.291040724 | -10.44630174 | 5.31E-21 | 7.31E-20 | 36.94459755 |
| H2AFZ | 1.06257176 | 5.558733032 | 10.44370645 | 5.41E-21 | 7.44E-20 | 36.92651286 |
| LRRC20 | 1.011309939 | 3.14561086 | 10.43930629 | 5.58E-21 | 7.65E-20 | 36.89585467 |
| SERPINB9 | -1.253359051 | 2.723755656 | -10.43056067 | 5.94E-21 | 8.10E-20 | 36.83493235 |
| SPON2 | 1.251872407 | 5.231131222 | 10.42952093 | 5.98E-21 | 8.15E-20 | 36.82769061 |
| P4HA2 | 1.098206405 | 2.912443439 | 10.42241663 | 6.29E-21 | 8.54E-20 | 36.77821591 |
| CD34 | 1.369346275 | 2.612941176 | 10.42094886 | 6.35E-21 | 8.63E-20 | 36.76799571 |
| AKR7A3 | -1.960627178 | 5.393076923 | -10.41487193 | 6.63E-21 | 8.99E-20 | 36.72568661 |
| DUSP9 | 1.73410735 | 1.349728507 | 10.37103841 | 9.02E-21 | 1.20E-19 | 36.42075292 |
| ASPH | 1.347517836 | 4.220588235 | 10.36949592 | 9.12E-21 | 1.21E-19 | 36.41003031 |
| ZFP36 | -1.401789447 | 6.705022624 | -10.36018305 | 9.74E-21 | 1.29E-19 | 36.34530338 |
| KLKB1 | -1.240607267 | 5.588914027 | -10.3405314 | 1.12E-20 | 1.47E-19 | 36.20878402 |
| TMEM150B | 1.255905094 | 1.856334842 | 10.31581896 | 1.33E-20 | 1.74E-19 | 36.03723335 |
| TACC3 | 1.073904928 | 2.372081448 | 10.31107737 | 1.37E-20 | 1.79E-19 | 36.00433391 |
| CNDP1 | -1.773376473 | 1.82361991 | -10.29717392 | 1.52E-20 | 1.96E-19 | 35.90789517 |
| HAUS4 | 1.028422101 | 3.760542986 | 10.27339617 | 1.79E-20 | 2.30E-19 | 35.74306931 |
| CEMP1 | 1.08516675 | 3.032941176 | 10.26979387 | 1.84E-20 | 2.36E-19 | 35.71810997 |
| SULF2 | -1.392487971 | 3.442036199 | -10.26962186 | 1.84E-20 | 2.36E-19 | 35.71691822 |
| RUSC1-AS1 | 1.218334163 | 3.827873303 | 10.26601413 | 1.89E-20 | 2.41E-19 | 35.69192437 |
| SLC19A3 | -1.030886013 | 2.524208145 | -10.26482717 | 1.90E-20 | 2.43E-19 | 35.68370198 |
| AKR1C2 | 1.924000332 | 6.046742081 | 10.26072143 | 1.96E-20 | 2.50E-19 | 35.6552629 |
| TRIM15 | -1.381399535 | 2.990452489 | -10.19216801 | 3.16E-20 | 3.95E-19 | 35.18100572 |
| DUSP6 | -1.115169238 | 4.995972851 | -10.17616357 | 3.53E-20 | 4.41E-19 | 35.07044758 |
| MPPED1 | -1.337755932 | 2.14841629 | -10.15939477 | 3.97E-20 | 4.94E-19 | 34.95467556 |
| FOLH1B | -1.185340966 | 1.464162896 | -10.15828587 | 4.00E-20 | 4.97E-19 | 34.94702207 |
| CSRNP1 | -1.144016094 | 3.666244344 | -10.13469015 | 4.72E-20 | 5.80E-19 | 34.78423887 |
| ANXA10 | -1.594907085 | 3.504479638 | -10.13121455 | 4.84E-20 | 5.94E-19 | 34.76027271 |
| RDH16 | -2.053414634 | 6.427149321 | -10.12463172 | 5.06E-20 | 6.20E-19 | 34.71488865 |
| GLI4 | 1.148515016 | 2.684524887 | 10.11731709 | 5.33E-20 | 6.51E-19 | 34.66447175 |
| GPD1 | -1.551803551 | 4.31 | -10.08526265 | 6.66E-20 | 8.05E-19 | 34.44368846 |
| EGR1 | -1.936770367 | 5.012579186 | -10.07412721 | 7.20E-20 | 8.68E-19 | 34.36704972 |
| MT1H | -2.864514684 | 3.776334842 | -10.06920496 | 7.45E-20 | 8.96E-19 | 34.33318263 |
| CYP2C9 | -2.000550854 | 8.122081448 | -10.0533153 | 8.32E-20 | 9.94E-19 | 34.22389639 |
| PHYHIPL | 1.078502572 | 1.79361991 | 10.051933 | 8.40E-20 | 1.00E-18 | 34.21439215 |
| TREH | -1.260693546 | 1.648959276 | -10.04853267 | 8.60E-20 | 1.03E-18 | 34.1910147 |
| HMGB2 | 1.126768707 | 4.287013575 | 10.02187782 | 1.03E-19 | 1.22E-18 | 34.00786174 |
| HIST1H3H | 1.018039655 | 0.823846154 | 10.00882762 | 1.13E-19 | 1.34E-18 | 33.9182555 |
| CA2 | -1.316836735 | 4.813936652 | -10.00097895 | 1.20E-19 | 1.41E-18 | 33.86438527 |
| CYR61 | -1.771148167 | 4.836696833 | -9.992490999 | 1.27E-19 | 1.49E-18 | 33.80614482 |
| S100A10 | 1.166054422 | 6.00959276 | 9.979452099 | 1.39E-19 | 1.62E-18 | 33.7167137 |
| F3 | -1.118824457 | 1.473122172 | -9.945933275 | 1.75E-19 | 2.02E-18 | 33.48701519 |
| FAM99A | -2.031495769 | 2.832307692 | -9.938327937 | 1.85E-19 | 2.12E-18 | 33.4349375 |
| ETS2 | -1.056221171 | 5.827556561 | -9.937168506 | 1.86E-19 | 2.13E-18 | 33.42699959 |
| HSD17B2 | -1.544965157 | 5.516561086 | -9.909617889 | 2.25E-19 | 2.55E-18 | 33.23848032 |
| BGN | -1.895688568 | 5.619321267 | -9.89556876 | 2.48E-19 | 2.79E-18 | 33.1424233 |
| PRELP | -1.337338643 | 1.720995475 | -9.892715461 | 2.53E-19 | 2.84E-18 | 33.12292099 |
| TMEM45A | -1.637238261 | 3.62438914 | -9.866574074 | 3.03E-19 | 3.38E-18 | 32.94434419 |
| SRD5A2 | -1.614300647 | 2.809502262 | -9.848269418 | 3.44E-19 | 3.82E-18 | 32.81940916 |
| TMEM82 | -1.519158785 | 2.837556561 | -9.822784342 | 4.10E-19 | 4.51E-18 | 32.64561409 |
| C8A | -1.371626846 | 6.631809955 | -9.822032688 | 4.13E-19 | 4.53E-18 | 32.64049083 |
| QSOX1 | -1.389933632 | 3.130090498 | -9.814233454 | 4.35E-19 | 4.77E-18 | 32.58734038 |
| PLIN2 | -1.369070848 | 7.449457014 | -9.811727401 | 4.43E-19 | 4.84E-18 | 32.57026552 |
| CYP4A11 | -1.698330015 | 7.058959276 | -9.808877738 | 4.52E-19 | 4.93E-18 | 32.55085154 |
| CYP4A22 | -1.657093081 | 4.332624434 | -9.803016023 | 4.70E-19 | 5.12E-18 | 32.51092416 |
| AFM | -1.832224988 | 7.03760181 | -9.787435867 | 5.24E-19 | 5.67E-18 | 32.40484415 |
| ODZ1 | -1.101271777 | 1.374932127 | -9.740041672 | 7.26E-19 | 7.73E-18 | 32.08255842 |
| PODN | -1.382711133 | 1.877375566 | -9.733286137 | 7.60E-19 | 8.09E-18 | 32.03667004 |
| MIR650 | -2.107733532 | 2.269366516 | -9.732764024 | 7.63E-19 | 8.11E-18 | 32.033124 |
| ZG16 | -1.270642111 | 2.420769231 | -9.730911152 | 7.73E-19 | 8.19E-18 | 32.02054044 |
| STRA13 | 1.165732537 | 5.018597285 | 9.719032613 | 8.38E-19 | 8.85E-18 | 31.93989121 |
| C6orf97 | 1.035132736 | 0.988461538 | 9.704419072 | 9.27E-19 | 9.75E-18 | 31.84072616 |
| CXCR2P1 | -1.315888502 | 2.176199095 | -9.691631093 | 1.01E-18 | 1.06E-17 | 31.75399763 |
| COL15A1 | 1.232638958 | 1.401855204 | 9.687648331 | 1.04E-18 | 1.09E-17 | 31.72699568 |
| STEAP3 | -1.284928654 | 4.592307692 | -9.68450693 | 1.06E-18 | 1.11E-17 | 31.70570103 |
| THBS4 | 1.141383773 | 1.004841629 | 9.680357124 | 1.09E-18 | 1.14E-17 | 31.6775749 |
| THRSP | -2.614115646 | 4.112533937 | -9.672064638 | 1.16E-18 | 1.21E-17 | 31.62138536 |
| ALDH8A1 | -1.379801726 | 4.978280543 | -9.653769879 | 1.31E-18 | 1.35E-17 | 31.49748893 |
| MSH5-C6orf26 | 1.024382778 | 2.496968326 | 9.638974729 | 1.45E-18 | 1.49E-17 | 31.39736149 |
| PABPC1 | 1.035129418 | 7.199276018 | 9.624263575 | 1.61E-18 | 1.64E-17 | 31.29786378 |
| SP5 | 1.318350755 | 1.419457014 | 9.620579342 | 1.65E-18 | 1.68E-17 | 31.27295536 |
| EPHX2 | -1.03143106 | 5.672986425 | -9.609294864 | 1.78E-18 | 1.80E-17 | 31.19668708 |
| IGF2BP3 | 1.21705492 | 1.001855204 | 9.56650372 | 2.38E-18 | 2.38E-17 | 30.9078054 |
| ACSM3 | -1.104230131 | 4.321040724 | -9.561212217 | 2.47E-18 | 2.47E-17 | 30.87211915 |
| HRCT1 | 1.537519496 | 1.745927602 | 9.550906753 | 2.65E-18 | 2.64E-17 | 30.80264162 |
| RRS1 | 1.132464742 | 3.328959276 | 9.548056859 | 2.70E-18 | 2.68E-17 | 30.78343358 |
| RNF43 | 1.222668824 | 2.238959276 | 9.525341886 | 3.15E-18 | 3.11E-17 | 30.63042085 |
| CXCL2 | -2.004097395 | 5.257556561 | -9.516003075 | 3.36E-18 | 3.30E-17 | 30.56755626 |
| LAMA3 | 1.332923511 | 1.742669683 | 9.501723028 | 3.70E-18 | 3.62E-17 | 30.47147882 |
| C11orf96 | -1.705775676 | 3.651447964 | -9.500837236 | 3.73E-18 | 3.64E-17 | 30.4655211 |
| RAC3 | 1.151121619 | 2.884751131 | 9.496968546 | 3.83E-18 | 3.73E-17 | 30.4395035 |
| TBX15 | -1.528052928 | 2.465565611 | -9.488758574 | 4.05E-18 | 3.93E-17 | 30.38430459 |
| FBLN5 | -1.530770699 | 3.272624434 | -9.483191433 | 4.20E-18 | 4.07E-17 | 30.34688578 |
| MAGEA1 | 1.662747636 | 0.999502262 | 9.477887212 | 4.36E-18 | 4.21E-17 | 30.31124264 |
| SLC44A5 | 1.151127427 | 0.878325792 | 9.472521676 | 4.52E-18 | 4.35E-17 | 30.27519593 |
| PCOLCE2 | 1.42809109 | 2.429411765 | 9.432302935 | 5.94E-18 | 5.66E-17 | 30.00527049 |
| RDH5 | -1.165089597 | 4.178597285 | -9.429789306 | 6.04E-18 | 5.75E-17 | 29.98841643 |
| LPA | -1.147410818 | 3.029457014 | -9.420765851 | 6.42E-18 | 6.09E-17 | 29.9279291 |
| SLC22A10 | -1.704367015 | 3.705701357 | -9.402422852 | 7.27E-18 | 6.86E-17 | 29.80504497 |
| PROZ | -1.626606935 | 4.16 | -9.394457873 | 7.68E-18 | 7.22E-17 | 29.7517172 |
| XDH | -1.32595487 | 3.586923077 | -9.392555622 | 7.78E-18 | 7.30E-17 | 29.73898393 |
| THY1 | 1.49697196 | 2.848054299 | 9.389076025 | 7.96E-18 | 7.46E-17 | 29.71569505 |
| MS4A6A | -1.515794757 | 4.456877828 | -9.380820508 | 8.42E-18 | 7.87E-17 | 29.66045571 |
| HPS3 | -1.197726896 | 6.824208145 | -9.371933493 | 8.94E-18 | 8.33E-17 | 29.60101396 |
| VNN1 | -2.144438361 | 4.398325792 | -9.359447054 | 9.73E-18 | 9.02E-17 | 29.51753764 |
| GSTZ1 | -1.14630745 | 4.428642534 | -9.314843754 | 1.32E-17 | 1.20E-16 | 29.21973767 |
| KRTCAP3 | -1.107848017 | 2.204027149 | -9.309052404 | 1.37E-17 | 1.25E-16 | 29.18111578 |
| F9 | -1.853938941 | 6.167149321 | -9.290567713 | 1.55E-17 | 1.41E-16 | 29.05791276 |
| PZP | -1.334722084 | 1.308778281 | -9.289147775 | 1.56E-17 | 1.42E-16 | 29.04845306 |
| DUSP5 | -1.32964659 | 3.553031674 | -9.279663836 | 1.67E-17 | 1.51E-16 | 28.98528661 |
| FOS | -1.937935955 | 5.723665158 | -9.259282644 | 1.91E-17 | 1.72E-16 | 28.84963536 |
| UGT2B7 | -1.759129749 | 6.831855204 | -9.233999489 | 2.27E-17 | 2.02E-16 | 28.68153882 |
| AKR1D1 | -1.769562801 | 4.272579186 | -9.231188367 | 2.31E-17 | 2.06E-16 | 28.66286133 |
| SPSB2 | 1.061212875 | 2.332262443 | 9.222471764 | 2.45E-17 | 2.17E-16 | 28.60496282 |
| SHISA4 | 1.252538576 | 2.302624434 | 9.215849526 | 2.56E-17 | 2.27E-16 | 28.56099181 |
| RBP7 | 1.126470881 | 3.015113122 | 9.214663251 | 2.58E-17 | 2.28E-16 | 28.55311653 |
| G6PD | 1.254060893 | 2.7939819 | 9.184682732 | 3.16E-17 | 2.76E-16 | 28.35423417 |
| DBH | -1.09923262 | 2.874434389 | -9.169526729 | 3.50E-17 | 3.04E-16 | 28.2538025 |
| ACAA2 | -1.00080969 | 7.232171946 | -9.142336835 | 4.20E-17 | 3.62E-16 | 28.07381255 |
| GYS2 | -1.565081301 | 4.243936652 | -9.142039042 | 4.21E-17 | 3.63E-16 | 28.07184255 |
| APOA1 | -1.57838228 | 12.25357466 | -9.138396542 | 4.31E-17 | 3.71E-16 | 28.04774859 |
| GLYATL1 | -1.590049776 | 5.417285068 | -9.121565229 | 4.83E-17 | 4.13E-16 | 27.93647059 |
| HP | -1.877735192 | 12.06778281 | -9.082515524 | 6.27E-17 | 5.29E-16 | 27.6786532 |
| PYROXD2 | -1.301069355 | 2.343936652 | -9.07368067 | 6.65E-17 | 5.59E-16 | 27.62039207 |
| COX7B2 | 1.656174714 | 0.967782805 | 9.069609839 | 6.83E-17 | 5.74E-16 | 27.59355574 |
| HBB | -1.684829932 | 4.430452489 | -9.069371119 | 6.84E-17 | 5.75E-16 | 27.59198219 |
| GLUL | 1.844037664 | 7.598054299 | 9.060608937 | 7.25E-17 | 6.08E-16 | 27.53423802 |
| HIST1H2BK | 1.085439688 | 5.670950226 | 9.052741444 | 7.64E-17 | 6.37E-16 | 27.48241155 |
| HGFAC | -2.454324705 | 4.852895928 | -9.049790202 | 7.80E-17 | 6.48E-16 | 27.46297576 |
| EPS8L3 | 1.411445993 | 1.354841629 | 9.029653391 | 8.92E-17 | 7.38E-16 | 27.330439 |
| FGFR2 | -1.784275759 | 3.128778281 | -9.022100991 | 9.38E-17 | 7.75E-16 | 27.28076519 |
| MAP2 | 1.206901444 | 2.058959276 | 9.003732632 | 1.06E-16 | 8.72E-16 | 27.1600317 |
| CP | -1.281823461 | 8.145158371 | -8.983480998 | 1.21E-16 | 9.89E-16 | 27.02705033 |
| ZNF692 | 1.045598971 | 3.83959276 | 8.971754168 | 1.31E-16 | 1.07E-15 | 26.95010962 |
| WDR72 | -1.101298324 | 2.141447964 | -8.950549271 | 1.51E-16 | 1.22E-15 | 26.81110027 |
| SPP2 | -2.13510287 | 6.04199095 | -8.950404246 | 1.51E-16 | 1.22E-15 | 26.81015008 |
| CYP26A1 | -1.41574664 | 1.520723982 | -8.928777128 | 1.74E-16 | 1.39E-15 | 26.66853082 |
| SAA4 | -1.961854986 | 7.704434389 | -8.927687865 | 1.76E-16 | 1.40E-15 | 26.66140228 |
| IFITM1 | -1.541872407 | 5.797466063 | -8.898288421 | 2.13E-16 | 1.69E-15 | 26.46915465 |
| EPO | -1.271083458 | 1.309095023 | -8.889605008 | 2.26E-16 | 1.78E-15 | 26.41242908 |
| MT1L | -1.664436702 | 3.295067873 | -8.879674058 | 2.41E-16 | 1.89E-15 | 26.3475856 |
| MIR1287 | -1.091606935 | 1.512760181 | -8.880466347 | 2.40E-16 | 1.89E-15 | 26.35275755 |
| NNMT | -2.206092583 | 6.750950226 | -8.878292842 | 2.44E-16 | 1.91E-15 | 26.33856973 |
| ARMCX3 | -1.07879625 | 2.648461538 | -8.820979442 | 3.56E-16 | 2.74E-15 | 25.96504018 |
| LTBP4 | -1.138581384 | 2.797330317 | -8.796330163 | 4.18E-16 | 3.19E-15 | 25.80474545 |
| AKR1B15 | 1.439931973 | 1.268144796 | 8.744386452 | 5.89E-16 | 4.43E-15 | 25.46765616 |
| PTGDS | -2.239580222 | 3.870271493 | -8.732595462 | 6.36E-16 | 4.76E-15 | 25.39127189 |
| C5orf45 | 1.147934296 | 5.187058824 | 8.713437078 | 7.22E-16 | 5.38E-15 | 25.26726628 |
| ENG | -1.012631492 | 4.393755656 | -8.70734434 | 7.51E-16 | 5.58E-15 | 25.22785768 |
| PANX2 | 1.061684918 | 2.081402715 | 8.706480168 | 7.55E-16 | 5.61E-15 | 25.22226919 |
| HIST2H2BE | 1.060914219 | 2.655565611 | 8.6944853 | 8.17E-16 | 6.04E-15 | 25.14472763 |
| TDGF1 | 1.171122449 | 1.130678733 | 8.677526303 | 9.13E-16 | 6.72E-15 | 25.03518377 |
| WNT11 | -1.164034345 | 1.54959276 | -8.672448712 | 9.44E-16 | 6.94E-15 | 25.00240614 |
| ACSL1 | -1.049221005 | 7.329411765 | -8.654492722 | 1.06E-15 | 7.76E-15 | 24.88656903 |
| SLC30A10 | 1.105886013 | 4.312533937 | 8.650909924 | 1.09E-15 | 7.93E-15 | 24.86346983 |
| IDO2 | -1.372624025 | 1.766561086 | -8.64007327 | 1.17E-15 | 8.48E-15 | 24.79363172 |
| CXCL6 | -1.537118799 | 1.610723982 | -8.638351774 | 1.18E-15 | 8.57E-15 | 24.78254128 |
| LAPTM4B | 1.284138875 | 4.111538462 | 8.635507559 | 1.20E-15 | 8.72E-15 | 24.76422029 |
| IGLL5 | -2.8033408 | 5.286606335 | -8.620620552 | 1.32E-15 | 9.56E-15 | 24.66837408 |
| SSX1 | 1.847708644 | 1.082036199 | 8.616795038 | 1.36E-15 | 9.79E-15 | 24.64375762 |
| C6 | -1.582852995 | 6.305882353 | -8.601797844 | 1.50E-15 | 1.07E-14 | 24.5473054 |
| GZMK | -1.31319562 | 2.505248869 | -8.59853371 | 1.53E-15 | 1.10E-14 | 24.52632359 |
| SCARA3 | 1.023356562 | 1.613846154 | 8.59636782 | 1.55E-15 | 1.11E-14 | 24.51240346 |
| FKBP1A-SDCBP2 | -1.041587855 | 3.020904977 | -8.590602854 | 1.61E-15 | 1.15E-14 | 24.47536056 |
| VSIG4 | -1.394847354 | 2.951131222 | -8.587355384 | 1.65E-15 | 1.18E-14 | 24.45449929 |
| LUM | -2.256733035 | 3.850316742 | -8.558468477 | 1.99E-15 | 1.41E-14 | 24.26910617 |
| SLC7A2 | -1.177151153 | 4.554027149 | -8.55069065 | 2.09E-15 | 1.48E-14 | 24.21924194 |
| FAM110C | -1.063420441 | 2.474570136 | -8.528374245 | 2.41E-15 | 1.69E-14 | 24.07629533 |
| CCL2 | -1.815920856 | 4.730859729 | -8.528757187 | 2.41E-15 | 1.69E-14 | 24.07874667 |
| F11 | -1.055034014 | 5.311176471 | -8.52913858 | 2.40E-15 | 1.69E-14 | 24.08118815 |
| HMGA1 | 1.037712792 | 3.96520362 | 8.525740025 | 2.46E-15 | 1.72E-14 | 24.05943428 |
| BMP4 | 1.26964742 | 1.998371041 | 8.509153761 | 2.74E-15 | 1.90E-14 | 23.95332925 |
| GAS5 | 1.043865937 | 6.559954751 | 8.507849289 | 2.76E-15 | 1.92E-14 | 23.94498873 |
| RBP1 | -1.818450307 | 4.636696833 | -8.504457919 | 2.82E-15 | 1.96E-14 | 23.923308 |
| GCGR | -2.165900946 | 4.011176471 | -8.49855225 | 2.93E-15 | 2.03E-14 | 23.88556396 |
| BASP1 | -1.088587191 | 2.36158371 | -8.492072058 | 3.06E-15 | 2.11E-14 | 23.84416323 |
| CELF6 | 1.045326862 | 1.99638009 | 8.485402476 | 3.19E-15 | 2.20E-14 | 23.80156912 |
| FGB | -1.195237266 | 10.6381448 | -8.481888624 | 3.26E-15 | 2.24E-14 | 23.7791353 |
| ALDH3A1 | 2.353232122 | 1.824615385 | 8.479858115 | 3.31E-15 | 2.27E-14 | 23.76617386 |
| FOSB | -1.760019081 | 2.800135747 | -8.469123843 | 3.55E-15 | 2.43E-14 | 23.69767934 |
| PAFAH1B3 | 1.007464742 | 2.44438914 | 8.463364404 | 3.68E-15 | 2.52E-14 | 23.6609469 |
| ELOVL2 | 1.154452464 | 3.892217195 | 8.447128461 | 4.09E-15 | 2.78E-14 | 23.5574657 |
| CYP2A6 | -2.717766716 | 6.27800905 | -8.433292487 | 4.47E-15 | 3.03E-14 | 23.46936047 |
| ASPSCR1 | 1.068646922 | 4.312624434 | 8.43113833 | 4.53E-15 | 3.07E-14 | 23.45564975 |
| C10orf116 | -1.491234445 | 3.933710407 | -8.383925698 | 6.14E-15 | 4.11E-14 | 23.15560106 |
| EPCAM | -2.042086444 | 2.251719457 | -8.382232441 | 6.21E-15 | 4.15E-14 | 23.14485598 |
| SAA2-SAA4 | -1.847079808 | 7.43361991 | -8.379489415 | 6.32E-15 | 4.22E-14 | 23.12745164 |
| CYP2B7P1 | -2.325354239 | 4.532941176 | -8.360182017 | 7.16E-15 | 4.76E-14 | 23.00503009 |
| FABP1 | -1.710528455 | 10.45298643 | -8.353837774 | 7.46E-15 | 4.94E-14 | 22.96483516 |
| SULT1C2 | 1.533348266 | 1.689728507 | 8.347185154 | 7.78E-15 | 5.15E-14 | 22.92270335 |
| GBP1 | -1.077621536 | 4.930135747 | -8.344370817 | 7.93E-15 | 5.24E-14 | 22.90488505 |
| SLCO1B3 | -2.116277584 | 3.871447964 | -8.336611064 | 8.33E-15 | 5.49E-14 | 22.85577209 |
| ALPL | -1.209105691 | 3.407058824 | -8.33519518 | 8.41E-15 | 5.54E-14 | 22.84681323 |
| INS-IGF2 | -3.00902439 | 5.545294118 | -8.334385419 | 8.45E-15 | 5.57E-14 | 22.84168991 |
| C1QTNF3 | 1.126095902 | 1.987149321 | 8.319739812 | 9.28E-15 | 6.09E-14 | 22.74907229 |
| IGF2 | -3.001498258 | 5.568054299 | -8.313954854 | 9.63E-15 | 6.30E-14 | 22.71251195 |
| HPX | -1.248793761 | 9.848235294 | -8.284693099 | 1.16E-14 | 7.54E-14 | 22.52778325 |
| NRAP | -1.212960843 | 4.098280543 | -8.273161861 | 1.25E-14 | 8.07E-14 | 22.4550801 |
| CPLX2 | 1.813476854 | 1.039004525 | 8.269971867 | 1.28E-14 | 8.22E-14 | 22.43497689 |
| IGF2BP1 | 1.081827609 | 0.647511312 | 8.249833988 | 1.45E-14 | 9.29E-14 | 22.3081625 |
| CDA | -1.43400448 | 3.55199095 | -8.241304471 | 1.53E-14 | 9.79E-14 | 22.2544984 |
| SERPINA4 | -1.057596648 | 6.765158371 | -8.20750633 | 1.90E-14 | 1.20E-13 | 22.04214219 |
| COL7A1 | 1.231103368 | 1.413755656 | 8.199724298 | 2.00E-14 | 1.26E-13 | 21.99331234 |
| ALDOB | -1.772616559 | 10.13710407 | -8.195568205 | 2.05E-14 | 1.29E-13 | 21.96724418 |
| ADH4 | -2.247410818 | 8.227239819 | -8.195304378 | 2.06E-14 | 1.30E-13 | 21.96558962 |
| NUPR1 | 1.119756098 | 7.05321267 | 8.189807209 | 2.13E-14 | 1.34E-13 | 21.9311212 |
| HSPB6 | -1.337555168 | 2.859140271 | -8.16144745 | 2.55E-14 | 1.59E-13 | 21.75349451 |
| MS4A1 | -1.051739671 | 1.17760181 | -8.14808979 | 2.78E-14 | 1.72E-13 | 21.66994461 |
| ISX | 1.060798905 | 0.658868778 | 8.108240157 | 3.57E-14 | 2.19E-13 | 21.42112676 |
| PRNP | -1.112507052 | 3.997556561 | -8.101483384 | 3.73E-14 | 2.29E-13 | 21.37900291 |
| CYP3A4 | -3.074038493 | 7.442579186 | -8.084631298 | 4.15E-14 | 2.53E-13 | 21.27402401 |
| CLGN | 1.145107848 | 1.125791855 | 8.056929199 | 4.94E-14 | 2.99E-13 | 21.10171215 |
| FPR1 | -1.133953045 | 1.846334842 | -8.042121875 | 5.43E-14 | 3.26E-13 | 21.00973937 |
| SFN | 1.570177534 | 1.420226244 | 8.041234704 | 5.46E-14 | 3.28E-13 | 21.0042318 |
| PHGDH | -1.334391903 | 4.577737557 | -8.028491578 | 5.92E-14 | 3.54E-13 | 20.92515868 |
| PCK1 | -2.059610088 | 7.687782805 | -8.019733045 | 6.25E-14 | 3.74E-13 | 20.87085016 |
| FAM134B | -1.169059233 | 2.167918552 | -8.008042112 | 6.73E-14 | 4.01E-13 | 20.79840922 |
| GALK1 | 1.008964659 | 4.906244344 | 8.006490043 | 6.79E-14 | 4.04E-13 | 20.78879642 |
| CD163 | -1.318406338 | 3.72918552 | -7.981125039 | 7.97E-14 | 4.71E-13 | 20.63184166 |
| C8B | -1.179651568 | 6.847737557 | -7.980508865 | 8.00E-14 | 4.73E-13 | 20.62803227 |
| TRIM22 | -1.142475527 | 3.993122172 | -7.955102469 | 9.38E-14 | 5.52E-13 | 20.47110196 |
| MS4A7 | -1.053934793 | 3.42638009 | -7.930697901 | 1.09E-13 | 6.39E-13 | 20.32061931 |
| COL5A3 | 1.258047951 | 2.325384615 | 7.92803809 | 1.11E-13 | 6.49E-13 | 20.3042339 |
| CH25H | -1.095692716 | 1.440588235 | -7.896722071 | 1.35E-13 | 7.81E-13 | 20.11154476 |
| APOA5 | -1.67560146 | 5.915384615 | -7.887876842 | 1.43E-13 | 8.24E-13 | 20.05719629 |
| CRISPLD2 | -1.206007964 | 2.116742081 | -7.861034547 | 1.69E-13 | 9.65E-13 | 19.89247476 |
| GADD45B | -1.250355898 | 6.106651584 | -7.841758288 | 1.90E-13 | 1.08E-12 | 19.77437689 |
| GDA | -1.198989547 | 3.624117647 | -7.825832229 | 2.10E-13 | 1.19E-12 | 19.67692697 |
| LIPG | -1.015438858 | 3.52280543 | -7.812433355 | 2.29E-13 | 1.29E-12 | 19.59502689 |
| S100A8 | -1.230896798 | 2.600542986 | -7.807370799 | 2.36E-13 | 1.33E-12 | 19.56410278 |
| IFI16 | -1.016568774 | 3.790135747 | -7.779241713 | 2.81E-13 | 1.57E-12 | 19.39248524 |
| ANXA2 | 1.074756927 | 6.247963801 | 7.764671989 | 3.07E-13 | 1.71E-12 | 19.30373211 |
| RGS5 | 1.200937448 | 3.267782805 | 7.757626878 | 3.21E-13 | 1.78E-12 | 19.26084987 |
| HAL | -1.822888668 | 4.506063348 | -7.730518714 | 3.80E-13 | 2.09E-12 | 19.09605415 |
| EMILIN1 | -1.098682595 | 2.324434389 | -7.726301041 | 3.90E-13 | 2.14E-12 | 19.07044365 |
| FXYD1 | -1.6872001 | 6.18719457 | -7.721715255 | 4.01E-13 | 2.20E-12 | 19.04260696 |
| SLC22A11 | 1.523062054 | 1.259411765 | 7.713243977 | 4.22E-13 | 2.31E-12 | 18.99120933 |
| CD69 | -1.098419612 | 2.064886878 | -7.697226681 | 4.66E-13 | 2.54E-12 | 18.89411611 |
| NCRNA00221 | 1.046898125 | 0.610723982 | 7.686949029 | 4.97E-13 | 2.70E-12 | 18.83187623 |
| TTR | -1.259082462 | 10.78352941 | -7.662048931 | 5.79E-13 | 3.12E-12 | 18.68128328 |
| ACSL4 | 1.849589348 | 3.675429864 | 7.630220345 | 7.04E-13 | 3.75E-12 | 18.48919805 |
| ITIH4 | -1.071109175 | 9.091719457 | -7.628276037 | 7.12E-13 | 3.80E-12 | 18.47747915 |
| SQLE | 1.38282064 | 3.935746606 | 7.616301386 | 7.67E-13 | 4.07E-12 | 18.40534271 |
| PALM3 | -1.22421188 | 3.309773756 | -7.610854916 | 7.93E-13 | 4.21E-12 | 18.3725544 |
| MMP11 | 1.284728721 | 1.631764706 | 7.608334519 | 8.05E-13 | 4.27E-12 | 18.35738596 |
| C1orf162 | -1.029619214 | 3.898280543 | -7.603416139 | 8.29E-13 | 4.40E-12 | 18.32779421 |
| ASPN | -1.206688236 | 2.923710407 | -7.601877256 | 8.37E-13 | 4.43E-12 | 18.3185377 |
| MZB1 | -1.553247055 | 2.344298643 | -7.589290841 | 9.04E-13 | 4.77E-12 | 18.24287035 |
| SNORD47 | 1.078645263 | 4.528597285 | 7.5859331 | 9.23E-13 | 4.86E-12 | 18.22269653 |
| WFDC2 | -1.242668824 | 1.459909502 | -7.563428374 | 1.06E-12 | 5.55E-12 | 18.08761919 |
| PPP1R3B | -1.119993363 | 4.435022624 | -7.555395843 | 1.11E-12 | 5.81E-12 | 18.03946334 |
| TREM2 | 1.155026547 | 1.651312217 | 7.553336788 | 1.13E-12 | 5.88E-12 | 18.02712392 |
| SLC10A1 | -1.804041812 | 5.950226244 | -7.496794833 | 1.59E-12 | 8.19E-12 | 17.68905419 |
| CYP4F2 | -1.171260163 | 4.883122172 | -7.494821177 | 1.61E-12 | 8.28E-12 | 17.67728055 |
| CYP2C19 | -1.009059233 | 2.886968326 | -7.463952167 | 1.94E-12 | 9.90E-12 | 17.49337357 |
| ID4 | -1.010897627 | 1.608190045 | -7.440440917 | 2.24E-12 | 1.13E-11 | 17.35360393 |
| TMEM98 | 1.118541563 | 3.571312217 | 7.411557652 | 2.66E-12 | 1.34E-11 | 17.18225857 |
| HIST1H2AE | 1.016108346 | 1.587873303 | 7.397697194 | 2.89E-12 | 1.45E-11 | 17.10017511 |
| HIST1H1C | 1.217459764 | 5.822488688 | 7.381957841 | 3.18E-12 | 1.59E-11 | 17.00707628 |
| FGG | -1.051927991 | 11.39882353 | -7.364913207 | 3.52E-12 | 1.75E-11 | 16.90639107 |
| MIR4751 | -1.635774847 | 6.02520362 | -7.361029015 | 3.61E-12 | 1.79E-11 | 16.88346616 |
| PEG10 | 1.957798241 | 1.971266968 | 7.346481566 | 3.94E-12 | 1.95E-11 | 16.79767039 |
| CYP17A1 | 1.50620375 | 1.732171946 | 7.32447191 | 4.49E-12 | 2.21E-11 | 16.66806009 |
| ATF5 | -1.575690227 | 6.447828054 | -7.32271646 | 4.54E-12 | 2.23E-11 | 16.65773273 |
| HSD17B14 | 1.246952879 | 2.808597285 | 7.31840833 | 4.66E-12 | 2.29E-11 | 16.63239428 |
| DEFB1 | -2.237414136 | 5.69361991 | -7.306340895 | 5.01E-12 | 2.45E-11 | 16.56146727 |
| MAGEC2 | 1.300699353 | 0.776877828 | 7.297242698 | 5.29E-12 | 2.58E-11 | 16.50803912 |
| HABP2 | -1.38294591 | 6.1 | -7.271966752 | 6.15E-12 | 2.98E-11 | 16.35982161 |
| FASN | 1.058957193 | 5.240904977 | 7.268812349 | 6.27E-12 | 3.03E-11 | 16.34134628 |
| MPZ | 1.167059897 | 1.63280543 | 7.268548345 | 6.28E-12 | 3.04E-11 | 16.33980024 |
| ENO3 | -1.249744483 | 4.20158371 | -7.229535921 | 7.92E-12 | 3.79E-11 | 16.11171511 |
| MBL2 | -1.21632736 | 4.764570136 | -7.226729926 | 8.05E-12 | 3.85E-11 | 16.09533893 |
| TNFRSF11B | -1.03224075 | 2.101719457 | -7.219511962 | 8.41E-12 | 4.01E-11 | 16.05323186 |
| S100P | 2.149556994 | 2.025339367 | 7.217119852 | 8.53E-12 | 4.07E-11 | 16.03928282 |
| BBOX1 | -1.284292351 | 2.521131222 | -7.181765262 | 1.05E-11 | 4.98E-11 | 15.83345302 |
| FHL2 | -1.039516343 | 2.527466063 | -7.177042975 | 1.08E-11 | 5.11E-11 | 15.80600772 |
| MMP7 | -1.556960345 | 2.075701357 | -7.162826273 | 1.18E-11 | 5.53E-11 | 15.72344959 |
| ANG | -1.008126763 | 8.668099548 | -7.140898077 | 1.34E-11 | 6.27E-11 | 15.59630891 |
| LGR5 | 1.145468724 | 1.092217195 | 7.140028064 | 1.35E-11 | 6.30E-11 | 15.59126953 |
| CD79A | -1.077141198 | 1.557239819 | -7.127838 | 1.45E-11 | 6.75E-11 | 15.52070111 |
| PHYHD1 | -1.046756263 | 3.689457014 | -7.112431364 | 1.58E-11 | 7.36E-11 | 15.43161937 |
| ADH1A | -1.46740584 | 8.120135747 | -7.022198485 | 2.69E-11 | 1.22E-10 | 14.9123169 |
| GLYAT | -1.58724075 | 5.527828054 | -7.018373824 | 2.75E-11 | 1.25E-10 | 14.8903977 |
| CFHR3 | -1.664203584 | 5.562850679 | -7.008735089 | 2.91E-11 | 1.32E-10 | 14.83519143 |
| FCGR3A | -1.111776174 | 4.453167421 | -6.997372209 | 3.11E-11 | 1.40E-10 | 14.77017172 |
| ALPK2 | 1.035467894 | 1.516199095 | 6.961767806 | 3.83E-11 | 1.71E-10 | 14.56687305 |
| ASPDH | -1.184514684 | 4.731357466 | -6.927671109 | 4.66E-11 | 2.07E-10 | 14.37280291 |
| CYP8B1 | -1.762769205 | 5.220135747 | -6.901826345 | 5.42E-11 | 2.38E-10 | 14.22610707 |
| DCAF4L2 | 1.241890659 | 0.741085973 | 6.833615242 | 8.03E-11 | 3.47E-10 | 13.84063434 |
| OGDHL | -1.150402356 | 4.493710407 | -6.810158446 | 9.19E-11 | 3.95E-10 | 13.70864847 |
| AZGP1 | -1.171196283 | 9.012443439 | -6.80061986 | 9.71E-11 | 4.16E-10 | 13.65506141 |
| UGT2B10 | -1.106059399 | 5.755022624 | -6.792560077 | 1.02E-10 | 4.35E-10 | 13.60982025 |
| CSTA | 1.103685084 | 3.227737557 | 6.775759442 | 1.12E-10 | 4.77E-10 | 13.51562737 |
| MAGEA3 | 1.265418948 | 0.781221719 | 6.747058606 | 1.32E-10 | 5.57E-10 | 13.35506855 |
| SRGN | -1.10211548 | 5.067013575 | -6.720842838 | 1.53E-10 | 6.42E-10 | 13.20880217 |
| DSCR8 | 1.060777335 | 0.644162896 | 6.709375695 | 1.63E-10 | 6.84E-10 | 13.14494086 |
| COCH | 1.028674299 | 0.958642534 | 6.691158446 | 1.81E-10 | 7.56E-10 | 13.04363549 |
| HBA2 | -1.058982081 | 3.02520362 | -6.682925984 | 1.90E-10 | 7.90E-10 | 12.99791472 |
| SNORD104 | 1.020502738 | 4.474298643 | 6.673972161 | 2.00E-10 | 8.30E-10 | 12.94822994 |
| HPGD | -1.571946242 | 3.773891403 | -6.668835516 | 2.06E-10 | 8.52E-10 | 12.91974657 |
| GBP7 | -1.057249876 | 4.030045249 | -6.663826633 | 2.12E-10 | 8.76E-10 | 12.89198565 |
| AEBP1 | -1.325801394 | 3.394253394 | -6.604216872 | 2.96E-10 | 1.21E-09 | 12.56267199 |
| BHMT | -1.353141696 | 7.144751131 | -6.596100343 | 3.10E-10 | 1.26E-09 | 12.51798477 |
| THBS1 | -1.330794757 | 4.194841629 | -6.581308339 | 3.37E-10 | 1.36E-09 | 12.43663881 |
| CYP2E1 | -2.291019579 | 9.570723982 | -6.573251187 | 3.53E-10 | 1.42E-09 | 12.39238136 |
| CYP3A7 | -1.920922515 | 5.637828054 | -6.533307151 | 4.41E-10 | 1.76E-09 | 12.17350858 |
| FXYD2 | -1.759167911 | 2.961221719 | -6.518800439 | 4.78E-10 | 1.90E-09 | 12.09424146 |
| OSGIN1 | 1.095837896 | 5.748778281 | 6.507104477 | 5.11E-10 | 2.03E-09 | 12.03041929 |
| PAGE4 | 1.864196947 | 1.340090498 | 6.498463922 | 5.36E-10 | 2.12E-09 | 11.98331949 |
| GEM | -1.196359715 | 2.638642534 | -6.443344194 | 7.28E-10 | 2.84E-09 | 11.68385981 |
| AKR7L | -1.018138377 | 3.545384615 | -6.44136927 | 7.36E-10 | 2.87E-09 | 11.67316242 |
| RASD1 | -1.223618716 | 3.81918552 | -6.42493728 | 8.06E-10 | 3.13E-09 | 11.58424344 |
| ISLR | -1.042657209 | 1.688371041 | -6.42411561 | 8.10E-10 | 3.15E-09 | 11.57980117 |
| RAMP1 | 1.26723992 | 4.349276018 | 6.413999944 | 8.56E-10 | 3.32E-09 | 11.52514376 |
| NPC1L1 | -1.134870582 | 2.301538462 | -6.410252234 | 8.74E-10 | 3.39E-09 | 11.5049089 |
| NKD1 | 1.092700348 | 0.998868778 | 6.394164914 | 9.56E-10 | 3.69E-09 | 11.4181412 |
| VSIG2 | -1.026962004 | 1.931085973 | -6.385982998 | 1.00E-09 | 3.85E-09 | 11.37406893 |
| UBD | 1.575113655 | 7.108687783 | 6.381242607 | 1.03E-09 | 3.95E-09 | 11.3485523 |
| NPW | -1.205298656 | 2.717647059 | -6.378262822 | 1.04E-09 | 4.01E-09 | 11.33251932 |
| FABP5 | 1.092251535 | 3.764027149 | 6.360662679 | 1.15E-09 | 4.39E-09 | 11.23792524 |
| S100A14 | -1.272259831 | 2.927828054 | -6.338854128 | 1.30E-09 | 4.93E-09 | 11.12096204 |
| MAGEA6 | 1.168706653 | 0.72361991 | 6.317720395 | 1.45E-09 | 5.50E-09 | 11.00788237 |
| STEAP1 | 1.03416957 | 2.839864253 | 6.274642083 | 1.84E-09 | 6.89E-09 | 10.77819411 |
| PLG | -1.026948731 | 8.197828054 | -6.251887517 | 2.08E-09 | 7.75E-09 | 10.65731016 |
| NQO1 | 1.774091588 | 2.300859729 | 6.247852811 | 2.13E-09 | 7.91E-09 | 10.63590765 |
| CYP3A43 | -1.032156131 | 2.840950226 | -6.207040376 | 2.66E-09 | 9.77E-09 | 10.41995636 |
| SOCS3 | -1.256436038 | 3.7439819 | -6.202038983 | 2.73E-09 | 1.00E-08 | 10.39356054 |
| LCN2 | 1.899825784 | 3.710226244 | 6.131269631 | 4.00E-09 | 1.44E-08 | 10.0216655 |
| SLC22A12 | 1.110793927 | 0.757918552 | 6.124762838 | 4.14E-09 | 1.49E-08 | 9.987623205 |
| MEP1A | 1.094639954 | 0.74280543 | 6.084409376 | 5.14E-09 | 1.83E-08 | 9.777073444 |
| ADH1C | -1.607188485 | 8.018868778 | -6.066341754 | 5.66E-09 | 2.01E-08 | 9.683123473 |
| ACSM5 | -1.006272607 | 4.69800905 | -6.045316939 | 6.33E-09 | 2.24E-08 | 9.57404688 |
| CCL5 | -1.056012112 | 3.876244344 | -6.023851416 | 7.09E-09 | 2.50E-08 | 9.462962805 |
| TPM2 | 1.019857309 | 4.432714932 | 5.999720782 | 8.06E-09 | 2.83E-08 | 9.338424433 |
| SLC28A1 | -1.067194292 | 3.015837104 | -5.98535208 | 8.69E-09 | 3.04E-08 | 9.264437769 |
| MIR675 | -2.235660362 | 6.893167421 | -5.966685714 | 9.59E-09 | 3.34E-08 | 9.168512202 |
| APOC3 | -1.134483159 | 12.12144796 | -5.947788701 | 1.06E-08 | 3.68E-08 | 9.071621157 |
| H19 | -2.202559316 | 7.548733032 | -5.9407501 | 1.10E-08 | 3.81E-08 | 9.035588658 |
| SPARCL1 | 1.243561473 | 4.060995475 | 5.919587431 | 1.23E-08 | 4.23E-08 | 8.927436953 |
| FETUB | -1.133923179 | 5.319004525 | -5.883270372 | 1.49E-08 | 5.07E-08 | 8.742490599 |
| HULC | 1.135631326 | 7.616742081 | 5.816077359 | 2.11E-08 | 7.07E-08 | 8.402492857 |
| VNN3 | -1.112969968 | 3.361176471 | -5.807750546 | 2.20E-08 | 7.36E-08 | 8.360557837 |
| SPP1 | 2.105092086 | 5.148959276 | 5.731225547 | 3.26E-08 | 1.07E-07 | 7.977235828 |
| C1QC | -1.057101377 | 6.058597285 | -5.7169376 | 3.51E-08 | 1.15E-07 | 7.906081477 |
| C1QB | -1.070379957 | 6.637737557 | -5.689336912 | 4.04E-08 | 1.32E-07 | 7.769001807 |
| GTSF1 | 1.149092417 | 1.22280543 | 5.685474853 | 4.12E-08 | 1.34E-07 | 7.749860012 |
| ADH1B | -1.356222001 | 8.945791855 | -5.660633437 | 4.68E-08 | 1.51E-07 | 7.626967663 |
| REG3A | 1.979611747 | 1.357285068 | 5.657356539 | 4.76E-08 | 1.54E-07 | 7.610786488 |
| DHRS2 | 1.528346607 | 2.922352941 | 5.650343023 | 4.93E-08 | 1.59E-07 | 7.57617752 |
| PRG4 | -1.078283557 | 5.052624434 | -5.599725355 | 6.37E-08 | 2.03E-07 | 7.327349885 |
| HRG | -1.332035839 | 9.529457014 | -5.550338021 | 8.17E-08 | 2.57E-07 | 7.086188824 |
| SLC39A5 | -1.057867928 | 5.096742081 | -5.515480329 | 9.73E-08 | 3.03E-07 | 6.91694557 |
| AGXT | -1.097245728 | 8.971764706 | -5.488440983 | 1.11E-07 | 3.45E-07 | 6.786217607 |
| C3P1 | -1.198270284 | 5.282579186 | -5.469143056 | 1.23E-07 | 3.77E-07 | 6.693215125 |
| LGALS3BP | -1.015593994 | 6.345882353 | -5.464941635 | 1.25E-07 | 3.85E-07 | 6.673000184 |
| PPP1R1A | -1.174067529 | 3.952171946 | -5.454803526 | 1.32E-07 | 4.04E-07 | 6.624269758 |
| SLC27A5 | -1.040340136 | 6.594660633 | -5.414521127 | 1.61E-07 | 4.89E-07 | 6.431327016 |
| SERPINE1 | -1.121299983 | 4.990723982 | -5.398192163 | 1.74E-07 | 5.27E-07 | 6.353426258 |
| SLC22A7 | -1.212052431 | 5.950316742 | -5.373479918 | 1.96E-07 | 5.92E-07 | 6.235873794 |
| MUC13 | 1.184893811 | 1.920588235 | 5.31937342 | 2.56E-07 | 7.62E-07 | 5.97994397 |
| SLC13A3 | 1.053337481 | 2.210226244 | 5.297809365 | 2.84E-07 | 8.42E-07 | 5.878499776 |
| LECT2 | -1.142269786 | 5.932624434 | -5.293195311 | 2.91E-07 | 8.60E-07 | 5.856835125 |
| SULT2A1 | -1.055241414 | 7.862081448 | -5.244640463 | 3.67E-07 | 1.07E-06 | 5.629738989 |
| FXYD6-FXYD2 | -1.052682097 | 2.738914027 | -5.210598594 | 4.33E-07 | 1.26E-06 | 5.471491017 |
| GNMT | -1.232872905 | 5.435565611 | -5.183084915 | 4.94E-07 | 1.43E-06 | 5.344177095 |
| OSTalpha | -1.140615563 | 4.966606335 | -5.131668121 | 6.31E-07 | 1.80E-06 | 5.107670211 |
| SDS | -1.500997179 | 5.799909502 | -5.109726254 | 7.00E-07 | 1.99E-06 | 5.007305398 |
| G0S2 | -1.041204579 | 6.156832579 | -5.082062637 | 7.97E-07 | 2.26E-06 | 4.881251097 |
| HPD | -1.461626016 | 8.656515837 | -5.062881778 | 8.73E-07 | 2.46E-06 | 4.794166935 |
| RHBG | 1.531559648 | 2.908733032 | 4.888742648 | 1.96E-06 | 5.31E-06 | 4.015539955 |
| GPX2 | 1.148332504 | 6.579321267 | 4.809092491 | 2.81E-06 | 7.50E-06 | 3.666689408 |
| SERPINA7 | -1.038255351 | 6.457963801 | -4.693052949 | 4.73E-06 | 1.23E-05 | 3.166781222 |
| CHI3L1 | -1.519705492 | 6.486742081 | -4.667131076 | 5.31E-06 | 1.37E-05 | 3.056468665 |
| CYP7A1 | 1.243925668 | 3.586606335 | 4.622228381 | 6.47E-06 | 1.66E-05 | 2.866567241 |
| PLA2G2A | -1.744521321 | 5.311312217 | -4.474561428 | 1.23E-05 | 3.05E-05 | 2.252754001 |
| TIMP1 | -1.027245728 | 7.070723982 | -4.43828518 | 1.43E-05 | 3.53E-05 | 2.104496504 |
| AFP | 1.399488137 | 2.669819005 | 4.431692774 | 1.48E-05 | 3.63E-05 | 2.077662104 |
| IGFBP1 | -1.07802887 | 7.815113122 | -4.422023225 | 1.54E-05 | 3.77E-05 | 2.038362473 |
| TAT | -1.220749959 | 6.695067873 | -4.379098638 | 1.84E-05 | 4.48E-05 | 1.864773026 |
| FGF21 | 1.094702174 | 3.202533937 | 4.349707299 | 2.09E-05 | 5.03E-05 | 1.74673221 |
| IL8 | -1.007209225 | 2.782895928 | -4.334076951 | 2.23E-05 | 5.35E-05 | 1.684230199 |
| APOA4 | -1.190016592 | 2.171764706 | -4.318051488 | 2.38E-05 | 5.70E-05 | 1.620344934 |
| CPS1 | -1.046187987 | 8.004977376 | -4.251537197 | 3.14E-05 | 7.40E-05 | 1.357323704 |
| GSTA2 | -1.226927991 | 5.376425339 | -4.241048782 | 3.28E-05 | 7.71E-05 | 1.316164266 |
| HSD11B1 | -1.108760577 | 6.97800905 | -3.952716458 | 0.000104167 | 0.000230711 | 0.218791533 |
| SAA2 | -1.258074498 | 5.131131222 | -3.571138709 | 0.000436568 | 0.000895178 | -1.129716453 |
| SLPI | -1.007227476 | 5.407375566 | -3.292446732 | 0.001157196 | 0.002243603 | -2.037503764 |
| SAA1 | -1.039457442 | 7.19800905 | -2.888802799 | 0.004253883 | 0.007606852 | -3.233019978 |
| MIR3654 | 1.290607267 | 2.848914027 | 2.509865752 | 0.012798281 | 0.021195199 | -4.223102005 |

DEGs, differentially expressed genes.
