# Supplementary material for: Integrative analysis identifies key mRNA biomarkers for diagnosis, prognosis, and therapeutic targets of HCV-associated hepatocellular carcinoma
Source: Aging (Albany NY). 2021 May 4;13(9):12865–95. doi: 10.18632/aging.202957 (PMC8148482; doi:10.18632/aging.202957)
Supplement: Supplementary Table 4 [file aging-13-202957-s005.pdf]

Supplementary Table 4. Putative upstream regulatory transcription factors of the hub genes.

ID	Accession	Target	TargetID	Experiment	Literature	Tissue
ATF1	466	TOP2A	7153	Unknown	12104051	Not Applicable
BRCA1	672	ASPM	259266	+	16123590	Not Applicable
BRCA1	672	CCNB1	891	Unknown	12647291	Not Applicable
CUX1	1523	RACGAP1	29127	Unknown	21886810	Not Applicable
E2F1	1869	AURKA	6790	+	20300951	Not Applicable
E2F1	1869	CCNB1	891	Unknown	22508987	Not Applicable
E2F1	1869	RACGAP1	29127	Unknown	21886810	Not Applicable
E2F1	1869	TOP2A	7153	+	11313881	Not Applicable
E2F3	1871	AURKA	6790	+	18776222	Not Applicable
E2F3	1871	CCNB1	891	Unknown	17098936	Not Applicable
E2F4	1874	CCNB1	891	Unknown	22508987	Not Applicable
FOXJ1	2302	ASPM	259266	Unknown	16809635	Not Applicable
FOXM1	2305	CCNB1	891	+	19276163	Not Applicable
IRF1	3659	CCNB1	891	-	22200613	Not Applicable
KLF4	9314	CCNB1	891	-	14627709	Not Applicable
KLF5	688	CCNB1	891	+	21951574	Not Applicable
MED1	5469	AURKA	6790	+	16574658	Not Applicable
MYC	4609	CCNB1	891	Unknown	11522645	Not Applicable
NFKB1	4790	CCNB1	891	+	11861406	Not Applicable
NFKB1	4790	CCNB1	891	Unknown	19610058	Not Applicable
OTX2	5015	AURKA	6790	+	21047732	Not Applicable
PTTG1	9232	CCNB1	891	+	22475756	Not Applicable
RELA	5970	CCNB1	891	+	11861406	Not Applicable
RELA	5970	CCNB1	891	Unknown	19610058	Not Applicable
TBP	6908	CCNB1	891	Unknown	17098936	Not Applicable
TFAP2A	7020	CCNB1	891	Unknown	7739559	Not Applicable
TP53	7157	CCNB1	891	-	11892838	Not Applicable
TP53	7157	PRC1	9055	-	12889596	Not Applicable
UHRF1	29128	TOP2A	7153	Unknown	10646863	Not Applicable
USF1	7391	CCNB1	891	Unknown	10548544	Not Applicable
YBX1	4904	CCNB1	891	-	20596676	Not Applicable
YBX1	4904	CDKN3	1033	+	20596676	Not Applicable
YBX1	4904	TOP2A	7153	Unknown	10597187	Not Applicable
