# Supplementary material for: Integrative analysis identifies key mRNA biomarkers for diagnosis, prognosis, and therapeutic targets of HCV-associated hepatocellular carcinoma
Source: Aging (Albany NY). 2021 May 4;13(9):12865–95. doi: 10.18632/aging.202957 (PMC8148482; doi:10.18632/aging.202957)
Supplement: Supplementary Table 5 [file aging-13-202957-s006.doc]

## Supplementary Table 5. putative miRNA-mRNA pairs by experiments based on miRTarbase 8.0.

| **miRNA** | **Species (miRNA)** | **Target Gene** | **Species (Target Gene)** | **Experiments** | **Support Type** | **References (PMID)** |
| --- | --- | --- | --- | --- | --- | --- |
| hsa-miR-373-3p | Homo sapiens | CENPF | Homo sapiens | Microarray//Microarray; Other | Functional MTI (Weak) | 15685193 |
| hsa-miR-373-3p | Homo sapiens | PRC1 | Homo sapiens | Microarray//Microarray; Other | Functional MTI (Weak) | 15685193 |
| hsa-miR-32-5p | Homo sapiens | AURKA | Homo sapiens | Luciferase reporter assay//qRT-PCR//Western blot | Functional MTI | 26036635 |
| hsa-let-7b-5p | Homo sapiens | AURKA | Homo sapiens | Microarray | Functional MTI (Weak) | 17699775 |
| hsa-let-7b-5p | Homo sapiens | AURKA | Homo sapiens | Proteomics | Functional MTI (Weak) | 18668040 |
| hsa-miR-129-1-3p | Homo sapiens | AURKA | Homo sapiens | Immunoprecipitaion//Luciferase reporter assay//qRT-PCR//Western blot | Functional MTI | 27793005 |
| hsa-miR-548b-3p | Homo sapiens | PRC1 | Homo sapiens | Sequencing | Functional MTI (Weak) | 20371350 |
| hsa-miR-193b-3p | Homo sapiens | RACGAP1 | Homo sapiens | Microarray | Functional MTI (Weak) | 20304954 |
| hsa-miR-193b-3p | Homo sapiens | TOP2A | Homo sapiens | Microarray | Functional MTI (Weak) | 20304954 |
| hsa-miR-193b-3p | Homo sapiens | ASPM | Homo sapiens | Microarray | Functional MTI (Weak) | 20304954 |
| hsa-miR-375 | Homo sapiens | CENPF | Homo sapiens | Microarray | Functional MTI (Weak) | 20215506 |
| hsa-miR-155-5p | Homo sapiens | AURKA | Homo sapiens | Proteomics | Functional MTI (Weak) | 18668040 |
| hsa-miR-132-3p | Homo sapiens | CCNB1 | Homo sapiens | Western blot; qRT-PCR | Functional MTI | 21329664 |
| hsa-miR-128-3p | Homo sapiens | NEK2 | Homo sapiens | Microarray | Functional MTI (Weak) | 17612493 |
| hsa-miR-212-3p | Homo sapiens | CCNB1 | Homo sapiens | Western blot; qRT-PCR | Functional MTI | 21329664 |
| hsa-miR-124-3p | Homo sapiens | AURKA | Homo sapiens | Proteomics; Microarray | Functional MTI (Weak) | 18668037 |
| hsa-miR-122-5p | Homo sapiens | CENPF | Homo sapiens | Proteomics | Functional MTI (Weak) | 21750653 |
| hsa-miR-1-3p | Homo sapiens | CENPF | Homo sapiens | Proteomics | Functional MTI (Weak) | 18668040 |
| hsa-miR-215-5p | Homo sapiens | RACGAP1 | Homo sapiens | Microarray | Functional MTI (Weak) | 19074876 |
| hsa-miR-215-5p | Homo sapiens | CDKN3 | Homo sapiens | Microarray | Functional MTI (Weak) | 19074876 |
| hsa-miR-215-5p | Homo sapiens | CENPF | Homo sapiens | Microarray | Functional MTI (Weak) | 19074876 |
| hsa-miR-215-5p | Homo sapiens | NUF2 | Homo sapiens | Microarray | Functional MTI (Weak) | 19074876 |
| hsa-miR-215-5p | Homo sapiens | ASPM | Homo sapiens | Microarray | Functional MTI (Weak) | 19074876 |
| hsa-miR-548b-3p | Homo sapiens | CCNB1 | Homo sapiens | Luciferase reporter assay//Immunofluorescence analysis//qRT-PCR | Functional MTI | 24832599 |
| hsa-miR-192-5p | Homo sapiens | NUF2 | Homo sapiens | Microarray | Functional MTI (Weak) | 19074876 |
| hsa-miR-192-5p | Homo sapiens | ASPM | Homo sapiens | Microarray | Functional MTI (Weak) | 19074876 |
| hsa-miR-192-5p | Homo sapiens | CENPF | Homo sapiens | Microarray | Functional MTI (Weak) | 19074876 |
| hsa-miR-192-5p | Homo sapiens | CDKN3 | Homo sapiens | Microarray | Functional MTI (Weak) | 19074876 |
| hsa-miR-26b-5p | Homo sapiens | RACGAP1 | Homo sapiens | Microarray | Functional MTI (Weak) | 19088304 |
| hsa-miR-26b-5p | Homo sapiens | ASPM | Homo sapiens | Microarray | Functional MTI (Weak) | 19088304 |
| hsa-miR-24-3p | Homo sapiens | CCNB1 | Homo sapiens | Microarray | Functional MTI (Weak) | 19748357 |
| hsa-miR-21-5p | Homo sapiens | TOP2A | Homo sapiens | Microarray | Functional MTI (Weak) | 18591254 |
| hsa-miR-16-5p | Homo sapiens | RACGAP1 | Homo sapiens | Proteomics | Functional MTI (Weak) | 18668040 |
| hsa-miR-16-5p | Homo sapiens | RACGAP1 | Homo sapiens | PAR-CLIP | Functional MTI (Weak) | 21572407 |
| hsa-miR-16-5p | Homo sapiens | CENPF | Homo sapiens | Proteomics | Functional MTI (Weak) | 18668040 |
| hsa-miR-1226-3p | Homo sapiens | TOP2A | Homo sapiens | CLASH | Functional MTI (Weak) | 23622248 |
| hsa-miR-1226-3p | Homo sapiens | CENPF | Homo sapiens | CLASH | Functional MTI (Weak) | 23622248 |
| hsa-miR-877-5p | Homo sapiens | CENPF | Homo sapiens | CLASH | Functional MTI (Weak) | 23622248 |
| hsa-miR-708-5p | Homo sapiens | TOP2A | Homo sapiens | CLASH | Functional MTI (Weak) | 23622248 |
| hsa-miR-93-3p | Homo sapiens | CENPF | Homo sapiens | CLASH | Functional MTI (Weak) | 23622248 |
| hsa-miR-505-3p | Homo sapiens | PRC1 | Homo sapiens | CLASH | Functional MTI (Weak) | 23622248 |
| hsa-miR-193a-3p | Homo sapiens | AURKA | Homo sapiens | CLASH | Functional MTI (Weak) | 23622248 |
| hsa-miR-186-5p | Homo sapiens | TOP2A | Homo sapiens | CLASH | Functional MTI (Weak) | 23622248 |
| hsa-miR-125a-5p | Homo sapiens | PRC1 | Homo sapiens | CLASH | Functional MTI (Weak) | 23622248 |
| hsa-miR-221-3p | Homo sapiens | NUF2 | Homo sapiens | CLASH | Functional MTI (Weak) | 23622248 |
| hsa-miR-221-3p | Homo sapiens | RACGAP1 | Homo sapiens | CLASH | Functional MTI (Weak) | 23622248 |
| hsa-miR-183-5p | Homo sapiens | CCNB1 | Homo sapiens | CLASH | Functional MTI (Weak) | 23622248 |
| hsa-miR-183-5p | Homo sapiens | CCNB1 | Homo sapiens | PAR-CLIP | Functional MTI (Weak) | 20371350 |
| hsa-miR-7-5p | Homo sapiens | TOP2A | Homo sapiens | CLASH | Functional MTI (Weak) | 23622248 |
| hsa-miR-92a-3p | Homo sapiens | NEK2 | Homo sapiens | CLASH | Functional MTI (Weak) | 23622248 |
| hsa-miR-92a-3p | Homo sapiens | CDKN3 | Homo sapiens | CLASH | Functional MTI (Weak) | 23622248 |
| hsa-miR-92a-3p | Homo sapiens | CCNB1 | Homo sapiens | CLASH | Functional MTI (Weak) | 23622248 |
| hsa-miR-92a-3p | Homo sapiens | CCNB1 | Homo sapiens | PAR-CLIP | Functional MTI (Weak) | 26701625 |
| hsa-miR-92a-3p | Homo sapiens | PRC1 | Homo sapiens | CLASH | Functional MTI (Weak) | 23622248 |
| hsa-miR-20a-5p | Homo sapiens | CCNB1 | Homo sapiens | CLASH | Functional MTI (Weak) | 23622248 |
| hsa-let-7e-5p | Homo sapiens | RACGAP1 | Homo sapiens | CLASH | Functional MTI (Weak) | 23622248 |
| hsa-let-7b-5p | Homo sapiens | CCNB1 | Homo sapiens | CLASH | Functional MTI (Weak) | 23622248 |
| hsa-let-7a-5p | Homo sapiens | ASPM | Homo sapiens | CLASH | Functional MTI (Weak) | 23622248 |
| hsa-miR-1260b | Homo sapiens | PRC1 | Homo sapiens | CLASH | Functional MTI (Weak) | 23622248 |
| hsa-miR-410-3p | Homo sapiens | CCNB1 | Homo sapiens | Luciferase reporter assay//qRT-PCR | Functional MTI | 26125663 |
| hsa-miR-559 | Homo sapiens | CCNB1 | Homo sapiens | PAR-CLIP | Functional MTI (Weak) | 22012620 |
| hsa-miR-559 | Homo sapiens | CCNB1 | Homo sapiens | PAR-CLIP | Functional MTI (Weak) | 20371350 |
| hsa-miR-548b-5p | Homo sapiens | CCNB1 | Homo sapiens | PAR-CLIP | Functional MTI (Weak) | 22012620 |
| hsa-miR-548b-5p | Homo sapiens | CCNB1 | Homo sapiens | PAR-CLIP | Functional MTI (Weak) | 20371350 |
| hsa-miR-548a-5p | Homo sapiens | CCNB1 | Homo sapiens | PAR-CLIP | Functional MTI (Weak) | 22012620 |
| hsa-miR-548a-5p | Homo sapiens | CCNB1 | Homo sapiens | PAR-CLIP | Functional MTI (Weak) | 20371350 |
| hsa-miR-548c-5p | Homo sapiens | CCNB1 | Homo sapiens | PAR-CLIP | Functional MTI (Weak) | 22012620 |
| hsa-miR-548c-5p | Homo sapiens | CCNB1 | Homo sapiens | PAR-CLIP | Functional MTI (Weak) | 20371350 |
| hsa-miR-548d-5p | Homo sapiens | CCNB1 | Homo sapiens | PAR-CLIP | Functional MTI (Weak) | 22012620 |
| hsa-miR-548d-5p | Homo sapiens | CCNB1 | Homo sapiens | PAR-CLIP | Functional MTI (Weak) | 20371350 |
| hsa-miR-548j-5p | Homo sapiens | CCNB1 | Homo sapiens | PAR-CLIP | Functional MTI (Weak) | 22012620 |
| hsa-miR-548j-5p | Homo sapiens | CCNB1 | Homo sapiens | PAR-CLIP | Functional MTI (Weak) | 20371350 |
| hsa-miR-548n | Homo sapiens | CCNB1 | Homo sapiens | PAR-CLIP | Functional MTI (Weak) | 22012620 |
| hsa-miR-548n | Homo sapiens | CCNB1 | Homo sapiens | PAR-CLIP | Functional MTI (Weak) | 20371350 |
| hsa-miR-548h-5p | Homo sapiens | CCNB1 | Homo sapiens | PAR-CLIP | Functional MTI (Weak) | 22012620 |
| hsa-miR-548h-5p | Homo sapiens | CCNB1 | Homo sapiens | PAR-CLIP | Functional MTI (Weak) | 20371350 |
| hsa-miR-548i | Homo sapiens | CCNB1 | Homo sapiens | PAR-CLIP | Functional MTI (Weak) | 22012620 |
| hsa-miR-548i | Homo sapiens | CCNB1 | Homo sapiens | PAR-CLIP | Functional MTI (Weak) | 20371350 |
| hsa-miR-548w | Homo sapiens | CCNB1 | Homo sapiens | PAR-CLIP | Functional MTI (Weak) | 22012620 |
| hsa-miR-548w | Homo sapiens | CCNB1 | Homo sapiens | PAR-CLIP | Functional MTI (Weak) | 20371350 |
| hsa-miR-548y | Homo sapiens | CCNB1 | Homo sapiens | PAR-CLIP | Functional MTI (Weak) | 22012620 |
| hsa-miR-548y | Homo sapiens | CCNB1 | Homo sapiens | PAR-CLIP | Functional MTI (Weak) | 20371350 |
| hsa-miR-548o-5p | Homo sapiens | CCNB1 | Homo sapiens | PAR-CLIP | Functional MTI (Weak) | 22012620 |
| hsa-miR-548o-5p | Homo sapiens | CCNB1 | Homo sapiens | PAR-CLIP | Functional MTI (Weak) | 20371350 |
| hsa-miR-548ab | Homo sapiens | CCNB1 | Homo sapiens | PAR-CLIP | Functional MTI (Weak) | 22012620 |
| hsa-miR-548ab | Homo sapiens | CCNB1 | Homo sapiens | PAR-CLIP | Functional MTI (Weak) | 20371350 |
| hsa-miR-548ad-5p | Homo sapiens | CCNB1 | Homo sapiens | PAR-CLIP | Functional MTI (Weak) | 22012620 |
| hsa-miR-548ad-5p | Homo sapiens | CCNB1 | Homo sapiens | PAR-CLIP | Functional MTI (Weak) | 20371350 |
| hsa-miR-548ae-5p | Homo sapiens | CCNB1 | Homo sapiens | PAR-CLIP | Functional MTI (Weak) | 22012620 |
| hsa-miR-548ae-5p | Homo sapiens | CCNB1 | Homo sapiens | PAR-CLIP | Functional MTI (Weak) | 20371350 |
| hsa-miR-548ak | Homo sapiens | CCNB1 | Homo sapiens | PAR-CLIP | Functional MTI (Weak) | 22012620 |
| hsa-miR-548ak | Homo sapiens | CCNB1 | Homo sapiens | PAR-CLIP | Functional MTI (Weak) | 20371350 |
| hsa-miR-4517 | Homo sapiens | CCNB1 | Homo sapiens | PAR-CLIP | Functional MTI (Weak) | 22012620 |
| hsa-miR-4517 | Homo sapiens | CCNB1 | Homo sapiens | PAR-CLIP | Functional MTI (Weak) | 20371350 |
| hsa-miR-548am-5p | Homo sapiens | CCNB1 | Homo sapiens | PAR-CLIP | Functional MTI (Weak) | 22012620 |
| hsa-miR-548am-5p | Homo sapiens | CCNB1 | Homo sapiens | PAR-CLIP | Functional MTI (Weak) | 20371350 |
| hsa-miR-4709-5p | Homo sapiens | CCNB1 | Homo sapiens | PAR-CLIP | Functional MTI (Weak) | 22012620 |
| hsa-miR-4709-5p | Homo sapiens | CCNB1 | Homo sapiens | PAR-CLIP | Functional MTI (Weak) | 20371350 |
| hsa-miR-548ap-5p | Homo sapiens | CCNB1 | Homo sapiens | PAR-CLIP | Functional MTI (Weak) | 22012620 |
| hsa-miR-548ap-5p | Homo sapiens | CCNB1 | Homo sapiens | PAR-CLIP | Functional MTI (Weak) | 20371350 |
| hsa-miR-548aq-5p | Homo sapiens | CCNB1 | Homo sapiens | PAR-CLIP | Functional MTI (Weak) | 22012620 |
| hsa-miR-548aq-5p | Homo sapiens | CCNB1 | Homo sapiens | PAR-CLIP | Functional MTI (Weak) | 20371350 |
| hsa-miR-548ar-5p | Homo sapiens | CCNB1 | Homo sapiens | PAR-CLIP | Functional MTI (Weak) | 22012620 |
| hsa-miR-548ar-5p | Homo sapiens | CCNB1 | Homo sapiens | PAR-CLIP | Functional MTI (Weak) | 20371350 |
| hsa-miR-548as-5p | Homo sapiens | CCNB1 | Homo sapiens | PAR-CLIP | Functional MTI (Weak) | 22012620 |
| hsa-miR-548as-5p | Homo sapiens | CCNB1 | Homo sapiens | PAR-CLIP | Functional MTI (Weak) | 20371350 |
| hsa-miR-5583-3p | Homo sapiens | CCNB1 | Homo sapiens | PAR-CLIP | Functional MTI (Weak) | 22012620 |
| hsa-miR-5583-3p | Homo sapiens | CCNB1 | Homo sapiens | PAR-CLIP | Functional MTI (Weak) | 20371350 |
| hsa-miR-548au-5p | Homo sapiens | CCNB1 | Homo sapiens | PAR-CLIP | Functional MTI (Weak) | 22012620 |
| hsa-miR-548au-5p | Homo sapiens | CCNB1 | Homo sapiens | PAR-CLIP | Functional MTI (Weak) | 20371350 |
| hsa-miR-548ay-5p | Homo sapiens | CCNB1 | Homo sapiens | PAR-CLIP | Functional MTI (Weak) | 22012620 |
| hsa-miR-548ay-5p | Homo sapiens | CCNB1 | Homo sapiens | PAR-CLIP | Functional MTI (Weak) | 20371350 |
| hsa-miR-548bb-5p | Homo sapiens | CCNB1 | Homo sapiens | PAR-CLIP | Functional MTI (Weak) | 22012620 |
| hsa-miR-548bb-5p | Homo sapiens | CCNB1 | Homo sapiens | PAR-CLIP | Functional MTI (Weak) | 20371350 |
| hsa-miR-3918 | Homo sapiens | AURKA | Homo sapiens | PAR-CLIP | Functional MTI (Weak) | 21572407 |
| hsa-miR-590-3p | Homo sapiens | CCNB1 | Homo sapiens | PAR-CLIP | Functional MTI (Weak) | 20371350 |
| hsa-miR-3145-5p | Homo sapiens | CCNB1 | Homo sapiens | PAR-CLIP | Functional MTI (Weak) | 20371350 |
| hsa-miR-3163 | Homo sapiens | CCNB1 | Homo sapiens | PAR-CLIP | Functional MTI (Weak) | 20371350 |
| hsa-miR-208a-5p | Homo sapiens | RACGAP1 | Homo sapiens | PAR-CLIP | Functional MTI (Weak) | 20371350 |
| hsa-miR-576-3p | Homo sapiens | RACGAP1 | Homo sapiens | PAR-CLIP | Functional MTI (Weak) | 20371350 |
| hsa-miR-208b-5p | Homo sapiens | RACGAP1 | Homo sapiens | PAR-CLIP | Functional MTI (Weak) | 20371350 |
| hsa-miR-590-3p | Homo sapiens | RACGAP1 | Homo sapiens | PAR-CLIP | Functional MTI (Weak) | 20371350 |
| hsa-let-7a-2-3p | Homo sapiens | CCNB1 | Homo sapiens | PAR-CLIP | Functional MTI (Weak) | 22012620 |
| hsa-let-7a-2-3p | Homo sapiens | CCNB1 | Homo sapiens | PAR-CLIP | Functional MTI (Weak) | 20371350 |
| hsa-miR-192-3p | Homo sapiens | CCNB1 | Homo sapiens | PAR-CLIP | Functional MTI (Weak) | 20371350 |
| hsa-let-7g-3p | Homo sapiens | CCNB1 | Homo sapiens | PAR-CLIP | Functional MTI (Weak) | 22012620 |
| hsa-let-7g-3p | Homo sapiens | CCNB1 | Homo sapiens | PAR-CLIP | Functional MTI (Weak) | 20371350 |
| hsa-miR-5589-3p | Homo sapiens | CCNB1 | Homo sapiens | PAR-CLIP | Functional MTI (Weak) | 20371350 |
| hsa-miR-5683 | Homo sapiens | CCNB1 | Homo sapiens | PAR-CLIP | Functional MTI (Weak) | 22012620 |
| hsa-miR-5683 | Homo sapiens | CCNB1 | Homo sapiens | PAR-CLIP | Functional MTI (Weak) | 20371350 |
| hsa-miR-6762-5p | Homo sapiens | CCNB1 | Homo sapiens | PAR-CLIP | Functional MTI (Weak) | 20371350 |
| hsa-miR-6845-5p | Homo sapiens | CCNB1 | Homo sapiens | PAR-CLIP | Functional MTI (Weak) | 20371350 |
| hsa-miR-484 | Homo sapiens | RACGAP1 | Homo sapiens | HITS-CLIP | Functional MTI (Weak) | 23824327 |
| hsa-miR-3119 | Homo sapiens | RACGAP1 | Homo sapiens | PAR-CLIP | Functional MTI (Weak) | 21572407 |
| hsa-miR-3155a | Homo sapiens | RACGAP1 | Homo sapiens | HITS-CLIP | Functional MTI (Weak) | 23824327 |
| hsa-miR-3155b | Homo sapiens | RACGAP1 | Homo sapiens | HITS-CLIP | Functional MTI (Weak) | 23824327 |
| hsa-miR-218-5p | Homo sapiens | TOP2A | Homo sapiens | HITS-CLIP | Functional MTI (Weak) | 23212916 |
| hsa-miR-218-5p | Homo sapiens | ASPM | Homo sapiens | HITS-CLIP | Functional MTI (Weak) | 23212916 |
| hsa-miR-5006-3p | Homo sapiens | RACGAP1 | Homo sapiens | PAR-CLIP | Functional MTI (Weak) | 23592263 |
| hsa-miR-5006-3p | Homo sapiens | RACGAP1 | Homo sapiens | PAR-CLIP | Functional MTI (Weak) | 23446348 |
| hsa-miR-5006-3p | Homo sapiens | RACGAP1 | Homo sapiens | PAR-CLIP | Functional MTI (Weak) | 21572407 |
| hsa-miR-5006-3p | Homo sapiens | RACGAP1 | Homo sapiens | PAR-CLIP | Functional MTI (Weak) | 20371350 |
| hsa-miR-4755-5p | Homo sapiens | RACGAP1 | Homo sapiens | PAR-CLIP | Functional MTI (Weak) | 23592263 |
| hsa-miR-4755-5p | Homo sapiens | RACGAP1 | Homo sapiens | PAR-CLIP | Functional MTI (Weak) | 23446348 |
| hsa-miR-4755-5p | Homo sapiens | RACGAP1 | Homo sapiens | PAR-CLIP | Functional MTI (Weak) | 21572407 |
| hsa-miR-4755-5p | Homo sapiens | RACGAP1 | Homo sapiens | PAR-CLIP | Functional MTI (Weak) | 20371350 |
| hsa-miR-211-5p | Homo sapiens | RACGAP1 | Homo sapiens | PAR-CLIP | Functional MTI (Weak) | 23592263 |
| hsa-miR-211-5p | Homo sapiens | RACGAP1 | Homo sapiens | PAR-CLIP | Functional MTI (Weak) | 23446348 |
| hsa-miR-211-5p | Homo sapiens | RACGAP1 | Homo sapiens | PAR-CLIP | Functional MTI (Weak) | 21572407 |
| hsa-miR-211-5p | Homo sapiens | RACGAP1 | Homo sapiens | PAR-CLIP | Functional MTI (Weak) | 20371350 |
| hsa-miR-204-5p | Homo sapiens | RACGAP1 | Homo sapiens | PAR-CLIP | Functional MTI (Weak) | 23592263 |
| hsa-miR-204-5p | Homo sapiens | RACGAP1 | Homo sapiens | PAR-CLIP | Functional MTI (Weak) | 23446348 |
| hsa-miR-204-5p | Homo sapiens | RACGAP1 | Homo sapiens | PAR-CLIP | Functional MTI (Weak) | 21572407 |
| hsa-miR-204-5p | Homo sapiens | RACGAP1 | Homo sapiens | PAR-CLIP | Functional MTI (Weak) | 20371350 |
| hsa-miR-623 | Homo sapiens | RACGAP1 | Homo sapiens | PAR-CLIP | Functional MTI (Weak) | 23592263 |
| hsa-miR-623 | Homo sapiens | RACGAP1 | Homo sapiens | PAR-CLIP | Functional MTI (Weak) | 23446348 |
| hsa-miR-623 | Homo sapiens | RACGAP1 | Homo sapiens | PAR-CLIP | Functional MTI (Weak) | 21572407 |
| hsa-miR-623 | Homo sapiens | RACGAP1 | Homo sapiens | PAR-CLIP | Functional MTI (Weak) | 20371350 |
| hsa-miR-24-2-5p | Homo sapiens | RACGAP1 | Homo sapiens | PAR-CLIP | Functional MTI (Weak) | 23592263 |
| hsa-miR-24-2-5p | Homo sapiens | RACGAP1 | Homo sapiens | PAR-CLIP | Functional MTI (Weak) | 23446348 |
| hsa-miR-24-2-5p | Homo sapiens | RACGAP1 | Homo sapiens | PAR-CLIP | Functional MTI (Weak) | 21572407 |
| hsa-miR-24-2-5p | Homo sapiens | RACGAP1 | Homo sapiens | PAR-CLIP | Functional MTI (Weak) | 20371350 |
| hsa-miR-24-1-5p | Homo sapiens | RACGAP1 | Homo sapiens | PAR-CLIP | Functional MTI (Weak) | 23592263 |
| hsa-miR-24-1-5p | Homo sapiens | RACGAP1 | Homo sapiens | PAR-CLIP | Functional MTI (Weak) | 23446348 |
| hsa-miR-24-1-5p | Homo sapiens | RACGAP1 | Homo sapiens | PAR-CLIP | Functional MTI (Weak) | 21572407 |
| hsa-miR-24-1-5p | Homo sapiens | RACGAP1 | Homo sapiens | PAR-CLIP | Functional MTI (Weak) | 20371350 |
| hsa-miR-141-5p | Homo sapiens | RACGAP1 | Homo sapiens | PAR-CLIP | Functional MTI (Weak) | 23592263 |
| hsa-miR-141-5p | Homo sapiens | RACGAP1 | Homo sapiens | PAR-CLIP | Functional MTI (Weak) | 23446348 |
| hsa-miR-141-5p | Homo sapiens | RACGAP1 | Homo sapiens | PAR-CLIP | Functional MTI (Weak) | 21572407 |
| hsa-miR-141-5p | Homo sapiens | RACGAP1 | Homo sapiens | PAR-CLIP | Functional MTI (Weak) | 20371350 |
| hsa-miR-3651 | Homo sapiens | RACGAP1 | Homo sapiens | PAR-CLIP | Functional MTI (Weak) | 23592263 |
| hsa-miR-3651 | Homo sapiens | RACGAP1 | Homo sapiens | PAR-CLIP | Functional MTI (Weak) | 23446348 |
| hsa-miR-3651 | Homo sapiens | RACGAP1 | Homo sapiens | PAR-CLIP | Functional MTI (Weak) | 21572407 |
| hsa-miR-3651 | Homo sapiens | RACGAP1 | Homo sapiens | PAR-CLIP | Functional MTI (Weak) | 20371350 |
| hsa-miR-140-3p | Homo sapiens | RACGAP1 | Homo sapiens | PAR-CLIP | Functional MTI (Weak) | 23592263 |
| hsa-miR-140-3p | Homo sapiens | RACGAP1 | Homo sapiens | PAR-CLIP | Functional MTI (Weak) | 23446348 |
| hsa-miR-140-3p | Homo sapiens | RACGAP1 | Homo sapiens | PAR-CLIP | Functional MTI (Weak) | 21572407 |
| hsa-miR-140-3p | Homo sapiens | RACGAP1 | Homo sapiens | PAR-CLIP | Functional MTI (Weak) | 20371350 |
| hsa-miR-4477a | Homo sapiens | CCNB1 | Homo sapiens | PAR-CLIP | Functional MTI (Weak) | 22012620 |
| hsa-miR-4477a | Homo sapiens | CCNB1 | Homo sapiens | PAR-CLIP | Functional MTI (Weak) | 20371350 |
| hsa-miR-548t-5p | Homo sapiens | CCNB1 | Homo sapiens | PAR-CLIP | Functional MTI (Weak) | 22012620 |
| hsa-miR-548az-5p | Homo sapiens | CCNB1 | Homo sapiens | PAR-CLIP | Functional MTI (Weak) | 22012620 |
| hsa-miR-3127-5p | Homo sapiens | AURKA | Homo sapiens | PAR-CLIP | Functional MTI (Weak) | 21572407 |
| hsa-miR-137 | Homo sapiens | AURKA | Homo sapiens | PAR-CLIP | Functional MTI (Weak) | 21572407 |
| hsa-miR-4448 | Homo sapiens | AURKA | Homo sapiens | PAR-CLIP | Functional MTI (Weak) | 21572407 |
| hsa-miR-6736-3p | Homo sapiens | AURKA | Homo sapiens | PAR-CLIP | Functional MTI (Weak) | 21572407 |
| hsa-miR-154-5p | Homo sapiens | AURKA | Homo sapiens | PAR-CLIP | Functional MTI (Weak) | 21572407 |
| hsa-miR-7843-5p | Homo sapiens | AURKA | Homo sapiens | PAR-CLIP | Functional MTI (Weak) | 21572407 |
| hsa-miR-6735-5p | Homo sapiens | AURKA | Homo sapiens | PAR-CLIP | Functional MTI (Weak) | 21572407 |
| hsa-miR-6879-5p | Homo sapiens | AURKA | Homo sapiens | PAR-CLIP | Functional MTI (Weak) | 21572407 |
| hsa-miR-4632-5p | Homo sapiens | AURKA | Homo sapiens | PAR-CLIP | Functional MTI (Weak) | 21572407 |
| hsa-miR-4436b-3p | Homo sapiens | AURKA | Homo sapiens | PAR-CLIP | Functional MTI (Weak) | 21572407 |
| hsa-miR-6876-5p | Homo sapiens | AURKA | Homo sapiens | PAR-CLIP | Functional MTI (Weak) | 21572407 |
| hsa-miR-4476 | Homo sapiens | AURKA | Homo sapiens | PAR-CLIP | Functional MTI (Weak) | 21572407 |
| hsa-miR-367-3p | Homo sapiens | AURKA | Homo sapiens | PAR-CLIP | Functional MTI (Weak) | 21572407 |
| hsa-miR-363-3p | Homo sapiens | AURKA | Homo sapiens | PAR-CLIP | Functional MTI (Weak) | 21572407 |
| hsa-miR-92b-3p | Homo sapiens | AURKA | Homo sapiens | HITS-CLIP | Functional MTI (Weak) | 22473208 |
| hsa-miR-92b-3p | Homo sapiens | AURKA | Homo sapiens | PAR-CLIP | Functional MTI (Weak) | 21572407 |
| hsa-miR-25-3p | Homo sapiens | AURKA | Homo sapiens | PAR-CLIP | Functional MTI (Weak) | 21572407 |
| hsa-miR-32-5p | Homo sapiens | AURKA | Homo sapiens | HITS-CLIP | Functional MTI (Weak) | 22473208 |
| hsa-miR-32-5p | Homo sapiens | AURKA | Homo sapiens | PAR-CLIP | Functional MTI (Weak) | 21572407 |
| hsa-miR-146a-5p | Homo sapiens | CDKN3 | Homo sapiens | Western blot | Non-Functional MTI | 19944095 |
| hsa-miR-92a-3p | Homo sapiens | AURKA | Homo sapiens | HITS-CLIP | Functional MTI (Weak) | 22473208 |
| hsa-miR-92a-3p | Homo sapiens | AURKA | Homo sapiens | PAR-CLIP | Functional MTI (Weak) | 21572407 |
| hsa-miR-7159-3p | Homo sapiens | RACGAP1 | Homo sapiens | PAR-CLIP | Functional MTI (Weak) | 21572407 |
| hsa-miR-4503 | Homo sapiens | RACGAP1 | Homo sapiens | PAR-CLIP | Functional MTI (Weak) | 21572407 |
| hsa-miR-107 | Homo sapiens | RACGAP1 | Homo sapiens | PAR-CLIP | Functional MTI (Weak) | 21572407 |
| hsa-miR-103a-3p | Homo sapiens | RACGAP1 | Homo sapiens | PAR-CLIP | Functional MTI (Weak) | 21572407 |
| hsa-miR-7157-5p | Homo sapiens | RACGAP1 | Homo sapiens | PAR-CLIP | Functional MTI (Weak) | 21572407 |
| hsa-miR-4310 | Homo sapiens | RACGAP1 | Homo sapiens | PAR-CLIP | Functional MTI (Weak) | 21572407 |
| hsa-miR-556-5p | Homo sapiens | RACGAP1 | Homo sapiens | PAR-CLIP | Functional MTI (Weak) | 21572407 |
| hsa-miR-382-3p | Homo sapiens | RACGAP1 | Homo sapiens | PAR-CLIP | Functional MTI (Weak) | 21572407 |
| hsa-miR-6838-5p | Homo sapiens | RACGAP1 | Homo sapiens | PAR-CLIP | Functional MTI (Weak) | 21572407 |
| hsa-miR-497-5p | Homo sapiens | RACGAP1 | Homo sapiens | PAR-CLIP | Functional MTI (Weak) | 21572407 |
| hsa-miR-424-5p | Homo sapiens | RACGAP1 | Homo sapiens | PAR-CLIP | Functional MTI (Weak) | 21572407 |
| hsa-miR-195-5p | Homo sapiens | RACGAP1 | Homo sapiens | PAR-CLIP | Functional MTI (Weak) | 21572407 |
| hsa-miR-15b-5p | Homo sapiens | RACGAP1 | Homo sapiens | PAR-CLIP | Functional MTI (Weak) | 21572407 |
| hsa-miR-15a-5p | Homo sapiens | RACGAP1 | Homo sapiens | PAR-CLIP | Functional MTI (Weak) | 21572407 |
| hsa-miR-7152-5p | Homo sapiens | AURKA | Homo sapiens | PAR-CLIP | Functional MTI (Weak) | 21572407 |
| hsa-miR-4326 | Homo sapiens | AURKA | Homo sapiens | PAR-CLIP | Functional MTI (Weak) | 21572407 |
| hsa-miR-24-3p | Homo sapiens | AURKA | Homo sapiens | PAR-CLIP | Functional MTI (Weak) | 21572407 |
| hsa-miR-4284 | Homo sapiens | AURKA | Homo sapiens | PAR-CLIP | Functional MTI (Weak) | 21572407 |
| hsa-miR-885-3p | Homo sapiens | AURKA | Homo sapiens | PAR-CLIP | Functional MTI (Weak) | 21572407 |
| hsa-miR-149-5p | Homo sapiens | AURKA | Homo sapiens | PAR-CLIP | Functional MTI (Weak) | 21572407 |
| hsa-miR-5699-3p | Homo sapiens | AURKA | Homo sapiens | PAR-CLIP | Functional MTI (Weak) | 21572407 |
| hsa-miR-4421 | Homo sapiens | AURKA | Homo sapiens | PAR-CLIP | Functional MTI (Weak) | 21572407 |
| hsa-miR-6748-3p | Homo sapiens | AURKA | Homo sapiens | PAR-CLIP | Functional MTI (Weak) | 21572407 |
| hsa-miR-183-5p | Homo sapiens | AURKA | Homo sapiens | PAR-CLIP | Functional MTI (Weak) | 21572407 |
| hsa-miR-8076 | Homo sapiens | AURKA | Homo sapiens | PAR-CLIP | Functional MTI (Weak) | 21572407 |
| hsa-miR-6779-3p | Homo sapiens | AURKA | Homo sapiens | PAR-CLIP | Functional MTI (Weak) | 21572407 |
| hsa-miR-421 | Homo sapiens | RACGAP1 | Homo sapiens | PAR-CLIP | Functional MTI (Weak) | 20371350 |
| hsa-miR-552-5p | Homo sapiens | RACGAP1 | Homo sapiens | PAR-CLIP | Functional MTI (Weak) | 20371350 |
| hsa-miR-3658 | Homo sapiens | RACGAP1 | Homo sapiens | PAR-CLIP | Functional MTI (Weak) | 20371350 |
| hsa-miR-302c-5p | Homo sapiens | RACGAP1 | Homo sapiens | PAR-CLIP | Functional MTI (Weak) | 20371350 |
| hsa-miR-4775 | Homo sapiens | RACGAP1 | Homo sapiens | PAR-CLIP | Functional MTI (Weak) | 20371350 |
| hsa-miR-3653-3p | Homo sapiens | RACGAP1 | Homo sapiens | PAR-CLIP | Functional MTI (Weak) | 20371350 |
| hsa-miR-6507-5p | Homo sapiens | RACGAP1 | Homo sapiens | PAR-CLIP | Functional MTI (Weak) | 20371350 |
| hsa-miR-527 | Homo sapiens | RACGAP1 | Homo sapiens | PAR-CLIP | Functional MTI (Weak) | 20371350 |
| hsa-miR-518a-5p | Homo sapiens | RACGAP1 | Homo sapiens | PAR-CLIP | Functional MTI (Weak) | 20371350 |
| hsa-miR-943 | Homo sapiens | RACGAP1 | Homo sapiens | PAR-CLIP | Functional MTI (Weak) | 20371350 |
| hsa-miR-3137 | Homo sapiens | RACGAP1 | Homo sapiens | PAR-CLIP | Functional MTI (Weak) | 20371350 |
| hsa-miR-520h | Homo sapiens | RACGAP1 | Homo sapiens | PAR-CLIP | Functional MTI (Weak) | 20371350 |
| hsa-miR-520g-3p | Homo sapiens | RACGAP1 | Homo sapiens | PAR-CLIP | Functional MTI (Weak) | 20371350 |
| hsa-miR-1183 | Homo sapiens | RACGAP1 | Homo sapiens | PAR-CLIP | Functional MTI (Weak) | 20371350 |
| hsa-miR-519c-3p | Homo sapiens | RACGAP1 | Homo sapiens | PAR-CLIP | Functional MTI (Weak) | 20371350 |
| hsa-miR-519b-3p | Homo sapiens | RACGAP1 | Homo sapiens | PAR-CLIP | Functional MTI (Weak) | 20371350 |
| hsa-miR-519a-3p | Homo sapiens | RACGAP1 | Homo sapiens | PAR-CLIP | Functional MTI (Weak) | 20371350 |
| hsa-miR-3662 | Homo sapiens | RACGAP1 | Homo sapiens | PAR-CLIP | Functional MTI (Weak) | 20371350 |
| hsa-miR-20a-5p | Homo sapiens | RACGAP1 | Homo sapiens | PAR-CLIP | Functional MTI (Weak) | 20371350 |
| hsa-miR-17-5p | Homo sapiens | RACGAP1 | Homo sapiens | PAR-CLIP | Functional MTI (Weak) | 20371350 |
| hsa-miR-519d-3p | Homo sapiens | RACGAP1 | Homo sapiens | PAR-CLIP | Functional MTI (Weak) | 20371350 |
| hsa-miR-106a-5p | Homo sapiens | RACGAP1 | Homo sapiens | PAR-CLIP | Functional MTI (Weak) | 20371350 |
| hsa-miR-93-5p | Homo sapiens | RACGAP1 | Homo sapiens | PAR-CLIP | Functional MTI (Weak) | 20371350 |
| hsa-miR-526b-3p | Homo sapiens | RACGAP1 | Homo sapiens | PAR-CLIP | Functional MTI (Weak) | 20371350 |
| hsa-miR-106b-5p | Homo sapiens | RACGAP1 | Homo sapiens | PAR-CLIP | Functional MTI (Weak) | 20371350 |
| hsa-miR-20b-5p | Homo sapiens | RACGAP1 | Homo sapiens | PAR-CLIP | Functional MTI (Weak) | 20371350 |
| hsa-miR-301b-3p | Homo sapiens | RACGAP1 | Homo sapiens | PAR-CLIP | Functional MTI (Weak) | 20371350 |
| hsa-miR-301a-3p | Homo sapiens | RACGAP1 | Homo sapiens | PAR-CLIP | Functional MTI (Weak) | 20371350 |
| hsa-miR-130a-3p | Homo sapiens | RACGAP1 | Homo sapiens | PAR-CLIP | Functional MTI (Weak) | 20371350 |
| hsa-miR-130b-3p | Homo sapiens | RACGAP1 | Homo sapiens | PAR-CLIP | Functional MTI (Weak) | 20371350 |
| hsa-miR-4295 | Homo sapiens | RACGAP1 | Homo sapiens | PAR-CLIP | Functional MTI (Weak) | 20371350 |
| hsa-miR-454-3p | Homo sapiens | RACGAP1 | Homo sapiens | PAR-CLIP | Functional MTI (Weak) | 20371350 |
| hsa-miR-3666 | Homo sapiens | RACGAP1 | Homo sapiens | PAR-CLIP | Functional MTI (Weak) | 20371350 |
| hsa-miR-19a-3p | Homo sapiens | RACGAP1 | Homo sapiens | PAR-CLIP | Functional MTI (Weak) | 20371350 |
| hsa-miR-19b-3p | Homo sapiens | RACGAP1 | Homo sapiens | PAR-CLIP | Functional MTI (Weak) | 20371350 |
| hsa-miR-1278 | Homo sapiens | CCNB1 | Homo sapiens | PAR-CLIP | Functional MTI (Weak) | 20371350 |
| hsa-miR-508-5p | Homo sapiens | CCNB1 | Homo sapiens | PAR-CLIP | Functional MTI (Weak) | 20371350 |
| hsa-miR-508-5p | Homo sapiens | CCNB1 | Homo sapiens | PAR-CLIP | Functional MTI (Weak) | 26701625 |
| hsa-miR-578 | Homo sapiens | AURKA | Homo sapiens | PAR-CLIP | Functional MTI (Weak) | 20371350 |
| hsa-miR-6734-5p | Homo sapiens | AURKA | Homo sapiens | PAR-CLIP | Functional MTI (Weak) | 20371350 |
| hsa-miR-1226-5p | Homo sapiens | AURKA | Homo sapiens | PAR-CLIP | Functional MTI (Weak) | 20371350 |
| hsa-miR-6834-5p | Homo sapiens | AURKA | Homo sapiens | PAR-CLIP | Functional MTI (Weak) | 20371350 |
| hsa-miR-4777-5p | Homo sapiens | AURKA | Homo sapiens | PAR-CLIP | Functional MTI (Weak) | 20371350 |
| hsa-miR-526a | Homo sapiens | AURKA | Homo sapiens | PAR-CLIP | Functional MTI (Weak) | 20371350 |
| hsa-miR-523-5p | Homo sapiens | AURKA | Homo sapiens | PAR-CLIP | Functional MTI (Weak) | 20371350 |
| hsa-miR-522-5p | Homo sapiens | AURKA | Homo sapiens | PAR-CLIP | Functional MTI (Weak) | 20371350 |
| hsa-miR-520c-5p | Homo sapiens | AURKA | Homo sapiens | PAR-CLIP | Functional MTI (Weak) | 20371350 |
| hsa-miR-519c-5p | Homo sapiens | AURKA | Homo sapiens | PAR-CLIP | Functional MTI (Weak) | 20371350 |
| hsa-miR-519b-5p | Homo sapiens | AURKA | Homo sapiens | PAR-CLIP | Functional MTI (Weak) | 20371350 |
| hsa-miR-519a-5p | Homo sapiens | AURKA | Homo sapiens | PAR-CLIP | Functional MTI (Weak) | 20371350 |
| hsa-miR-518f-5p | Homo sapiens | AURKA | Homo sapiens | PAR-CLIP | Functional MTI (Weak) | 20371350 |
| hsa-miR-518e-5p | Homo sapiens | AURKA | Homo sapiens | PAR-CLIP | Functional MTI (Weak) | 20371350 |
| hsa-miR-518d-5p | Homo sapiens | AURKA | Homo sapiens | PAR-CLIP | Functional MTI (Weak) | 20371350 |
| hsa-miR-4721 | Homo sapiens | AURKA | Homo sapiens | PAR-CLIP | Functional MTI (Weak) | 20371350 |
| hsa-miR-3616-3p | Homo sapiens | AURKA | Homo sapiens | PAR-CLIP | Functional MTI (Weak) | 20371350 |
| hsa-miR-581 | Homo sapiens | AURKA | Homo sapiens | PAR-CLIP | Functional MTI (Weak) | 20371350 |
| hsa-miR-892a | Homo sapiens | RACGAP1 | Homo sapiens | PAR-CLIP | Functional MTI (Weak) | 20371350 |
| hsa-miR-3657 | Homo sapiens | RACGAP1 | Homo sapiens | PAR-CLIP | Functional MTI (Weak) | 20371350 |
| hsa-miR-4669 | Homo sapiens | RACGAP1 | Homo sapiens | PAR-CLIP | Functional MTI (Weak) | 20371350 |
| hsa-miR-4468 | Homo sapiens | RACGAP1 | Homo sapiens | PAR-CLIP | Functional MTI (Weak) | 20371350 |
| hsa-miR-6501-3p | Homo sapiens | RACGAP1 | Homo sapiens | PAR-CLIP | Functional MTI (Weak) | 20371350 |
| hsa-miR-2278 | Homo sapiens | RACGAP1 | Homo sapiens | PAR-CLIP | Functional MTI (Weak) | 20371350 |
| hsa-miR-5683 | Homo sapiens | RACGAP1 | Homo sapiens | PAR-CLIP | Functional MTI (Weak) | 20371350 |
| hsa-miR-3977 | Homo sapiens | PRC1 | Homo sapiens | PAR-CLIP | Functional MTI (Weak) | 20371350 |
| hsa-miR-4473 | Homo sapiens | PRC1 | Homo sapiens | PAR-CLIP | Functional MTI (Weak) | 20371350 |
| hsa-miR-3166 | Homo sapiens | PRC1 | Homo sapiens | PAR-CLIP | Functional MTI (Weak) | 20371350 |
| hsa-miR-633 | Homo sapiens | PRC1 | Homo sapiens | PAR-CLIP | Functional MTI (Weak) | 20371350 |
| hsa-miR-2277-3p | Homo sapiens | PRC1 | Homo sapiens | PAR-CLIP | Functional MTI (Weak) | 20371350 |
| hsa-miR-1324 | Homo sapiens | PRC1 | Homo sapiens | PAR-CLIP | Functional MTI (Weak) | 20371350 |
| hsa-miR-512-3p | Homo sapiens | PRC1 | Homo sapiens | PAR-CLIP | Functional MTI (Weak) | 20371350 |
| hsa-miR-6507-5p | Homo sapiens | PRC1 | Homo sapiens | PAR-CLIP | Functional MTI (Weak) | 20371350 |
| hsa-miR-5692a | Homo sapiens | PRC1 | Homo sapiens | PAR-CLIP | Functional MTI (Weak) | 20371350 |
| hsa-miR-520f-3p | Homo sapiens | PRC1 | Homo sapiens | PAR-CLIP | Functional MTI (Weak) | 20371350 |
| hsa-miR-302c-3p | Homo sapiens | PRC1 | Homo sapiens | PAR-CLIP | Functional MTI (Weak) | 20371350 |
| hsa-miR-6819-3p | Homo sapiens | CDKN3 | Homo sapiens | HITS-CLIP | Functional MTI (Weak) | 23824327 |
| hsa-miR-3689d | Homo sapiens | CDKN3 | Homo sapiens | HITS-CLIP | Functional MTI (Weak) | 23824327 |
| hsa-miR-7641 | Homo sapiens | CDKN3 | Homo sapiens | HITS-CLIP | Functional MTI (Weak) | 23824327 |
| hsa-miR-4284 | Homo sapiens | CDKN3 | Homo sapiens | HITS-CLIP | Functional MTI (Weak) | 23824327 |
| hsa-miR-4695-3p | Homo sapiens | CDKN3 | Homo sapiens | HITS-CLIP | Functional MTI (Weak) | 23824327 |
| hsa-miR-4772-3p | Homo sapiens | CDKN3 | Homo sapiens | HITS-CLIP | Functional MTI (Weak) | 23824327 |
| hsa-miR-4638-5p | Homo sapiens | CDKN3 | Homo sapiens | HITS-CLIP | Functional MTI (Weak) | 23824327 |
| hsa-miR-1307-3p | Homo sapiens | CDKN3 | Homo sapiens | HITS-CLIP | Functional MTI (Weak) | 23824327 |
| hsa-miR-1304-3p | Homo sapiens | CDKN3 | Homo sapiens | HITS-CLIP | Functional MTI (Weak) | 23824327 |
| hsa-miR-6741-3p | Homo sapiens | CDKN3 | Homo sapiens | HITS-CLIP | Functional MTI (Weak) | 23824327 |
| hsa-miR-331-3p | Homo sapiens | CDKN3 | Homo sapiens | HITS-CLIP | Functional MTI (Weak) | 23824327 |
| hsa-miR-6810-3p | Homo sapiens | CDKN3 | Homo sapiens | HITS-CLIP | Functional MTI (Weak) | 23824327 |
| hsa-miR-6801-3p | Homo sapiens | CDKN3 | Homo sapiens | HITS-CLIP | Functional MTI (Weak) | 23824327 |
| hsa-miR-6890-3p | Homo sapiens | CDKN3 | Homo sapiens | HITS-CLIP | Functional MTI (Weak) | 23824327 |
| hsa-miR-6836-3p | Homo sapiens | RACGAP1 | Homo sapiens | HITS-CLIP | Functional MTI (Weak) | 23824327 |
| hsa-miR-5001-3p | Homo sapiens | RACGAP1 | Homo sapiens | HITS-CLIP | Functional MTI (Weak) | 23824327 |
| hsa-miR-7158-5p | Homo sapiens | RACGAP1 | Homo sapiens | HITS-CLIP | Functional MTI (Weak) | 23824327 |
| hsa-miR-6829-3p | Homo sapiens | RACGAP1 | Homo sapiens | HITS-CLIP | Functional MTI (Weak) | 23824327 |
| hsa-miR-6791-3p | Homo sapiens | RACGAP1 | Homo sapiens | HITS-CLIP | Functional MTI (Weak) | 23824327 |
| hsa-miR-6778-3p | Homo sapiens | RACGAP1 | Homo sapiens | HITS-CLIP | Functional MTI (Weak) | 23824327 |
| hsa-miR-4772-3p | Homo sapiens | RACGAP1 | Homo sapiens | HITS-CLIP | Functional MTI (Weak) | 23824327 |
| hsa-miR-4772-3p | Homo sapiens | RACGAP1 | Homo sapiens | PAR-CLIP | Functional MTI (Weak) | 27292025 |
| hsa-miR-6787-3p | Homo sapiens | RACGAP1 | Homo sapiens | HITS-CLIP | Functional MTI (Weak) | 23824327 |
| hsa-miR-6787-3p | Homo sapiens | RACGAP1 | Homo sapiens | PAR-CLIP | Functional MTI (Weak) | 27292025 |
| hsa-miR-1304-3p | Homo sapiens | RACGAP1 | Homo sapiens | HITS-CLIP | Functional MTI (Weak) | 23824327 |
| hsa-miR-1304-3p | Homo sapiens | RACGAP1 | Homo sapiens | PAR-CLIP | Functional MTI (Weak) | 27292025 |
| hsa-miR-4726-3p | Homo sapiens | RACGAP1 | Homo sapiens | HITS-CLIP | Functional MTI (Weak) | 23824327 |
| hsa-miR-6764-5p | Homo sapiens | RACGAP1 | Homo sapiens | HITS-CLIP | Functional MTI (Weak) | 23824327 |
| hsa-miR-1915-3p | Homo sapiens | RACGAP1 | Homo sapiens | HITS-CLIP | Functional MTI (Weak) | 23824327 |
| hsa-miR-6840-3p | Homo sapiens | RACGAP1 | Homo sapiens | HITS-CLIP | Functional MTI (Weak) | 23824327 |
| hsa-miR-6736-3p | Homo sapiens | RACGAP1 | Homo sapiens | HITS-CLIP | Functional MTI (Weak) | 23824327 |
| hsa-miR-6736-3p | Homo sapiens | RACGAP1 | Homo sapiens | PAR-CLIP | Functional MTI (Weak) | 27292025 |
| hsa-miR-6890-3p | Homo sapiens | RACGAP1 | Homo sapiens | HITS-CLIP | Functional MTI (Weak) | 23824327 |
| hsa-miR-6890-3p | Homo sapiens | RACGAP1 | Homo sapiens | PAR-CLIP | Functional MTI (Weak) | 27292025 |
| hsa-miR-6516-5p | Homo sapiens | CCNB1 | Homo sapiens | HITS-CLIP | Functional MTI (Weak) | 23313552 |
| hsa-miR-455-3p | Homo sapiens | CCNB1 | Homo sapiens | HITS-CLIP | Functional MTI (Weak) | 23313552 |
| hsa-miR-650 | Homo sapiens | CCNB1 | Homo sapiens | HITS-CLIP | Functional MTI (Weak) | 23313552 |
| hsa-miR-3612 | Homo sapiens | CCNB1 | Homo sapiens | HITS-CLIP | Functional MTI (Weak) | 23313552 |
| hsa-miR-6499-3p | Homo sapiens | CCNB1 | Homo sapiens | HITS-CLIP | Functional MTI (Weak) | 23313552 |
| hsa-miR-2467-3p | Homo sapiens | CCNB1 | Homo sapiens | HITS-CLIP | Functional MTI (Weak) | 23313552 |
| hsa-miR-6086 | Homo sapiens | CCNB1 | Homo sapiens | HITS-CLIP | Functional MTI (Weak) | 23313552 |
| hsa-miR-377-5p | Homo sapiens | CCNB1 | Homo sapiens | HITS-CLIP | Functional MTI (Weak) | 23313552 |
| hsa-miR-4487 | Homo sapiens | CCNB1 | Homo sapiens | HITS-CLIP | Functional MTI (Weak) | 23313552 |
| hsa-miR-4487 | Homo sapiens | CCNB1 | Homo sapiens | PAR-CLIP | Functional MTI (Weak) | 26701625 |
| hsa-miR-558 | Homo sapiens | CCNB1 | Homo sapiens | HITS-CLIP | Functional MTI (Weak) | 23313552 |
| hsa-miR-558 | Homo sapiens | CCNB1 | Homo sapiens | PAR-CLIP | Functional MTI (Weak) | 26701625 |
| hsa-miR-3160-3p | Homo sapiens | CCNB1 | Homo sapiens | HITS-CLIP | Functional MTI (Weak) | 23313552 |
| hsa-miR-3160-3p | Homo sapiens | CCNB1 | Homo sapiens | PAR-CLIP | Functional MTI (Weak) | 26701625 |
| hsa-miR-3192-5p | Homo sapiens | CCNB1 | Homo sapiens | HITS-CLIP | Functional MTI (Weak) | 23313552 |
| hsa-miR-4257 | Homo sapiens | CCNB1 | Homo sapiens | HITS-CLIP | Functional MTI (Weak) | 23313552 |
| hsa-miR-6511a-5p | Homo sapiens | CCNB1 | Homo sapiens | HITS-CLIP | Functional MTI (Weak) | 23313552 |
| hsa-miR-1910-3p | Homo sapiens | CCNB1 | Homo sapiens | HITS-CLIP | Functional MTI (Weak) | 23313552 |
| hsa-miR-203a-3p | Homo sapiens | TOP2A | Homo sapiens | HITS-CLIP | Functional MTI (Weak) | 23313552 |
| hsa-miR-524-5p | Homo sapiens | TOP2A | Homo sapiens | HITS-CLIP | Functional MTI (Weak) | 23313552 |
| hsa-miR-520d-5p | Homo sapiens | TOP2A | Homo sapiens | HITS-CLIP | Functional MTI (Weak) | 23313552 |
| hsa-miR-3668 | Homo sapiens | TOP2A | Homo sapiens | HITS-CLIP | Functional MTI (Weak) | 23313552 |
| hsa-miR-4524b-3p | Homo sapiens | TOP2A | Homo sapiens | HITS-CLIP | Functional MTI (Weak) | 23313552 |
| hsa-miR-7-2-3p | Homo sapiens | TOP2A | Homo sapiens | HITS-CLIP | Functional MTI (Weak) | 23313552 |
| hsa-miR-7-1-3p | Homo sapiens | TOP2A | Homo sapiens | HITS-CLIP | Functional MTI (Weak) | 23313552 |
| hsa-miR-5688 | Homo sapiens | TOP2A | Homo sapiens | HITS-CLIP | Functional MTI (Weak) | 23313552 |
| hsa-miR-495-3p | Homo sapiens | TOP2A | Homo sapiens | HITS-CLIP | Functional MTI (Weak) | 23313552 |
| hsa-miR-561-3p | Homo sapiens | TOP2A | Homo sapiens | HITS-CLIP | Functional MTI (Weak) | 23313552 |
| hsa-miR-654-5p | Homo sapiens | CCNB1 | Homo sapiens | HITS-CLIP | Functional MTI (Weak) | 23313552 |
| hsa-miR-541-3p | Homo sapiens | CCNB1 | Homo sapiens | HITS-CLIP | Functional MTI (Weak) | 23313552 |
| hsa-miR-6729-5p | Homo sapiens | CCNB1 | Homo sapiens | HITS-CLIP | Functional MTI (Weak) | 23313552 |
| hsa-miR-4649-5p | Homo sapiens | CCNB1 | Homo sapiens | HITS-CLIP | Functional MTI (Weak) | 23313552 |
| hsa-miR-6134 | Homo sapiens | CCNB1 | Homo sapiens | HITS-CLIP | Functional MTI (Weak) | 23313552 |
| hsa-miR-6089 | Homo sapiens | CCNB1 | Homo sapiens | HITS-CLIP | Functional MTI (Weak) | 23313552 |
| hsa-miR-148a-5p | Homo sapiens | CENPF | Homo sapiens | Luciferase reporter assay//Western blot | Functional MTI | 27292025 |
| hsa-miR-205-5p | Homo sapiens | CENPF | Homo sapiens | Immunohistochemistry//Luciferase reporter assay//qRT-PCR//Western blot | Functional MTI | 26059417 |
| hsa-miR-128-3p | Homo sapiens | NEK2 | Homo sapiens | Luciferase reporter assay//qRT-PCR//Western blot//DNA methylation analysis | Functional MTI | 24046120 |
| hsa-miR-192-5p | Homo sapiens | RACGAP1 | Homo sapiens | Luciferase reporter assay//qRT-PCR//Microarray//Reporter assay; Microarray; Other | Functional MTI | 19074876 |
| hsa-miR-1290 | Homo sapiens | AURKA | Homo sapiens | PAR-CLIP | Functional MTI (Weak) | 26701625 |
| hsa-miR-1914-5p | Homo sapiens | AURKA | Homo sapiens | PAR-CLIP | Functional MTI (Weak) | 26701625 |
| hsa-miR-25-3p | Homo sapiens | CCNB1 | Homo sapiens | PAR-CLIP | Functional MTI (Weak) | 26701625 |
| hsa-miR-3152-5p | Homo sapiens | AURKA | Homo sapiens | PAR-CLIP | Functional MTI (Weak) | 26701625 |
| hsa-miR-3167 | Homo sapiens | AURKA | Homo sapiens | PAR-CLIP | Functional MTI (Weak) | 26701625 |
| hsa-miR-32-5p | Homo sapiens | CCNB1 | Homo sapiens | PAR-CLIP | Functional MTI (Weak) | 26701625 |
| hsa-miR-3611 | Homo sapiens | AURKA | Homo sapiens | PAR-CLIP | Functional MTI (Weak) | 26701625 |
| hsa-miR-363-3p | Homo sapiens | CCNB1 | Homo sapiens | PAR-CLIP | Functional MTI (Weak) | 26701625 |
| hsa-miR-367-3p | Homo sapiens | CCNB1 | Homo sapiens | PAR-CLIP | Functional MTI (Weak) | 26701625 |
| hsa-miR-378j | Homo sapiens | AURKA | Homo sapiens | PAR-CLIP | Functional MTI (Weak) | 26701625 |
| hsa-miR-4293 | Homo sapiens | CCNB1 | Homo sapiens | PAR-CLIP | Functional MTI (Weak) | 26701625 |
| hsa-miR-4325 | Homo sapiens | CCNB1 | Homo sapiens | PAR-CLIP | Functional MTI (Weak) | 26701625 |
| hsa-miR-4755-3p | Homo sapiens | AURKA | Homo sapiens | PAR-CLIP | Functional MTI (Weak) | 26701625 |
| hsa-miR-4774-3p | Homo sapiens | AURKA | Homo sapiens | PAR-CLIP | Functional MTI (Weak) | 26701625 |
| hsa-miR-4782-5p | Homo sapiens | AURKA | Homo sapiens | PAR-CLIP | Functional MTI (Weak) | 26701625 |
| hsa-miR-518c-5p | Homo sapiens | AURKA | Homo sapiens | PAR-CLIP | Functional MTI (Weak) | 26701625 |
| hsa-miR-5706 | Homo sapiens | AURKA | Homo sapiens | PAR-CLIP | Functional MTI (Weak) | 26701625 |
| hsa-miR-6512-3p | Homo sapiens | CCNB1 | Homo sapiens | PAR-CLIP | Functional MTI (Weak) | 26701625 |
| hsa-miR-660-3p | Homo sapiens | AURKA | Homo sapiens | PAR-CLIP | Functional MTI (Weak) | 26701625 |
| hsa-miR-6720-5p | Homo sapiens | CCNB1 | Homo sapiens | PAR-CLIP | Functional MTI (Weak) | 26701625 |
| hsa-miR-6778-3p | Homo sapiens | AURKA | Homo sapiens | PAR-CLIP | Functional MTI (Weak) | 26701625 |
| hsa-miR-6791-3p | Homo sapiens | AURKA | Homo sapiens | PAR-CLIP | Functional MTI (Weak) | 26701625 |
| hsa-miR-6829-3p | Homo sapiens | AURKA | Homo sapiens | PAR-CLIP | Functional MTI (Weak) | 26701625 |
| hsa-miR-6836-3p | Homo sapiens | AURKA | Homo sapiens | PAR-CLIP | Functional MTI (Weak) | 26701625 |
| hsa-miR-6839-5p | Homo sapiens | AURKA | Homo sapiens | PAR-CLIP | Functional MTI (Weak) | 26701625 |
| hsa-miR-6849-3p | Homo sapiens | CCNB1 | Homo sapiens | PAR-CLIP | Functional MTI (Weak) | 26701625 |
| hsa-miR-760 | Homo sapiens | AURKA | Homo sapiens | PAR-CLIP | Functional MTI (Weak) | 26701625 |
| hsa-miR-766-3p | Homo sapiens | CCNB1 | Homo sapiens | PAR-CLIP | Functional MTI (Weak) | 26701625 |
| hsa-miR-7703 | Homo sapiens | CCNB1 | Homo sapiens | PAR-CLIP | Functional MTI (Weak) | 26701625 |
| hsa-miR-876-5p | Homo sapiens | AURKA | Homo sapiens | PAR-CLIP | Functional MTI (Weak) | 26701625 |
| hsa-miR-92b-3p | Homo sapiens | CCNB1 | Homo sapiens | PAR-CLIP | Functional MTI (Weak) | 26701625 |
| hsa-miR-122-5p | Homo sapiens | RACGAP1 | Homo sapiens | PAR-CLIP | Functional MTI (Weak) | 27292025 |
| hsa-miR-1279 | Homo sapiens | NEK2 | Homo sapiens | PAR-CLIP | Functional MTI (Weak) | 27292025 |
| hsa-miR-143-5p | Homo sapiens | RACGAP1 | Homo sapiens | PAR-CLIP | Functional MTI (Weak) | 27292025 |
| hsa-miR-186-5p | Homo sapiens | NEK2 | Homo sapiens | PAR-CLIP | Functional MTI (Weak) | 27292025 |
| hsa-miR-215-3p | Homo sapiens | RACGAP1 | Homo sapiens | PAR-CLIP | Functional MTI (Weak) | 27292025 |
| hsa-miR-3135b | Homo sapiens | RACGAP1 | Homo sapiens | PAR-CLIP | Functional MTI (Weak) | 27292025 |
| hsa-miR-3652 | Homo sapiens | RACGAP1 | Homo sapiens | PAR-CLIP | Functional MTI (Weak) | 27292025 |
| hsa-miR-4430 | Homo sapiens | RACGAP1 | Homo sapiens | PAR-CLIP | Functional MTI (Weak) | 27292025 |
| hsa-miR-4652-3p | Homo sapiens | NEK2 | Homo sapiens | PAR-CLIP | Functional MTI (Weak) | 27292025 |
| hsa-miR-4668-5p | Homo sapiens | NEK2 | Homo sapiens | PAR-CLIP | Functional MTI (Weak) | 27292025 |
| hsa-miR-4695-3p | Homo sapiens | RACGAP1 | Homo sapiens | PAR-CLIP | Functional MTI (Weak) | 27292025 |
| hsa-miR-4778-5p | Homo sapiens | NEK2 | Homo sapiens | PAR-CLIP | Functional MTI (Weak) | 27292025 |
| hsa-miR-500b-3p | Homo sapiens | RACGAP1 | Homo sapiens | PAR-CLIP | Functional MTI (Weak) | 27292025 |
| hsa-miR-504-3p | Homo sapiens | RACGAP1 | Homo sapiens | PAR-CLIP | Functional MTI (Weak) | 27292025 |
| hsa-miR-564 | Homo sapiens | RACGAP1 | Homo sapiens | PAR-CLIP | Functional MTI (Weak) | 27292025 |
| hsa-miR-5693 | Homo sapiens | RACGAP1 | Homo sapiens | PAR-CLIP | Functional MTI (Weak) | 27292025 |
| hsa-miR-5698 | Homo sapiens | RACGAP1 | Homo sapiens | PAR-CLIP | Functional MTI (Weak) | 27292025 |
| hsa-miR-6499-3p | Homo sapiens | RACGAP1 | Homo sapiens | PAR-CLIP | Functional MTI (Weak) | 27292025 |
| hsa-miR-6773-3p | Homo sapiens | RACGAP1 | Homo sapiens | PAR-CLIP | Functional MTI (Weak) | 27292025 |
| hsa-miR-6879-3p | Homo sapiens | RACGAP1 | Homo sapiens | PAR-CLIP | Functional MTI (Weak) | 27292025 |
| hsa-miR-891a-3p | Homo sapiens | RACGAP1 | Homo sapiens | PAR-CLIP | Functional MTI (Weak) | 27292025 |
