# Supplementary material for: Integrative analysis identifies key mRNA biomarkers for diagnosis, prognosis, and therapeutic targets of HCV-associated hepatocellular carcinoma
Source: Aging (Albany NY). 2021 May 4;13(9):12865–95. doi: 10.18632/aging.202957 (PMC8148482; doi:10.18632/aging.202957)
Supplement: Supplementary Table 6 [file aging-13-202957-s007.doc]

## Supplementary Table 6. Anti-neoplastic drugs predicted by DGIdb.

| **Gene** | **Drug** | **Interaction Type & Directionality** | **Sources** | **PMIDs** | **Query Score** | **Interaction Score** |
| --- | --- | --- | --- | --- | --- | --- |
| NEK2 | PAZOPANIB | n/a | DTC | - | 0.19 | 0.07 |
| NEK2 | PALBOCICLIB | n/a | DTC | - | 0.09 | 0.07 |
| NEK2 | FOSTAMATINIB | inhibitor | DrugBank | 26516587 | 0.06 | 0.02 |
| TOP2A | FINAFLOXACIN | inhibitor | DrugBank | 25808831 | 8.71 | 1.17 |
| TOP2A | VALRUBICIN | inhibitor | DrugBank|TdgClinicalTrial|ChemblInteractions|TEND | 11752352|16019763 | 8.71 | 1.17 |
| TOP2A | DEXRAZOXANE | inhibitor | DrugBank|NCI | 11179439|17652819|10194547|11046078|12911317|17115008|11752352|11984069|11332155 | 7.99 | 1.07 |
| TOP2A | TENIPOSIDE | inhibitor | DrugBank|TdgClinicalTrial|ChemblInteractions|NCI|TEND | 8702194|16271071|17361331|17514873|11752352|16480143|9426516 | 6.53 | 0.88 |
| TOP2A | MOXIFLOXACIN | inhibitor | DrugBank | 20802486|20596674|11752352 | 5.81 | 0.78 |
| TOP2A | ENOXACIN | inhibitor | DrugBank | 18471102|11752352|10089819 | 5.81 | 0.78 |
| TOP2A | ETOPOSIDE | inhibitor | DTC|DrugBank|TdgClinicalTrial|ChemblInteractions|NCI|TEND|GuideToPharmacology | 25466187|20006518|18258442|8823806|22867019|25240702|26291037|25003995|26216018|26292628|23360284|16271071|23920485|21435753|22867097|16759114|11678653|19386396|24931277|23566520|17361331|25922181|25941559|24507920|24775914|9485461|23353750|25815139|16309315|24012683|19691293|25800514|21644529|22620261|25945730|24334150|17514873|8870683|23711769|11752352|20863598|24095018|26264845|25799376|22364746|16377807|9494516|23968711|18816045|24326278|19783445|9426516 | 4.94 | 0.66 |
| TOP2A | AMSACRINE | inhibitor | DTC|DrugBank|NCI | 1322791|8823806|10691026|8519659|19155103|22537681|17911018|8632768|19725581|11006484|11716434|11752352|25626146|11473732|1311390 | 4.36 | 0.59 |
| TOP2A | LOMEFLOXACIN | inhibitor | DrugBank | 11752352 | 2.9 | 0.39 |
| TOP2A | MITOXANTRONE | inhibitor | DrugBank|TdgClinicalTrial|NCI|TEND|GuideToPharmacology | 10451375|11004693|18687447|11752352|9631585|9494516|11278845|9426516 | 2.76 | 0.19 |
| TOP2A | PEFLOXACIN | inhibitor | DrugBank | 11752352 | 2.18 | 0.29 |
| TOP2A | NORFLOXACIN | inhibitor | DrugBank | 11752352 | 2.18 | 0.29 |
| TOP2A | PODOFILOX | inhibitor | DrugBank|TdgClinicalTrial|TEND | 16061385|1334447|10783066|11752352|1845848|1331331 | 1.7 | 0.23 |
| TOP2A | CIPROFLOXACIN | inhibitor | DrugBank | 3015015|1323952|11752352 | 1.58 | 0.21 |
| TOP2A | IDARUBICIN | inhibitor | DrugBank|TdgClinicalTrial|TEND | 10203104|8036155|12034365|11836027|11752352|10523799 | 1.09 | 0.15 |
| TOP2A | EPIRUBICIN | inhibitor | DrugBank|TdgClinicalTrial|TEND|PharmGKB | 14728934|16234514|17639997 | 1.02 | 0.14 |
| TOP2A | DOXORUBICIN | inhibitor | DTC|DrugBank|TdgClinicalTrial|ClearityFoundationClinicalTrial|TEND|GuideToPharmacology | 21388138|17016621|17578914|17010609|17351394|26211460|11752352|20170164|17089011|22276998 | 0.73 | 0.1 |
| TOP2A | OFLOXACIN | inhibitor | DrugBank | 2847647 | 0.73 | 0.1 |
| TOP2A | DAUNORUBICIN | inhibitor | DTC|DrugBank|TdgClinicalTrial|NCI | 22260166|1963303|6380596|9494516 | 0.52 | 0.07 |
| TOP2A | DACTINOMYCIN | inhibitor | DrugBank | 7756657 | 0.51 | 0.07 |
| TOP2A | VINCRISTINE | n/a | NCI | 9494516 | 0.15 | 0.02 |
| TOP2A | PACLITAXEL | n/a | TdgClinicalTrial | - | 0.09 | 0.01 |
| TOP2A | FLUOROURACIL | n/a | PharmGKB | - | 0.08 | 0.01 |
| RACGAP1 | MITOXANTRONE | n/a | DTC | - | 0.21 | 0.02 |
| AURKA | PAZOPANIB | n/a | DTC | - | 0.19 | 0.02 |
| AURKA | PACLITAXEL | n/a | CIViC | 12559175 | 0.17 | 0.02 |
| AURKA | FLUOROURACIL | n/a | CIViC | 25924824 | 0.17 | 0.02 |
| AURKA | TAMOXIFEN | n/a | CIViC | 24166501 | 0.13 | 0.03 |
| AURKA | CISPLATIN | n/a | CIViC | 25924824|25082261 | 0.09 | 0.02 |
| AURKA | FOSTAMATINIB | inhibitor | DrugBank | 26516587 | 0.06 | 0.01 |
| AURKA | SORAFENIB | n/a | DTC | - | 0.05 | 0.01 |
